# The SPF27 Homologue Num1 Connects Splicing and Kinesin 1-Dependent Cytoplasmic Trafficking in *Ustilago maydis*

**DOI:** 10.1371/journal.pgen.1004046

**Published:** 2014-01-02

**Authors:** Nikola Kellner, Kai Heimel, Theresa Obhof, Florian Finkernagel, Jörg Kämper

**Affiliations:** 1 Karlsruhe Institute of Technology, Institute for Applied Biosciences, Department of Genetics, Karlsruhe, Germany; 2 Georg-August-University-Göttingen, Institute for Microbiology and Genetics, Microbial Cell Biology, Göttingen, Germany; 3 Institute for Molecular Biology and Tumor Research, Marburg, Germany; Duke University Medical Center, United States of America

## Abstract

The conserved NineTeen protein complex (NTC) is an integral subunit of the spliceosome and required for intron removal during pre-mRNA splicing. The complex associates with the spliceosome and participates in the regulation of conformational changes of core spliceosomal components, stabilizing RNA-RNA- as well as RNA-protein interactions. In addition, the NTC is involved in cell cycle checkpoint control, response to DNA damage, as well as formation and export of mRNP-particles. We have identified the Num1 protein as the homologue of SPF27, one of NTC core components, in the basidiomycetous fungus *Ustilago maydis*. Num1 is required for polarized growth of the fungal hyphae, and, in line with the described NTC functions, the *num1* mutation affects the cell cycle and cell division. The *num1* deletion influences splicing in *U. maydis* on a global scale, as RNA-Seq analysis revealed increased intron retention rates. Surprisingly, we identified in a screen for Num1 interacting proteins not only NTC core components as Prp19 and Cef1, but several proteins with putative functions during vesicle-mediated transport processes. Among others, Num1 interacts with the motor protein Kin1 in the cytoplasm. Similar phenotypes with respect to filamentous and polar growth, vacuolar morphology, as well as the motility of early endosomes corroborate the genetic interaction between Num1 and Kin1. Our data implicate a previously unidentified connection between a component of the splicing machinery and cytoplasmic transport processes. As the *num1* deletion also affects cytoplasmic mRNA transport, the protein may constitute a novel functional interconnection between the two disparate processes of splicing and trafficking.

## Author Summary

In eukaryotic cells, nascent mRNA is processed by splicing to remove introns and to join the exon sequences. The processed mRNA is then transported out of the nucleus and employed by ribosomes to synthesize proteins. Splicing is achieved by the spliceosome and associated protein complexes, among them the so-called NineTeen complex (NTC). We have identified the Num1 protein as one of the core components of the NTC in the fungus *Ustilago maydis*, and could show that it is required for polarized growth of the filamentous fungal cells. Consistent with the NTC function, cells with a *num1*-deletion show reduced splicing of mRNA. Moreover, we uncover a novel cytoplasmic function of the Num1 protein: It physically interacts with the microtubule-associated Kinesin 1 motor protein, and phenotypic analyses corroborate that both proteins are functionally connected. Our findings reveal a yet unidentified role of a global splicing factor during intracellular trafficking processes. A possible connection between these disparate mechanisms presumably resides in mRNA-export out of the nucleus and/or the transport of mRNA within the cytoplasm.

## Introduction

The basidiomycete *Ustilago maydis* is the causal agent of the smut disease on corn (*Zea mays*). During its life cycle, *U. maydis* displays yeast-like, haploid cells that divide by budding, and dikaryotic cells that grow as filamentous hyphae. The filamentous stage is initiated by fusion of two yeast-like cells (sporidia) and marks the onset of the biotrophic stage in which the fungus depends on its host plant for propagation. The switch to polarized growth to form the elongated filament is indispensable for the successful infection of the host plant.

The regulatory circuits that underlie the dimorphic switch and concurrent pathogenic development in *U. maydis* have been well studied within the past years. Similar to other basidiomycetes, a heterodimeric complex of two homeodomain transcription factors, both encoded by the *b*-mating type locus, represents the major regulatory instance. The two proteins, termed bE and bW in *U. maydis*, form a functional heterodimer, but only if they originate from different *b*-alleles (e.g. bE1 and bW2). Hyphae formed upon activation of the *b*-pathway grow unipolar, but only the tip compartment is filled with cytoplasm and contains the two genetically distinct nuclei, while the distal part of the hypha is composed of empty segments separated by evenly spaced retraction septa. Simultaneously with the switch towards filamentous growth, cells become arrested in the G2 phase of the cell cycle. Only after the penetration of the plant surface, this cell cycle block is released, and the “true” filament with multiple septated compartments is developed [for recent review, see 1].

The bE/bW-heterodimer orchestrates a hierarchic, multi-layered transcriptional network. Only few *b*-responsive genes are direct targets of the bE/bW heterodimer. The majority of the genes is regulated via the b-dependently induced C2H2 zinc-finger transcription factor Rbf1. Rbf1 is the master regulator for several other transcription factors that coordinate expression of multiple genes associated with cell cycle coordination, morphogenesis and pathogenic development. Deletion of *rbf1* prevents the formation of the *b*-dependent filaments, and ectopic expression of *rbf1* is sufficient to induce the dimorphic transition. Thus, *rbf1* is both sufficient as well as required for the switch from budding to polarized filamentous growth [2].

Prerequisite for the growth of the filamentous hyphae is the establishment and maintenance of a defined axis of polarity. The filaments expand by polar tip growth, which is dependent on long-distance transport towards the growth cones at the cell apices. This directed transport is facilitated by arrays of polarized microtubules and a highly conserved set of microtubule-dependent kinesin and dynein motor proteins [reviewed in 3]. The cellular cargos that rely on microtubule-based transport include endosomes, peroxisomes and nuclei, but also mRNA, which, as shown recently in *U. maydis*, is as well instrumental for establishment and maintenance of polarity [4]–[6]. The latter, however, is not a direct microtubule-based cargo, but passively travels on endosomes [7]. In *U. maydis*, transport is mainly mediated through the concerted action of the plus-end directed UNC104-like Kinesin-3 motor protein Kin3, which moves endosomes in both directions within the cell along an array of antiparallel microtubules, and the minus-end directed dynein motor protein Dyn1/2 [8]–[11]. The minus-end directed Dyn1/2 is particularly important at the poles of filaments, where unipolar microtubules, with their plus-ends directed to the tip, extend from the antiparallel array [9], [12]. The conventional kinesin motor protein Kin1 additionally contributes to the establishment of hyphal morphology by transporting Dynein in the direction of the microtubule plus-ends within the hyphal apex where a loading zone for the retrograde transport processes is established [9], [12]. Other known cargos for the Kin1 motor protein include membranous structures; Kin1 was previously described to be involved in organelle transport [13] and to foster the transport of secretory vesicles into the growing tip [14]. More recently, transport of the fungal-specific class-17 myosin Mcs1 was shown to dependent directly on Kin1. Mcs1 is attached to vesicles and contains an N-terminal myosin motor domain fused to a chitin synthase region. Anterograde trafficking of Mcs1-positive vesicles requires both microtubules and filamentous actin and depends on Myosin-5 and Kinesin-1, which cooperate in delivering vesicles to the sites of exocytosis [15]–.

In an effort to achieve a better understanding of the complex processes required for the establishment and maintenance of the dikaryotic hyphae in *U. maydis*, we employed a candidate approach with genes that, based on function or phenotype in other systems, were anticipated to be involved in nuclear migration and determination of cell polarity. Here, we describe the molecular characterization of one of these genes, *num1*. The *num1* mutation has originally been identified in the basidiomycete *Coprinopsis cinerea* in a screen for mutants affected in the nuclear migration during the formation of the heterokaryotic hyphae [18].

We show now that Num1 is a homologue of SPF27, one of the core components of the highly conserved Prp19/CDC5L or NineTeen (NTC) splicing complex [19].

Unexpectedly, we identified the conventional Kinesin 1 motor protein Kin1 [13], [20] to interact with Num1. Similar phenotypes of *Δnum1* and *Δkin1* hyphae corroborate a functional interconnection between the two proteins. Our data implicate a previously unidentified connection between a component of the splicing machinery and cytoplasmic (Kin1-dependent) transport processes in *U. maydis*.

## Results

### Num1, a conserved BCAS2/SPF27-like protein, is required for polar growth and septum formation

In the basidiomycete *Coprinopsis cinerea, the num1* gene was identified in a screen for mutants affected in nuclear migration during the initial phase of sexual development [18]. Unlike *U. maydis, C. cinerea* grows strictly as a filament, and mating is initiated by fusion of two different haploid hyphae harboring compatible alleles of the mating type loci. The dikaryotic filament is then generated upon migration of “donor”-nuclei into the “acceptor”-mycelium. The *num1* mutation results in strains that are still able to donate nuclei, but fail to accept nuclei in compatible mating reactions [18]. To examine whether the gene has a conserved function in *U. maydis* with respect to nuclear migration, we set out to analyze the potential *U. maydis* homologue. The *U. maydis* predicted protein Um01682 (MIPS *Ustilago maydis* Database (MUMDB), http://mips.helmholtz-muenchen.de/genre/proj/ustilago/) exhibits 43% identity and 63% similarity with the *C. cinerea* Num1 protein, and 33% identity to the human SPF27 protein (Figure S1). SPF27 homologues are found in all eukaryotic clades, with the exception of the Saccharomycetales (Figure S2). SPF27 is part of the Prp19/CDC5L complex, which is an integral component of active spliceosomes and required for intron removal [21]. Similar to the SPF27 homologues, the *U. maydis* protein harbors a BCAS2 domain (breast carcinoma amplified sequence 2) with a so far unknown function and a classical, basically charged nuclear localization signal (Figure S1) [22].

To determine the function of Num1 in *U. maydis*, the gene was deleted in the strains FB1, FB2, SG200 and AB31. FB1 (*a1 b1*) and FB2 (*a2 b2*) are compatible wild-type strains that, upon mating, form filaments on solid media supplemented with charcoal. SG200 (*a1 mfa2 bE1 bW2*) forms such filaments without a mating partner, as the strain harbors a compatible combination of the *bE1* and *bW2* genes [23]. For both FB1/FB2 and SG200, cell division upon filament formation is stalled in axenic culture, and hyphal proliferation depends on the plant host. Polarized growth in axenic culture can be best monitored in strain AB31 (*a1 P_crg1_:bE1/P_crg1_:bW2*), which harbors a set of compatible *bE1* and *bW2* genes under control of the arabinose-responsive *P_crg1_*-promoter [24]. Upon arabinose-induced expression of *bE1/bW2*, AB31 switches from budding- to polarized growth and forms long filaments reminiscent of those formed after fusion of compatible FB1 and FB2 sporidia [2], [24].

**Figure 1:**
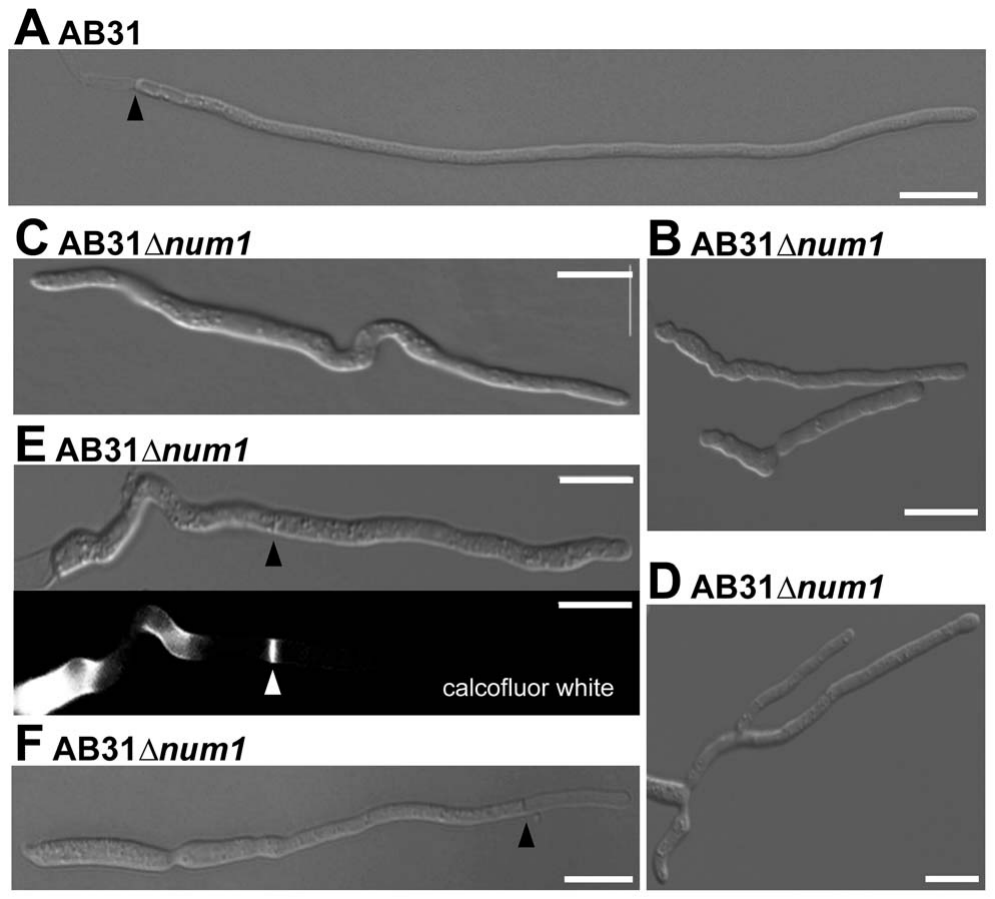
Num1 is required for polar growth and septum formation. Filament formation of AB31 wild-type and AB31*Δnum1* deletion strains was monitored 12–14 hours upon induction of the bE1/bW2 heterodimer. (**A**) AB31 wild-type cells grow as long, straight filaments; only the tip compartment is filled with cytoplasm, separated from highly vacuolated distal compartments by a septum (arrowhead). (**B**) AB31*Δnum1* hyphae grow shorter and more curved. In AB31*Δnum1*, bipolar (**C**) and branched (**D**) hyphae are observed frequently. (**E**) Septa of AB31*Δnum1* are often formed within the compartment filled with cytoplasm (arrowheads denote delocalized septum; in lower panel, Calcofluor White staining was used to visualize the septum). (**F**) In approx. 2.5% of hyphae delocalized septa (arrowhead) lead to empty tip compartments. Scale bars: 10 µm.

Yeast-like cells of AB31*Δnum1*- and SG200*Δnum1* showed no obvious phenotype when grown in axenic culture in complete or minimal media (Figure S3). In plant infection assays, the mixture of the compatible strains FB1*Δnum1* and FB2*Δnum1* as well as SG200*Δnum1* showed reduced disease symptoms compared to the respective wild-type strains; both the number of plants with tumors as well as the size of tumors decreased (Figure S3). However, microscopic observation of hyphae after plant infection did not reveal major morphological differences to wild-type strains (Figure S3). The cell-cell fusion of compatible sporidia to generate the dikaryotic hyphae, which can be visualized by the appearance of “fuzzy” colonies on charcoal containing media, is not altered by the *num1* mutation (Figure S3). However, the phenotype of conjugation hyphae upon treatment with synthetic pheromone as well as the phenotype of the dikaryotic hyphae showed considerable alterations from wild-type strains. Pheromone treatment of *Δnum1* strains led to the formation of branched conjugation tubes that are never observed in wild-type strains. Dikaryotic hyphae of *Δnum1* strains appeared thicker, branched, and fused sporidia displayed bipolar growth; in wild-type strains, however, the fusion event is initiated only from a single cell pole (Figures S4 and S5). To analyze the phenotype of the hyphae in more detail, we employed strain AB31 and induced the formation of the filament via the expression of *bE1/bW2*. Similar to the dikaryotic hyphae, AB31*Δnum1* filaments displayed severe alterations. 95% of the filaments were significantly shorter than wild-type AB31 filaments (average length of 68.9 µm compared to 121.7 µm, t-test: p = 6.35×10^−46^). Filament formation upon *b*-induction in AB31 is initiated at one of the cell poles of the sporidium; the resulting hyphae grow unbranched and strictly unipolar (Figure 1A). In AB31*Δnum1*, hyphae generally exhibited an irregular and curved morphology (Figure 1B, Table 1). In 18% of the sporidia, hyphae initiated at both cell poles (bipolar, Figure 1C, Table 1), and 4.4% exhibited a branched morphology (N = 461) (Figure 1D, Table 1). In addition, 21.2% of the AB31*Δnum1* filaments had delocalized septa, and 2.4% of the hyphae generated empty compartments at the cell tips (Figure 1E and F, Table 1). Septa in AB31*Δnum1* could be visualized by different chitin-stains, as Calcofluor [25], Congo Red [26] or fluorescein-conjugated wheat germ agglutinin (WGA) [27] (Figures 1E and S6; see also Figure S6 legend). However, in contrast to septa in the dikaryotic hyphae, septa in AB31*Δnum1* were not always visible in the DIC channel, and staining with fluorescein-WGA revealed extensive chitin accumulations or ring-like chitin structures throughout the hyphae, indicating that these septa or septa-like structures may be structurally different from wild-type septa.

**Table 1:** Quantification of phenotypes in AB31 and AB31*Δnum1* 12 hours after the induction of the bE/bW-heterodimer.

	AB31*	AB31*Δnum1* *
long and straight hyphae	92%±0.010	4.7%±0.029
irregular growing hyphae (short, curvy)	8%±0.015	49.13%±0.029
bipolar	0%	18.13%±0.026
branched	0%	4.4%±0.011
delocalized septa	0%	21.2%±0.005
empty compartments at hyphal tips	0%	2.43%±0.039
	N = 375	N = 461
Ø length of hyphae**	121.7 µm±15.63 µm	68.9 µm±15.37 µm
	N = 75	N = 75

* Mean values from three independent biological replicates are shown.

** t-test p = 6.35×10^−46^.

To ensure that the observed phenotype is due to the *num1* deletion, a construct harboring a *num1:eGFP* fusion gene under control of the arabinose inducible *P_crg1_*-promoter was introduced in single copy into the *cbx* locus. Strain UNK220 (AB31*Δnum1 P_crg1_:num1:egfp*) was grown in arabinose-containing media to induce both the bE1/bW2 heterodimer as well as the *num1:eGFP* gene; the induced filaments were indistinguishable from that of the wild-type AB31 strain (Figure S7).

### Num1 is a component of the highly conserved spliceosome-associated Prp19/CDC5L-complex

**Figure 2:**
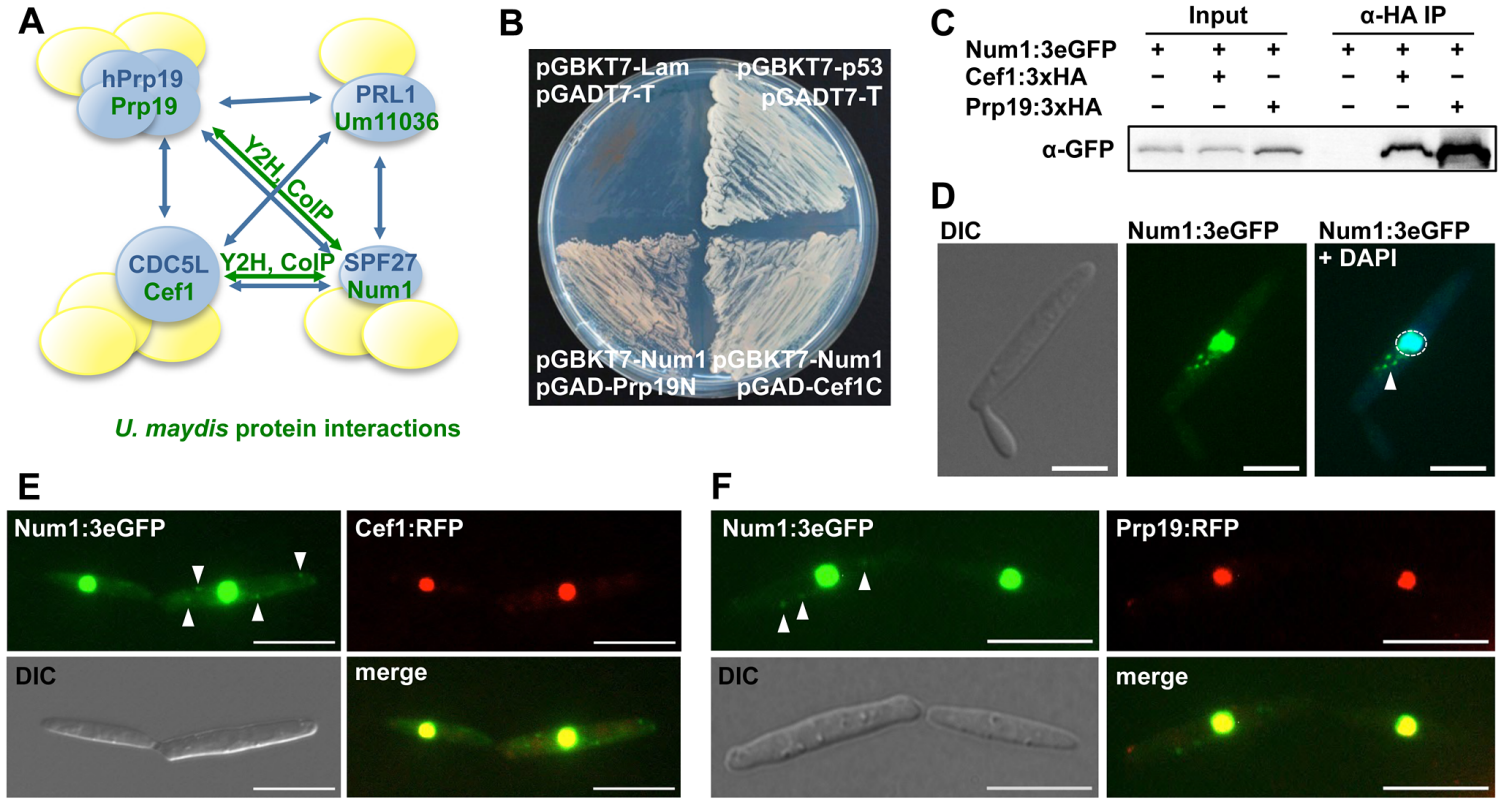
Num1 interacts with components of the Prp19/Cdc5L complex. (**A**) Schematic representation of the protein-protein interactions (blue arrows) within the human hPrp19/CDC5L-complex [21]. Proteins depicted in blue represent the core complex, additional components (yellow) are associated with individual proteins. Green arrows indicate *U. maydis* protein interactions identified in this study. (**B**) The Prp19 N-terminus and the Cef1 C-terminus interact with the full length Num1 protein in a yeast two-hybrid analysis. p53/SV40 T-antigen and Lamin/SV40 T-antigen (Clontech) serve as positive and negative control, respectively. (**C**) *In vivo* co-immunoprecipitation of HA-tagged Prp19 and Cef1 and 3eGFP-tagged Num1. α-HA coupled agarose beads were used to precipitate Prp19 and Cef1, respectively. The Num1 protein was detected on a Western blot using an α-GFP antibody. The negative control shows no unspecific binding of the Num1 protein to α-HA agarose. (**D**) Subcellular localization of the Num1:3eGFP fusion protein. In addition to the nuclear localization of Num1 (enclosed by dashed circle), a cytoplasmic distribution to distinct foci can be observed (arrowhead). Scale bar: 5 µm. (**E, F**) Num1:3eGFP as well as Cef1:RFP and Prp19:RFP were co-expressed in AB31 under control of their native promoters. Num1 co-localizes with both Cef1 and Prp19 in the nuclei. Additional cytoplasmic Num1:3eGFP foci are indicated by arrowheads. Scale bars: 10 µm.

Num1 has striking similarities to the human BCAS2/SPF27 protein that has been identified as one of the four core components (Prp19, PLR1, CDC5L and SPF27) of the Prp19/CDC5L spliceosome-associated complex, commonly termed NineTeen Complex (NTC) (Figure 2A). To assess whether Num1 is a structural component of the putative NTC complex in *U. maydis*, we employed a directed yeast two-hybrid approach using the full-length Num1 protein as bait as well as full-length and C- and N-terminal versions of the Prp19 (Um10027) and CDC5L-homologues (termed Cef1, Um04411) as prey, respectively. Consistent with the previously identified interactions in *S. pombe* [28], Num1 interacts with the Prp19 N-terminus as well as the Cef1 C-terminus (Figure 2B). The interaction of Num1 with Prp19 and Cef1 was verified by *in vivo* co-immunoprecipitation. *num1* was replaced by an eGFP-tagged version of the gene in strain AB31 (*a2, P_crg_:bW2, bE1*), and additionally *prp19* or *cef1* were exchanged with HA-tagged versions, resulting in strains UNK200 (AB31 *num1:3×eGFP:hyg^R^, prp19:3×HA:cbx^R^*) and UMO8 (AB31 *num1:3×eGFP:hyg^R^, cef1:3×HA:cbx^R^*), respectively. All genes were introduced via homologous recombination into their native loci to ensure native protein levels. All fusion proteins were shown to be functional (Supporting Text S1, Figure S8). By use of an anti-HA-antibody, Num1:3eGFP was specifically co-precipitated with both Prp19:HA and Cef1:HA in protein extracts from strains UNK200 and UMO8 (Figure 2C). The interaction of Num1 with Prp19 and Cef1 was further corroborated by their localization. Num1:3eGFP and Prp19:RFP or Cef1:RFP were co-expressed in strain AB31, and all proteins co-localized in the nuclei (Figure 2E and F). While for Prp19:RFP and Cef1:RFP the fluorescence signal was exclusively localized in the nucleus, Num1:3eGFP was also detected in the cytoplasm (Figure 2D). Transformation of AB31*Δnum1* with a *num1:eGFP* derivative harboring a mutated nuclear localization sequence (KKRK to AAAA) did not lead to complementation of the *Δnum1* phenotype, and the fusion protein localized predominantly in the cytoplasm (Figure S7), which implies that nuclear localization of Num1 is required for function of the protein.

In addition to their function in mRNA splicing, NTC and NTC-associated proteins are known to be involved in several other cellular processes. In *S. pombe* and *S. cerevisiae*, Cdc5 and Cef1p, respectively, were originally identified as cell division cycle mutants that are required for G2 progression and mitotic entry [29]–[31], and Prp19p has been isolated in *S. cerevisiae* in a screen for mutants conferring sensitivity to DNA damaging agents [32]. It has been proposed that Prp19p homologues play a direct role in DNA damage repair that is conserved from yeast to human [33]–[38].

**Figure 3:**
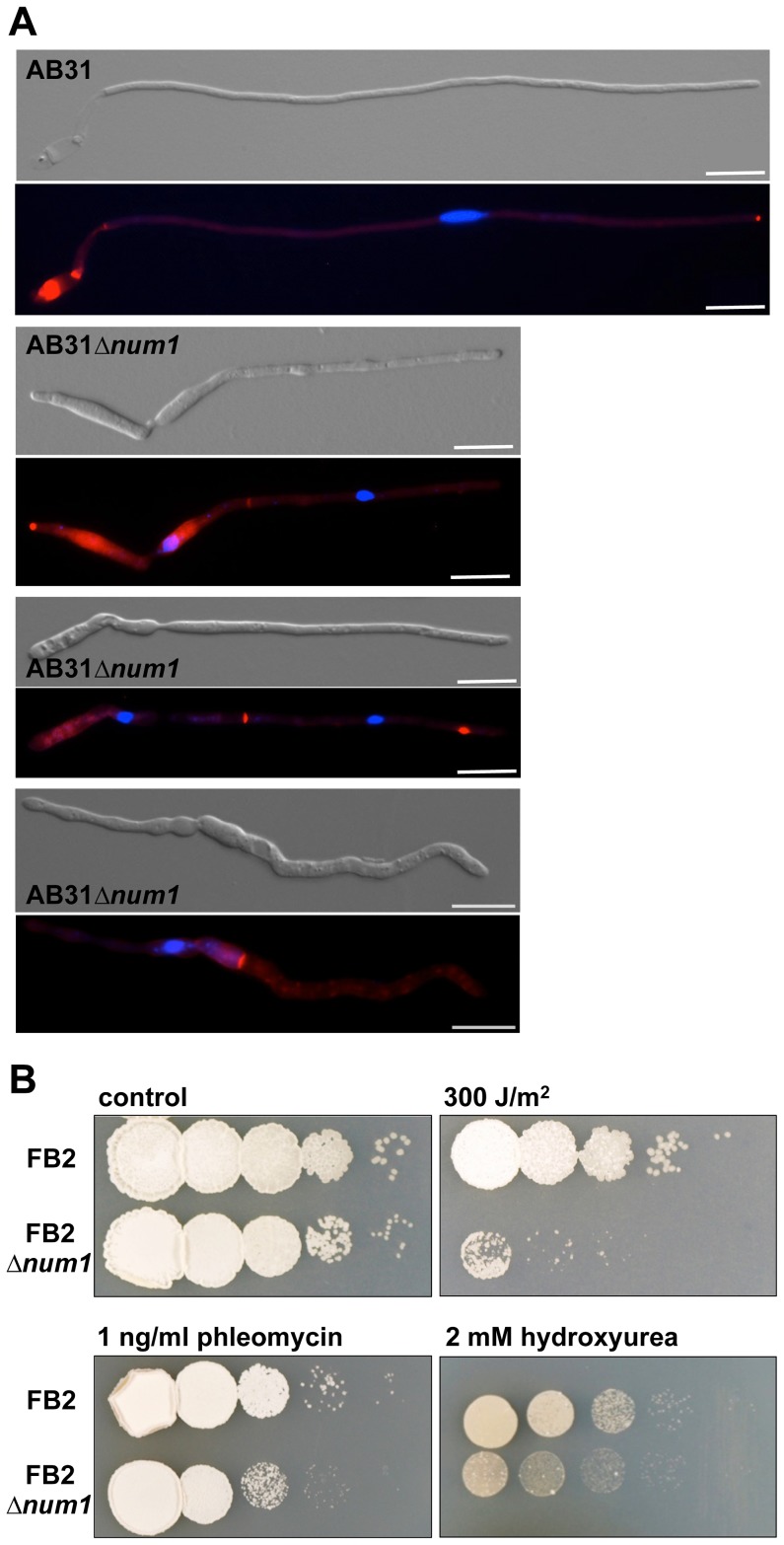
Num1 influences the cell cycle and cell division as well as the cellular response to DNA damage. (**A**) Induction of the bE1/bW2 heterodimer in wild-type cells (AB31) induces a G2 cell cycle arrest, resulting in hyphae with a single nucleus positioned in the tip compartment. In AB31*Δnum1*, 18% of the hyphae contain more than one nucleus, usually separated by a septum or a septum-like chitin structure, which is not always visible in the DIC-channel (see also Figure S6). In some cases, delocalized septa give rise to compartments that are devoid of any nuclei (bottom panel). Filaments were stained with DAPI and Congo Red, which effectively stains chitin in fungal cell walls [26], to visualize nuclei and septa within the same hypha. Scale bars: 10 µm. (**B**) Sensitivity of an FB2*Δnum1* deletion strain to DNA-damage in comparison to wild-type cells. Serial 10-fold dilutions were spotted on complete medium containing the indicated DNA-damage inducing supplements, or subsequently irradiated with 300 J/m^2^ UV light (254 nm), respectively. Pictures were taken 2–3 days after incubation at 28°C.

To define whether Num1 acts in line with previously known functions of the NTC-complex, we investigated cell cycle regulation and the response to DNA damage in AB31*Δnum1*. In AB31, the filaments generated after induction of the bE1/bW2 heterodimer are arrested in the G2 phase of the cell cycle, harboring a single haploid nucleus that is positioned in the tip compartment [39]. In filaments of AB31*Δnum1*, however, deviations from this pattern became evident. 18% of the filaments (N = 100) exhibited two (or more) nuclei within a single hyphal compartment; additionally, compartments devoid of any nuclei were generated, most likely due to the aberrant formation of septa (Figure 3A). In accordance with this observation, FACS analysis of AB31 cells revealed that upon prolonged induction of the bE1/bW2-heterodimer cells with a 2C DNA content accumulated, whereas in AB31*Δnum1*, an increased number of cells with both 1C and 2C DNA content was observed (Figure S9), indicative for a deregulated cell cycle. Taken together, our results implicate that the *num1* deletion affects both cell cycle regulation as well as cell division.

Consistent with the function of the NTC in response to DNA damage, we found that *Δnum1* cells exhibited increased sensitivity to UV irradiation and DNA-damaging agents. The survival rate upon UV-treatment was reduced approximately by a factor of 10 in AB31*Δnum1* (Figure 3B). Similarly, treatment with phleomycin, causing double-strand breaks in DNA [40] or hydroxyurea, which triggers stalling of the replication fork during DNA synthesis [41], led to reduced survival of *Δnum1* cells (Figure 3B).

### Num1 affects splicing of mRNA on a global scale

To assess whether the Δ*num1* mutation affects splicing, we employed next-generation RNA sequencing (RNA-Seq) to determine the transcriptome of strains AB31 and AB31*Δnum1*. *bE1/bW2* expression was induced for eight hours; at this time point, in both strains a comparable quantity of filamentous cells was observed. The expression levels of *bW*, of the central regulator *rbf1* (a direct *b*-target gene) and of *num1* were monitored by quantitative RT-PCR analysis to ensure similar expression levels (Figure S10). For both AB31 and AB31*Δnum1*, RNA-Seq experiments from three independent biological samples were performed, and sequences were compared to the manually curated *U. maydis* gene models of the MIPS *Ustilago maydis* Database (MUMDB). Splicing efficiency was calculated from the normalized read number within introns in relation to the normalized read number of exon regions of a gene (for details, see Materials and Methods). Based on 2142 intron sequences, introns of AB31 genes were spliced with an average efficiency of 0.93 (±0.13); splicing efficiency in AB31*Δnum1* was significantly lower (factor −1.44, t-test p = 6.22*10^−111^), with an average of 0.80 (±0.23). The splicing defect observed by RNA-Seq was verified for a small set of genes by qRT-PCR (Figure S11). In general, we noticed a wide variation in splicing efficiency of different introns in response to the *num1*-deletion. For 80.2% of the introns (1717 of 2142), the intron retention rate (fraction of RNA in intronic sequences in relation to exonic sequences) in AB31*Δnum1* was at least twice as high as in the wild-type strain, indicating that Num1 is required for splicing in general. We noticed, however, that for few introns (83, allowing a 10% threshold) splicing was not affected, while other introns were barely spliced at all in AB31*Δnum1*. Differences in intron retention rates were specific for discrete introns, and not for particular transcripts, as we observed that in genes with more than one intron separate introns were spliced to different extent (Table S1, examples are shown in Figure S12).

It has been proposed that the function of the NTC with regards to cell cycle regulation or DNA damage repair may result from the decreased splicing efficiency of intron-containing genes involved in recombination, repair, cell cycle or chromosome segregation [42]. We did not observe an enrichment of genes with high intron retention rates in any “category for functional annotation” (FunCat). However, inspection of genes in the FunCats “cell cycle” and “DNA repair” (Table S2) revealed that for individual genes involved in DNA repair splicing is impaired, as, for example, for *rad51* (*um03290*), for the gene for the repair exonuclease Rec1 (*um11016*), or for *mms2* (*um10097*), involved in the error-free post-replication repair. Similarly, splicing of genes involved in cell cycle control, as *cks1* (*um03210*), encoding a regulatory subunit of cyclin-dependent kinases, or the gene for the cyclin dependent kinase 1 (*cdk1*, *um 10705*) is affected in AB31*Δnum1* (Table S2, Figure S13).

We have shown before that cell cycle and hyphal development are controlled by a cascade of transcription factors [2]. Intriguingly, although most *U. maydis* genes contain no introns, both *bW* and *bE* carry single introns; *rbf1* harbors even four introns. It could be well possible that the observed phenotype of the *Δnum1* strains is caused by inefficiently spliced genes of the master regulators for filamentous growth and pathogenic development. In AB31, the *bW2*-allele is expressed without the intron sequence [24], but we observed that *bE1* is indeed spliced less efficiently in AB31Δ*num1* (Table S3). However, all direct b-target genes were induced to a similar extent in AB31*Δnum1* compared to AB31 (Table S4), which implies that expression of the *bE* and *bW* genes in the *Δnum1* background is sufficient to provide full functionality of the bE/bW heterodimer.

**Figure 4:**
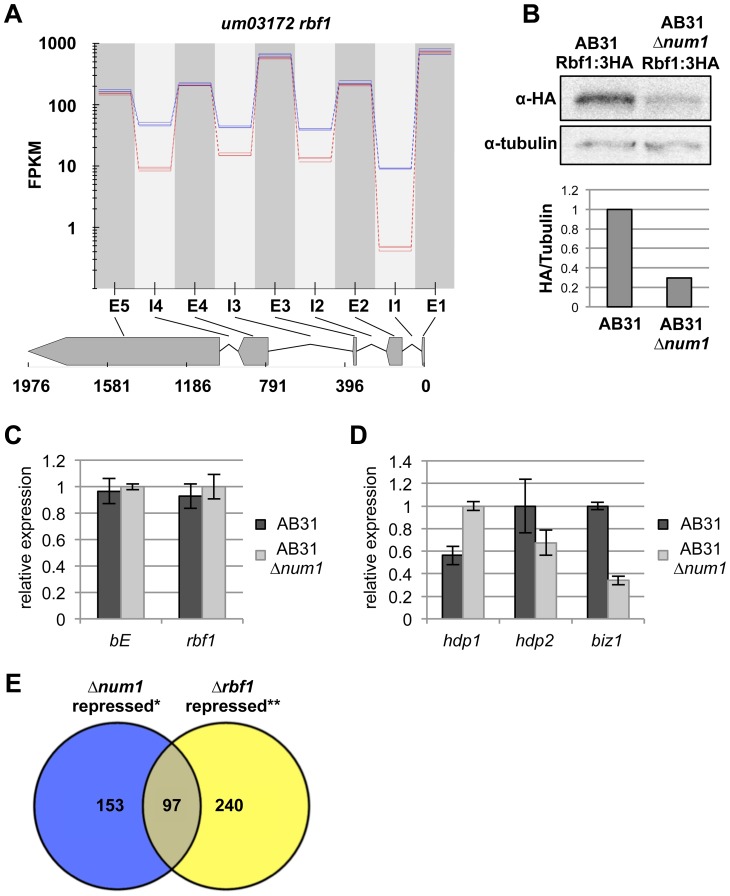
Reduced splicing efficiency of the *rbf1*-gene leads to impaired function of the Rbf1 master regulator. (**A**) Plot depicting splicing efficiency of the *rbf1*-gene. Plotted are the FPKM values (fragments per kilobase of sequence per million fragments mapped) across the genomic region indicated (coordinates in nucleotides) of three independent RNA-Seq experiments for AB31 wild-type (blue lines) and AB31*Δnum1* (red lines), respectively. Exons (E) and introns (I) are indicated. All four introns show increased intron retention rates in AB31*Δnum1*. (**B**) Western analysis showing abundance of Rbf1:3×HA and α-tubulin (loading control) from AB31 wild-type and Δ*num1*-deletion strains. In AB31*Δnum1*, Rbf1 is reduced to 30% of wild-type-level (Quantification: ImageJ [109]). (**C, D**) Gene expression analyses of *b*- and *rbf1*-genes (**C**) as well as *rbf1*-target genes (**D**) using qRT-PCR. RNA samples were isolated from strains AB31 and AB31*Δnum1* eight hours after induction of the bE1/bW2-heterodimer. Gene expression is shown relative to the highest expression value, using *actin* and *eIF2b* for normalization. Shown are the mean values of three biological and two technical replicates. Error bars represent the SD. (**E**) Venn diagram depicting the total number of genes repressed in AB31*Δnum1* and AB31*Δrbf1*. * RNA-Seq analysis, this study; ** Microarray analysis conducted five hours after induction of the bE1/bW2-heterodimer [2].

In accordance with the unaltered functionality of bE/bW, the expression of the direct target gene *rbf1*, was not altered in AB31*Δnum1* (Figure 4C); however, all four introns were spliced less efficiently. Under the assumption that splicing of the introns occurs independently from each other, only in 45% of the mature mRNA in AB31*Δnum1* all introns were spliced correctly, compared to 80% in AB31 wild-type cells (Figure 4A, Table S3). The splicing defect in *rbf1* was validated via RT-PCR and qRT-PCR analyses (Figure S14), showing additional unspliced variants and increased intron retention, respectively, in AB31*Δnum1*. Consistently, we observed that the abundance of the Rbf1 protein in AB31*Δnum1* is reduced to about 30% of wild-type level (Figure 4B), although qRT-PCR analysis showed that the total level of the *rbf1*-transcript was not altered (Figure 4C).

Rbf1 controls the expression of *biz1*, *hdp1* and *hdp2*, encoding transcription factors that are collectively involved in cell cycle regulation, filamentous growth and pathogenic development [43, Scherer, Vranes, Pothiratana and Kämper, unpublished]. In accordance with the reduced Rbf1 levels, RNA-Seq analysis revealed that *biz1* as well as *hdp2* expression was significantly down-regulated in AB31*Δnum1*, which was verified by qRT-PCR (Figures 4D and S15, Table S4). *hdp1* splicing efficiency was reduced in AB31*Δnum1* from 0.83 to 0.14 (Table S3); interestingly, *hdp1* RNA-levels were increased about 2-fold, which may argue for a self-regulation of the gene (Figures 4D and S15, Table S5). In total, we observed an at least two-fold down-regulation for 228 genes in AB31*Δnum1*; intriguingly, 89 of these genes overlap with a set of 351 genes that has been identified as down-regulated in response to a deletion of *rbf1* in AB31 (Figure 4E, Table S5, [2]).

As *rbf1* is one of the major regulators implicated in filamentous growth and cell cycle regulation, it was tempting to speculate that the reduced splicing efficiency of the gene may be the cause for the observed phenotype in *Δnum1*-mutants. To test this hypothesis, we exchanged the *rbf1* gene with an intron-free derivative in strains AB31 and AB31*Δnum1*. However, we observed that the intron-free copy of *rbf1* is not capable to substitute for the native gene; despite wild-type-like expression levels the strains did not grow filamentous, nor were the known *rbf1*-target genes induced (Figure S16). Obviously, introns are essential for the expression of the Rbf1-protein. As a consequence, expression of the *rbf1* cDNA did not rescue the *Δnum1* phenotype either.

In summary, we conclude that Num1 is a structural component of the conserved Prp19/CDC5L complex and fulfills common functions that have previously been associated with this complex, as cell cycle control, cellular response to DNA damage, and splicing. Our data indicate that the observed phenotype in *U. maydis* hyphae may be at least in part the result of the splicing defect of genes involved in cell cycle control, DNA damage repair and of central components of the regulatory cascade controlling filamentous growth and pathogenic development.

### Num1 interacts with the Kin1 motor protein

To further elaborate the molecular function of the Num1 protein, we employed the yeast two-hybrid system. Proteins identified as potential interactors for Num1 are shown in Table S6. Consistent with a putative function of Num1 within the Prp19/CDC5L complex, the CDC5L homologue Cef1 was isolated as an interacting protein.

Unexpectedly, we identified various interactions with proteins involved in cellular transport processes. Um10158 is an adaptin-like protein from clathrin/coatomer-adapter complexes (IPR002553) mediating endocytic protein transport between ER and Golgi. Um03539 and Um11510 contain a BAR-domain (IPR004148) and a BRO1-domain (IPR004328), respectively, and are thus likely involved in intracellular vesicular transport processes and protein targeting to endosomes or the vacuole. The conventional kinesin motor protein Kin1 [formerly named Kin2, 20] functions in long distance trafficking within fungal hyphae as it transports Dynein along the microtubule cytoskeleton towards their plus-ends directed to the hyphal tips [9], [14]. We were not able to generate deletion strains for *um10158*, indicating that the respective protein might be of essential function. Deletion analysis for *um03539* and *um11510* in strain AB31 revealed no obvious phenotype with respect to polar growth and vesicular movement (data not shown), indicating that the respective proteins have no function with respect to Num1-associated processes. For *kin1*, however, it had already been demonstrated that the gene has impact on hyphal morphology [14, see below].

**Figure 5:**
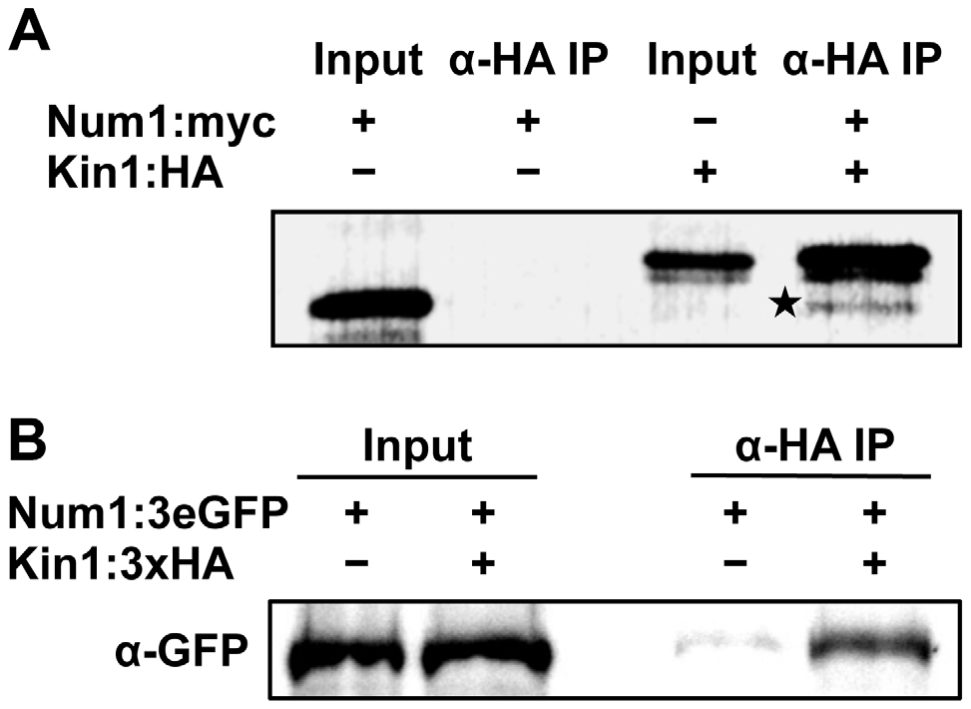
Num1 physically interacts with the Kin1 motor protein. (**A**) *In vitro* expression and co-immunoprecipitation of Myc-tagged Num1 and HA-tagged Kin1. Proteins were labeled with biotin during *in vitro* synthesis, detected with streptavidin-conjugated alkaline phosphatase, and visualized with NBT/BCIP. In the negative control (Num1:Myc), no unspecific binding of Myc-tagged Num1 to the HA-antibody was detected. In contrast, after co-incubation with HA-tagged Kin1, the Myc-tagged Num1 protein was co-immunoprecipitated (indicated by asterisk), demonstrating that the Num1 protein physically interacts with Kin1 *in vitro*. (**B**) *In vivo* co-immunoprecipitation of *U. maydis* strains expressing HA-tagged Kin1 and 3eGFP-tagged Num1 under control of their endogenous promoters. After crosslinking of proteins using DSP, α-HA coupled agarose beads were used to precipitate Kin1. The Num1 protein was detected on Western blots using an α-GFP antibody. As negative control, a strain expressing only Kin1:3×HA was used.

To confirm the interaction of Num1 with Kin1, co-immunoprecipitation analyses were carried out. A full-length Num1:Myc-tagged protein and a Kin1:HA-tagged protein fragment encompassing amino acids 650–968 were generated by coupled *in vitro* transcription/translation, and Num1:Myc was specifically co-precipitated from mixtures of Kin1:HA with an anti-HA antibody (Figure 5A). By use of the yeast two-hybrid system we mapped the interaction domain of Num1 (encompassing amino acids 67 to 147) with the C-terminal Kin1 domain used in the co-immunoprecipitation, which provides additional evidence for a specific interaction with the Kin1 motor protein (Figure S17). The interaction was further verified *in vivo*, using *U. maydis* strain UNK197 that harbors *num1:3eGFP* and *kin1:HA* fusion genes integrated into their native loci by homologous recombination, respectively. By use of dithiobis[succinimidyl]-propionate (DSP) to crosslink proteins in extracts prior to immunoprecipitation, the Num1:3eGFP protein was efficiently co-precipitated with the Kin1:HA protein using anti-HA antibodies (Figure 5B). Without cross-linking of the proteins, only a faint signal was detectable (not shown), suggesting that the interaction is only transient. Taken together, we conclude from our data that Num1 interacts with the motor protein Kin1.

### Num1 and the motor protein Kin1 are functionally connected

**Figure 6:**
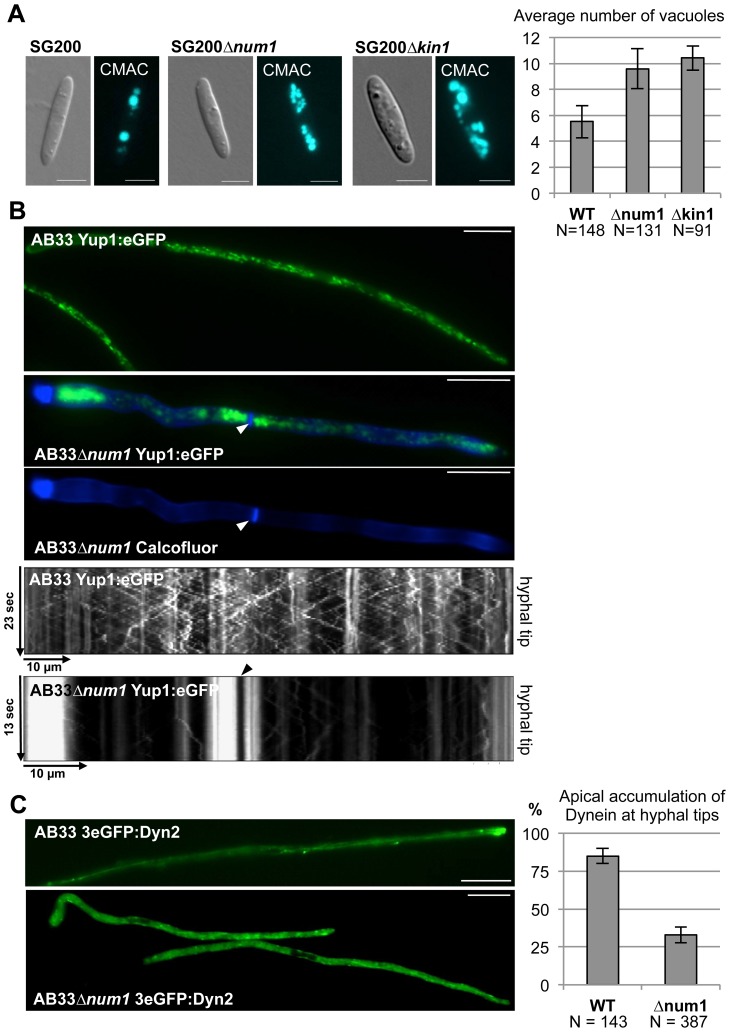
Num1 and Kin1 are functionally connected. (**A**) The *num1* deletion leads to aberrant vacuole morphology. Vacuoles were visualized by CellTracker Blue (7-amino-4-chloromethyl-coumarin, CMAC) staining. Both *num1* and *kin1* deletion strains contain more, but smaller vacuoles in comparison to wild-type sporidia. Scale bars: 5 µm. Right panel: Quantification of the average number of vacuoles of strains indicated. N represents the number of individual cells analyzed. Mean values of three independent experiments are shown. (**B**) The *num1* deletion leads to an aberrant distribution of early endosomes. For visualization of early endosomes, a Yup1:eGFP fusion protein [9] was expressed under control of the constitutively active *P_otef_*-promoter in strains AB33 and AB33*Δnum1*, which harbor *b*-genes under control of the nitrate-inducible *P_nar1_*-promoter. Endosomes were analyzed 14 hours after *bE1/bW2*-induction. Septa (arrowhead) were visualized by Calcofluor White staining. Yup1-labelled early endosomes accumulate at basal and apical parts of the hyphae as well as around delocalized septa in AB33*Δnum1* in contrast to wild-type hyphae. Scale bars: 10 µm. Movies detecting Yup1:eGFP-fluorescence of the depicted hyphae are shown in Videos S1 and S2. Shown below are the corresponding kymographs (time and distance as indicated) reflecting the motility of early endosomes. In AB33 hyphae, early endosomes travel continuously towards the hyphal tip (anterograde) and reverse direction to travel back towards the septum (retrograde). Pausing organelles are reflected by a vertical line. In contrast, endosomal motility is drastically reduced in AB33*Δnum1* hyphae containing additional septa, where the majority of the Yup1:eGFP-signal is non-motile. An arrowhead indicates the septum. (**C**) The localization of Dynein is dependent on Num1. In AB33 filaments 3eGFP:Dyn2 fusion proteins form comet-like motile structures that accumulate at the hyphal tip and from there move into retrograde direction [9]. In filaments of AB33*Δnum1* strains the apical accumulation is significantly reduced (p = 0.002); instead a stronger signal is observed in the cytoplasm. Dynein localization was monitored 14 hours after induction of the bE1/bW2 heterodimer. Scale bars: 10 µm. Right panel: Quantification of the apical accumulation of 3eGFP:Dyn2 fusion proteins. N represents the number of hyphae analyzed. Mean values of three independent experiments are shown.

In addition to the physical interaction, several lines of evidence argue for a functional connection between Num1 and Kin1. Induced expression of *bE1/bW2* in a *Δkin1* strain leads to short curved hyphae with delocalized septa and the formation of bipolar filaments [14]. Similarly, the induction of hyphal growth in AB31*Δnum1* leads to irregular or bipolar filaments with an aberrant insertion of septa (Figure 1, Table 1). In strains deleted for *kin1* the number of vacuoles is increased, while size is reduced [13]. We observed a similar phenotype in strains SG200*Δnum1* (Figure 6A).

To analyze the functional relation between the two proteins further, we generated strains deleted for both genes. Filaments induced in AB31*Δnum1/Δkin1* displayed a more severe phenotype with respect to polarized growth, as hyphae exhibited a very irregular, swollen morphology, and also the number of branched hyphae and hyphae with an altered septation pattern was significantly higher than in AB31*Δnum1* or AB31*Δkin1* (Figure S18). Infection experiments with SG200*Δnum1*, SG200*Δkin1* and SG200*Δnum1/Δkin1* deletion strains revealed that the virulence of the double deletion strain is significantly reduced when compared to the respective single deletion strains (Figure S18). The more severe phenotype excludes that both genes are in pure epistatic interaction; besides their function within a common complex, both proteins must fulfill functions that are independent from each other.

### Num1 and Kin1 are both required for trafficking of early endosomes

The deletion of *kin1* results in a loss of rapid bi-directional trafficking of early endosomes. Early endosomes visualized by a GFP-fusion with the endosomal t-SNARE Yup1 (Yup1:eGFP) [4] accumulate at the hyphal tips of *kin1*-deletion mutants [9], while in the wild-type situation Yup1:eGFP labeled vesicles shuttle bi-directionally throughout the hyphae along microtubule tracks [4]. The anterograde transport of early endosomes is mediated via the motor protein Kin3 (directed to the microtubule plus-ends at the hyphal tip), whereas the retrograde transport is accomplished by Dynein1/2 [8], [10], . Since the Kin1 motor protein transports Dynein toward the hyphal tips, the accumulation of early endosomes at the tip of Δ*kin1* hyphae can be explained indirectly by the failure of the Kin1-dependent anterograde transport of Dynein [9].

The deletion of *num1* leads to a comparable scenario in the distribution of early endosomes, which appeared to cluster at hyphal tips and the distal pole of the hyphae as well as around the delocalized septa frequently found in *Δnum1*-hyphae (Figure 6B). Endosomal motility was monitored in strain AB33, in which the *b*-genes are expressed under control of the nitrogen-responsive *P_nar1_*-promoter [44]. Filamentous growth in AB33 is repressed by ammonium and can be elicited in nitrate-containing media. In AB33*Δnum1* filaments, long-distance movement of early endosomes was constrained, and velocity of the vesicles was reduced to a speed of 1.14 µm/sec in AB33*Δnum1*, in contrast to 1.46 µm/sec in wild-type hyphae (t-test p = 0.005). In AB33*Δnum1* filaments with aberrant septa, long-distance trafficking of endosomes was entirely abolished, and only residual movement of vesicles was observed (Table 2, Videos S1 and S2). Next we addressed the question whether the altered movement and positioning of early endosomes in *Δnum1* strains may be caused by a distortion of cytoskeletal elements. Neither the arrangement or abundance of microtubules (visualized by an α-tubulin mCherry fusion, Tub1:mCherry) nor of actin patches (visualized by LifeAct:YFP [45]) was altered in the *Δnum1* mutant strain (Figure S19). To analyze the orientation of the microtubule cytoskeleton, we used strains expressing an α-tubulin GFP fusion [14] together with the microtubule plus-end marker Peb1, fused to RFP, which localizes to growing microtubule plus-ends [46]. As previously described [46], in wild-type interphase sporidia the majority of microtubule plus-ends is oriented towards the budding cell and the opposing cell pole of the mother cell; in *Δnum1* sporidia, the orientation of the microtubule cytoskeleton was not considerably altered (Figure S20 A,B). Similarly, in hyphae the majority of microtubule plus-ends is oriented towards the cell poles, i.e. hyphal tips and basal retraction septa [9]. In *Δnum1*-mutants, we observed microtubule plus-ends also in close vicinity to the delocalized septa (Figure S20 C–E). Apparently, endosomes accumulate at microtubule plus-ends in *num1*-deletion mutants, supporting the Kin1-dependent effect on endosomal transport. In some hyphae of the *num1*-deletion strain we observed the appearance of mitotic spindles, which supports the defect in cell cycle regulation.

**Table 2:** Reduced endosomal motility in *num1*-deletion strains.

	AB33Δ*num1* (hyphae with delocalized septum)	AB33Δ*num1* (hyphae without septum)	AB33
endosomal motility over a distance >5 µm	13% N = 150	42% N = 150	92% N = 150
Ø velocity of motile endosomes	n.d.	1.14±0.25 µm/s N = 20 *	1.46±0.42 µm/s N = 20 *

* t-test p = 0.005.

To determine whether the effect on endosomal trafficking in AB33*Δnum1* strains is attributed to an aberrant anterograde transport of Dynein towards the hyphal apex, as it was suggested for *Δkin1* mutants, we visualized the subcellular distribution of Dynein using an N-terminal 3eGFP:Dyn2 fusion protein [9]. In wild-type filaments, 3eGFP:Dyn2 fusion proteins form comet-like motile structures that accumulate at microtubule plus-ends in hyphal tips, where they reverse direction and move into retrograde direction [9]. In AB33*Δnum1* filaments, however, the number of cells harboring an apical accumulation of 3eGFP:Dyn2 was significantly reduced (t-test p = 0.002); concomitantly, in most cells the 3eGFP:Dyn2 signal in the cytoplasm was higher than in wild-type filaments (Figure 6C).

As Kin1 affects motility of early endosomes only indirectly (via the anterograde transport of Dynein), we set out to analyze the movement of a direct Kin1-cargo. The myosin chitin synthase Mcs1 [15] localizes to Kin1-dependently transported vesicles [17]. It has been described before that 3eGFP:Mcs1 fusion proteins localize to the poles of growing sporidia, where it is secreted and participates in the synthesis of chitin [15]. *Δnum1*-mutants showed reduced Mcs1-accumulations at the growth region in budding cells, which coincides with the distribution of Mcs1 in *kin1*-deletion mutants [17] (Figure S21 A,B). In AB33 filaments, 3eGFP:Mcs1 localizes to distinct foci close to the cell membrane and forms a gradient towards the growth zone within the hyphal apex or localizes to the basal retraction septum, i.e. to sites of chitin synthesis. In contrast, in AB33*Δnum1* hyphae no tip-directed gradient was obvious and less foci were observed at the cell membrane (Figure S21 C,D), indicating an impaired delivery of Mcs1-containing vesicles. In many cases, Mcs1 was not observed at delocalized septa in *Δnum1*-mutant hyphae. In summary, *U. maydis* strains deleted for either *num1* or *kin1* show similar phenotypes with respect to hyphal morphology and septation, formation of vacuoles, endosomal trafficking as well as the localization of the Kin1-cargos Dynein and Mcs1, which strongly corroborates a functional interrelation of Num1 and Kin1.

### Num1 is required for transport of cytoplasmic mRNA particles

Recently, it has been shown that the RNA-binding protein Rrm4 is associated with early endosomes [7]. Rrm4 is required for cytoplasmic transport of mRNAs in *U. maydis* filaments [47]. We deleted *num1* in an AB33 strain expressing an Rrm4:eGFP fusion protein. In the wild-type background vesicles associated with eGFP-labeled Rrm4 shuttled within the hyphae to the distal and proximal ends. In contrast, movement in *Δnum1* was reduced, and similar to Yup1-labeled early endosomes, Rrm4-containing particles frequently accumulated in clusters at hyphal tips or around delocalized septa (Figure S22, Videos S3, S4), indicating that *num1* is required for cytoplasmic mRNA transport.

### Num1 has a functional homologue in the filamentous ascomycete *Aspergillus nidulans*

**Figure 7:**
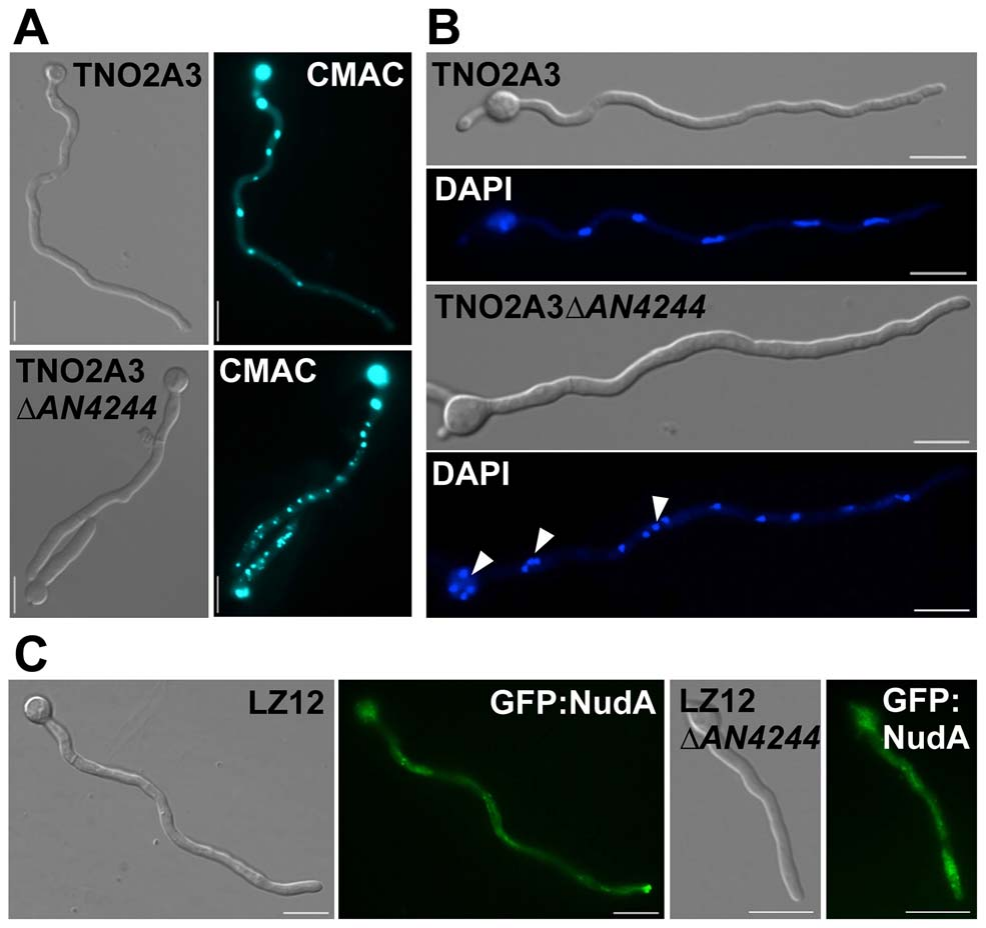
Deletion of the gene for a Num1-homologue in the ascomycete *Aspergillus nidulans* leads to a phenotype reminiscent to the *Δnum1* phenotype in *U. maydis*. (**A**) The morphology of vacuoles is affected by the *AN4244*-deletion. Spore suspensions of a wild-type (TNO2A3) and the *AN4244* deletion mutant were incubated for 18 hours at 37°C and the germinated filaments were CellTracker Blue (CMAC)-stained to visualize the vacuoles. Similar to the *num1*-deletion in *U. maydis*, the *AN4244*-deletion in *A. nidulans* leads to an increased number of vacuoles. (**B**) Deletion of *AN4244* affects nuclear positioning. Spore suspensions of *A. nidulans* wild-type (TNO2A3) and the *AN4244* deletion mutant were incubated for 12 and 18 hours, respectively, at 37°C, and the germinated filaments were DAPI-stained to visualize nuclei. In wild-type filaments, nuclei are evenly spaced, whereas in the mutant nuclei are often found in clusters. (**C**) The localization of Dynein (NudA) is dependent on AN4244. Spore suspensions of strains LZ12, expressing a GFP:NudA fusion protein, and LZ12*ΔAN4244* were incubated for 18 hours at 37°C. In LZ12 the GFP:NudA signal strongly accumulated in tips of germinating hyphae, whereas in mutant filaments, this apical accumulation was reduced; instead a stronger signal was observed in the cytoplasm. Scale bars: 10 µm.

To address the function of Num1 in filamentous fungi in general, we deleted a gene encoding a protein with high similarities to Num1 (AN4244, 49% similarity, 30% identity) in the ascomycete *Aspergillus nidulans* (strain TN02A3). The deletion mutants displayed smaller colonies with a reduced rate of conidiospore formation. Germination of spores was drastically reduced in the *ΔAN4244* mutant strains: after eight hours of incubation at 37°C, only 5% of the spores germinated, compared to 98% in the wild-type strain. Similar to the phenotype observed in *U. maydis*, hyphae from germinating *ΔAN4244* conidia were shorter and thicker than those of the wild-type strain. Staining with CellTracker Blue revealed that in the mutant the number of vacuoles was increased, while size was reduced (Figure 7A). In contrast to *U. maydis, A. nidulans* hyphae have cell compartments with multiple nuclei. In wild-type hyphae, these nuclei are evenly distributed. In *ΔAN4244*, however, nuclei were often found in clusters (Figure 7B). A similar phenotype has been observed earlier for strains deleted for KinA, the homologue of the *U. maydis* Kin1 [48]. To test a potential functional relation between AN4244 and KinA in *A. nidulans*, we analyzed, in analogy to *U. maydis*, the distribution of Dynein (NudA in *A. nidulans*). We deleted *AN4244* in strain LZ12, which harbors a GFP:NudA fusion protein. Similar as described for the *U. maydis* hyphae, GFP:NudA was predominantly localized at the tip of hyphae in strain LZ12, while the apical accumulation was reduced in LZ12*ΔAN4244* (Figure 7C). Taken together, our data suggest that the *A. nidulans* AN4244 has a conserved function as the *U. maydis* protein with respect to polarized growth and intracellular transport processes.

## Discussion

In this study we have characterized the function of the Num1 protein in *U. maydis*. We show that Num1 is a functional homologue to the human SPF27 protein, which is an integral component of the conserved spliceosome-associated Prp19/CDC5L-complex (NTC). Deletion of the *num1* gene affects polar and filamentous hyphal growth, cell cycle control as well as DNA repair. Consistent with its function as component of the NTC, deletion of *num1* results in a genome-wide reduced splicing-activity, which could explain the observed pleiotropic phenotype. In addition we demonstrate a previously unknown function of an NTC-component in the cytoplasm, where Num1 interacts with the Kinesin 1 motor protein. This functional interconnection of Num1 with elements of the cytoskeleton implies additional, yet unknown functions during intracellular trafficking processes.

### Num1 is a conserved component of the spliceosome-associated Prp19/CDC5L-complex (NTC)

Num1 shows significant similarities to SPF27, and its interaction with Prp19 and Cef1 is in accordance with the interactions within the NTC complex demonstrated in *S. pombe* [28], humans [21] and plants [49].

The NTC was initially identified in *S. cerevisiae* as a complex containing Prp19 and at least seven other components [50], most of which are conserved from yeast to human. The NTC is a non-snRNP (small nuclear ribonucleoprotein particle) multi-protein complex, and as a subunit of the spliceosome it is required for intron removal during pre-mRNA splicing. The complex associates with the spliceosome during or after dissociation of U4 snRNP and participates in the regulation of conformational changes of core spliceosomal components, stabilizing RNA-RNA- as well as RNA-protein interactions [reviewed in 51]. The only known enzymatic function of the NTC is a conserved U-box domain with E3-ubiquitin ligase activity of the Prp19-protein [52], [53]. It was conclusively shown that individual core components of the NTC, as Prp19, Cef1/Cdc5 in *S. cerevisiae* and *S. pombe*, or the *S. pombe* SPF27 homologue Cwf7 independently affect splicing [42], [50], [54]–[59]. However, it is mostly unresolved to which extend single components of the NTC influence splicing of individual introns. We now show that one of the core components of the NTC, the SPF27 homologue Num1, has a global effect on splicing in *U. maydis*, as retention rates of nearly 80% of all introns were significantly increased in *num1*-deletion mutants. We observed variations in intron retention between different transcripts in response to the *Δnum1* mutation. Similar observations have been made in *S. cerevisiae* on a genome-wide scale for depletion of individual components of the spliceosome, including the NTC-components Prp19 and Prp17. The resulting splicing defects displayed considerable differences in different mRNAs, indicating that individual splicing factors affect a discrete set of genes [60]. It is well possible that structural alterations of the spliceosome complex after deletion of *num1* result in altered recognition/affinity of specific introns, probably caused by their different GC content or secondary structures. Following the conclusions made in *S. cerevisiae*, we favor the possibility that deletion of *num1* leads to an altered integrity of the NTC, resulting in a reduced activity and/or specificity and hence a diminished splicing efficiency. However, we cannot rule out that Num1 may recruit splicing enhancer- or inhibitor-proteins that might affect splicing of only a subset of introns.

### Splicing defects can account for pleiotropic phenotypes in *num1*-deletion mutants

In addition to disturbed polarity we frequently observed delocalized septa in *num1*-deletion strains; in addition, chitin-staining indicate structural differences to wild-type septa. Formation of septa requires a highly coordinated and dynamic positioning of septins into ring-like structures [61], a process tightly regulated by the small GTPase Cdc42, its guanine exchange factor Don1 and the protein kinase Don3 [62]–[64] as well as by formins or Cdc4, an essential light chain of the type II myosin motor protein [65]. The genes encoding the septins Cdc3 (*um10503*) and Sep3 (*um03449*), but also Cdc4 (*um11848*) display significantly increased intron retention rates (Table S1) and the expression of *don3* (*um05543*) is reduced more than 2-fold in the *num1*-deletion strain (Table S4), which altogether might rationalize the formation of delocalized septa.

We found that Num1 affects splicing of several genes of the functional class “cell cycle”, including genes for the catalytic and regulatory subunits of cyclin-dependent kinases, for mitotic cyclins as well as for γ-Tubulin (Table S2). Similarly, several genes grouped in the functional class “DNA-repair” (e.g. for the DNA repair recombinase Rad51, the nucleotide excision machinery component Rad1 and the repair exonuclease Rec1) show increased intron retention rates (Table S2). The synergistic effect of several genes with reduced splicing efficiency (and the resulting reduced activity) within the same functional category may be sufficient to explain the effects on the corresponding cellular processes. However, various components of the NTC have been found to be directly involved in DNA damage repair. Initially, the resistance to the interstrand cross-linking reagent psoralen in a *S. cerevisiae* Prp19 mutant strain [32] has been attributed to the splicing defect of the intron containing *RAD14* gene [66]. Subsequently, it has been conclusively shown that the DNA damage phenotype is independent from the splicing defect [42]. In addition, for Prp19 several interaction partners have been identified which belong into the functional classes “cell cycle”, “chromatin structure” and “DNA repair” rather than pre-mRNA processing. For example, the human Prp19/Pso4 directly interacts with terminal deoxynucleotidyl-transferase (TdT), which is involved in DSB repair [67]. The human Cef1-homologue CDC5L interacts with the Werner syndrome protein WRN, a DNA helicase that functions during homologous recombination in response to DNA-damage [35] and also with the non-homologous end-joining (NHEJ) factor DNA PKcs [68].

Thus, it is well possible that also in *U. maydis* the increased UV-sensitivity of *Δnum1* mutants results from a direct function of Num1 in a DNA damage repair pathway. Similarly, disturbed cell cycle and cell division can be explained as indirect effect of a disturbed DNA-damage repair. Interaction of the human CDC5L with the cell cycle checkpoint kinase ATR (“ataxia-telangienctasia and Rad3-related”) was shown to be required for activation of the ATR kinase as well as for further downstream effectors and mediators of ATR function as the checkpoint kinase CHK1 [69]. In *U. maydis*, the homologues of ATR1 and CHK1 are known to be critical cell cycle regulators in addition to their decisive roles in the DNA-damage response [70], [71].

We have shown previously that cell cycle and polarized growth in *U. maydis* is regulated by a cascade of transcription factors controlled by the bE/bW-heterodimer [2]. Several of the transcription factors of this b-cascade were found to have increased intron retention rates or a differential expression pattern in AB31*Δnum1* and might collectively contribute to the effects on cell cycle control and polar hyphal growth. For example, splicing efficiency of the *hdp1* gene, which encodes a homeodomain transcription factor required for filamentous growth and cell cycle control (Pothiratana and Kämper, unpublished data), was reduced from 83% in wild-type to 14% in the *num1*-deletion strain. More importantly, all four introns of *rbf1* are spliced less efficiently in the *num1*-deletion strain. As a consequence, protein levels of Rbf1, the master regulator for hyphal development, are significantly decreased in *Δnum1* strains. In accordance with the reduced Rbf1 protein levels in *Δnum1* strains, we observed the down-regulation of *rbf1*-dependently expressed genes. Among these are the *rbf1*-dependently expressed genes encoding the transcription factors Biz1 and Hdp2, which were previously described as cell cycle regulators and factors for filamentous growth and pathogenic development [43; Scherer and Kämper, unpublished data]. Conclusively, we can assume that the reduced splicing efficiency of the *rbf1* gene and in turn the deregulation of *rbf1*-dependently expressed genes contributes to hyphal morphology and the deregulated cell cycle in *num1*-deletion mutants. A direct proof of this hypothesis by replacing the *rbf1* open reading frame with a cDNA copy failed. The intron-free copy, although expressed to the same level as the intron-containing copy, was not able to provide wild-type Rbf1 function, even when introduced into wild-type cells. Such a reduced expression of cDNA copies in relation to intron-containing genes has been described frequently before, and in various cases this has been related to direct effects of introns to translational efficiency [reviewed in 72], [73].

### Num1 has a specific role in cytoplasmic trafficking processes

Aberrant or incomplete splicing events of the genes encoding motor proteins by themselves would be an explanation for the impaired endosomal trafficking processes observed in *num1*-deletion strains. However, none of the genes for the major transport-mediating motor proteins (*kin1*, *kin3*, *dyn1/2*) contains introns or show altered expression levels in the *Δnum1*-mutant. Although we cannot rule out an effect caused by aberrant splicing of genes for unknown components of the transport machinery, we favor a specific role of the Num1 protein in intracellular trafficking processes, based on the observation that (a) Num1 is localized in distinct foci in the cytoplasm, implicating functions beyond its role as a splicing factor, and (b) Num1 interacts with the Kin1 motor protein, which is involved in long-distant transport processes during filamentous growth [9], [12], [14]. The physical interaction of Kin1 and Num1 is in line with the similar phenotypes of the two mutant strains with respect to cell morphology, determination of polarity and septation, and morphology of vacuoles. *Δnum1* and likewise *Δkin1* hyphae display impaired trafficking of early endosomes and reduced apical localization of Dynein, two interconnected phenotypes, as the transport of early endosomes relies on Dynein and the anterior transport of Dynein depends on Kinesin 1. In addition, the distribution of the direct Kin1 cargo Mcs1 is altered in both Δ*kin1* and Δ*num1* mutants, which argues for a direct connection of Kin1 and Num1. However, we can rule out that Num1 is required for all Kin1-related processes, as (a) both mutations appear not to be epistatic, and (b) the *num1* and *kin1* mutant strains have overlapping, but not identical phenotypes; for example, haploid *Δkin1* strains are defective in mating [20], while *Δnum1* strains are not. This is anticipated, as each of the proteins participates in different cellular processes. The conventional kinesin is best known for its function in cellular transport processes. However, conventional kinesin has also been implicated in cell signaling, or to serve as *bona fide*, static anchor for mRNA–protein complexes or vesicular compartments, or to participate in the phosphorylation of cargo proteins to which it binds [reviewed in 74]. As outlined above, also SPF27 (or the NTC) participates in a wide spectrum of processes that, however, show no overlap with Kin1-dependent processes.

Interaction of SPF27 homologues with conventional kinesin motor proteins is not specific to *U. maydis*, but appears to be conserved between basidio- and ascomycetes, as in *Aspergillus nidulans* the phenotype of deletion mutants of the SPF27 homologue AN4244 or the Kin1 homologue KinA [48] are comparable to those observed for *U. maydis*.

The association of a conventional kinesin motor protein with a component of the spliceosome-associated NTC has not been described so far and implies a novel function for SPF27-homologous proteins. The question is whether the Num1 protein fulfills dual functions, (a) during splicing as an NTC-component in the nucleus and (b) during Kin1-dependent intracellular trafficking processes in the cytoplasm, or whether these two disparate mechanisms are connected by one single function. Discrete cytoplasmic functions may serve to regulate the activity of Kin1, as described in neuronal development for the interaction of Kinesin-1 and JIP3, an interactor of the c-Jun N-terminal kinase [75]. Alternatively, Num1 could function as a scaffold or adapter molecule, providing stability to the motor protein/cargo-complex and/or to the NTC. In both scenarios, deletion of *num1* could alter the specificity/activity of Kin1-mediated transport processes, resulting in reduced motility of early endosomes and by that influence polar growth. We have not been able to visualize Num1 proteins moving or co-migrating with Kin1 along microtubule tracks. As GFP-Kin1 shows a strong cytoplasmic background, visualization of Kin1 molecules moving on microtubules has not been achieved yet [14], [76]. Taken into account that the interaction of Kin1 and Num1 appears to be transient, we cannot rule out that Kin1 and Num1 may interact on microtubule tracks.

A possible process that could couple both nuclear and cytoplasmic functions of Num1 is the cytoplasmic transport of mRNAs. In *U. maydis*, the RNA binding protein Rrm4 was found to be instrumental for bidirectional transport of mRNAs in filaments [47]. Intriguingly, cytoplasmic transport of Rrm4 is dependent on Kin1, Kin3 and Dynein, and recently it was shown that Rrm4 co-localizes with early endosomes [7], [77]. There is a remarkable overlap of the phenotypes of *rrm4*-, *num1*- as well as *kin1*-deletion mutants with respect to polarized growth [14], [78], which raises the possibility of a concerted function of all three proteins within the same process. Indeed, the motility of early endosomes associated with Rrm4:eGFP fusion proteins is drastically reduced in Δ*kin1*-mutants [7], [14] and, as we show here, also in *num1*-deletion strains.

In *Drosophila* oocytes, it has been shown that components of the exon junction complex (EJC) are essential for transport of *oskar* transcripts [79]. The EJC marks exon/exon-transitions during splicing, remains bound to the processed mRNA and serves as a quality control mechanism for the splicing reaction during translation [reviewed in 80]. Reminiscent to mRNA transport in *U. maydis*, transport of *oskar*-mRNA is also dependent on the conventional Kinesin 1 [81].

Unlike the components of the EJC that remain bound to mRNA during nuclear export, the NTC is thought to disassemble after the splicing reaction. However, recently it has been shown in *S. cerevisiae* that the NTC is required for efficient recruitment of TREX to the transcriptional machinery. This is mediated by the C-terminus of the NTC-component Syf1p, which connects TREX to Rpb1, the largest subunit of RNA-polymerase II [82]. TREX is a conserved protein complex (“transcription export”), which facilitates both transcription elongation as well as mRNA-export processes [83]. Yra1p, a component of the TREX complex, is an RNA-binding protein and interacts with the Mex67/Mtr2-receptor complex, which in turn act together with components of the nuclear pore complex (NPC) to guide the transcripts into the cytoplasm [84]–[86]. Thus, it appears well possible that NTC components remain tethered to the spliced mRNA. In this context it is interesting to note the recently discovered association of Kinesin 1- and Dynein to fungal nuclear pores, which mediate NPC-motility and are thus required for chromosome organization and nucleo-cytoplasmic transport [76]. This result corroborates a functional coupling between the nuclear transcription machinery and cytoplasmic transport processes in *U. maydis*.

**Figure 8:**
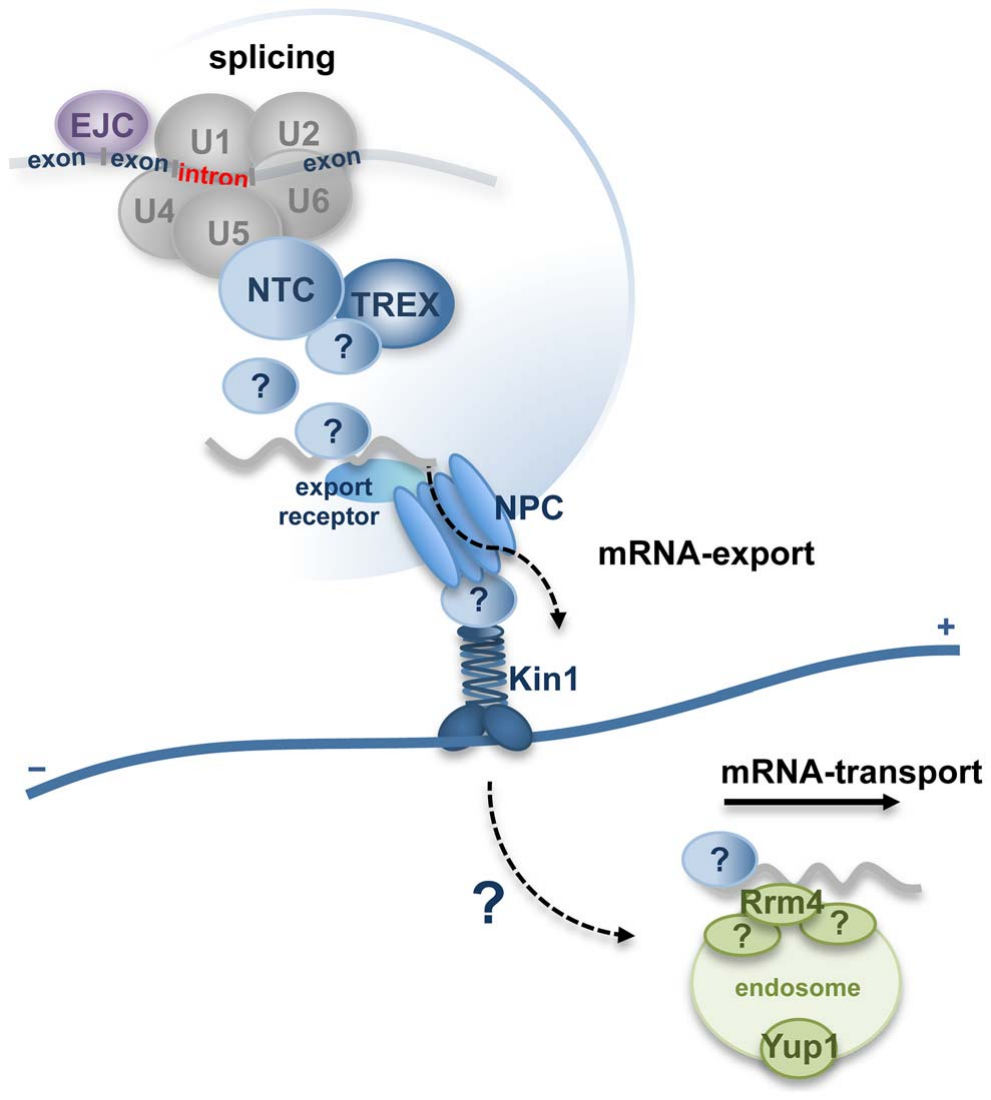
Hypothetical model for function of Num1 coordinating pre-mRNA splicing with nuclear pore complex-dependent export of mRNP-particles and microtubule-based mRNA-transport. Num1 is a component of the spliceosome-associated Prp19/CDC5L-complex (NTC) and has a global effect on splicing efficiency. NTC-components interact with the TREX (“transcription-coupled export”)-complex [82], which mediates the export of mature transcripts into the cytoplasm via the nuclear pore complex (NPC) [83]–[86]. Adapter proteins connect the mRNA to NPC-bound export receptors. The Kinesin-1 motor protein has been shown to be associated to the NPC and promotes nucleo-cytoplasmic transport processes [76]. mRNAs within the hyphae are distributed via the RNA-binding protein Rrm4, which shuttles associated with early endosomes along the polar microtubule cytoskeleton [7], [77]. Motility of the Rrm4 associated early endosomes depends both on Kin1 [7], [14] and Num1. Proteins labeled with (?) depict yet unidentified adapter-proteins that might function as connectors between the nuclear splicing machinery and the cytoplasmic NPC-bound Kin1. As Num1 is a component of the NTC complex and interacts with Kin1, it might fulfill a function during mRNA export processes, possibly by connecting exported mRNP to the cytoplasmic transport machinery via its interaction with Kin1.

The major finding of our study is that a component of the conserved spliceosome-associated NTC is involved in cytoplasmic trafficking processes. Due to the interaction of Num1 with the Kin1 and the functional connection to the RNA-binding protein Rrm4, it is tempting to speculate that Num1 could have a function during intracellular mRNA-transport processes. We hypothesize that one of the functions of the Num1-protein is to participate in the coordination of pre-mRNA splicing and the formation and export of mRNP-particles out of the nucleus. The transcripts would be transferred into the cytoplasm where they could be passed on to the microtubule-based transport machinery via the interaction of Num1 with Kin1 motor proteins that were found to be associated with the nuclear pore complex (Figure 8). In this model, additional adapter-proteins that act as connecting proteins between the nuclear splicing machinery and the cytoplasmic NPC-bound motor proteins cannot be excluded and remain to be identified.

## Materials and Methods

### Strains and growth conditions

*Escherichia coli* strain TOP10 (Invitrogen) was used for cloning purposes. Growth conditions and media for the cultivation of *E. coli* followed the protocols described previously [87]. The *Saccharomyces cerevisiae* strain AH109 (Clontech) was used for yeast two-hybrid interaction studies. S. cerevisiae cells were grown in YPDA complete medium, or on minimal medium (SD) supplemented with the dropout-mix needed for selection, as described in the Clontech Matchmaker™ GAL4 Two-Hybrid System 3 Manual (http://www.clontech.com). *Ustilago maydis* cells were grown in YEPSL [88], CM (complete medium) supplemented with 1% glucose (CM-G) and 1% arabinose (CM-A), respectively, or NM (nitrate minimal medium) [89] at 28°C. Solid media contained 2% agar. The induction of hyphal growth in AB31- and AB33 derivatives was done as previously described in [24]. Plate mating assays followed the protocol of [24]. Mating assays in liquid culture for the generation of dikaryotic hyphae were carried out as described in [90]. Pheromone stimulation of *U. maydis* cells was performed according to the protocol of [91]. *Aspergillus nidulans* was grown in CM (complex medium) [92] or MM (minimal medium) [93].

All *U. maydis* and *A. nidulans* strains used in this study are listed in Tables S7 and S8, respectively.

Plant infections were performed as described in [94]. For infection studies with *U. maydis*, the maize cultivar Early Golden Bantam was used under controlled conditions in a CLF Plant Climatics GroBank with a 16 h (28°C)/8 h (22°C) day/night rhythm. Disease symptoms were evaluated according to the disease rating criteria published previously [23].

### Yeast two-hybrid analyses

Screening for Num1-interacting proteins was performed as previously described [95], using the Matchmaker III system (Clontech). Plasmid pGBKT7-Num1 was generated by PCR amplification of the *num1* open reading frame (ORF) from genomic DNA (strain 521 [23]), introducing two incompatible *Sfi*I restriction sites at the 3′ and 5′ ends and subsequent ligation into the respective sites of pBGKT7 (Clontech). Plasmids pGAD-Prp19 and pGAD-Cef1 containing full-length as well as N- and C-terminal variants were generated by PCR amplification using either genomic DNA (for *cef1*) or a full-length cDNA library (for *prp19*) and subsequent ligation into pGAD-DS (Dualsystems Biotech) via *Sfi*I sites. Oligonucleotide sequences are given in Table S9.

### DNA and RNA procedures

Molecular techniques followed the protocols described in [87]. DNA from *U. maydis* was isolated according to the protocol given in [96]. Transformation procedures were performed as described in [97]. DNA from *A. nidulans* was isolated according to [98] and transformations were carried out as described in [99].

For gene deletions in *U. maydis*, a PCR-based approach [100] was used. Open reading frames were replaced by either hygromycin, carboxin or nourseothricin resistance cassettes. Similarly, for gene deletions in *A. nidulans*, a pyridoxine auxotrophic marker was used. C-terminal eGFP, RFP or 3×HA gene fusion constructs were generated in *U. maydis* via homologous recombination following the protocol given in [101], using *Sfi*I cassettes from plasmids pUMA647 (eGFP) [7], pUMA738 [47] (RFP) or pUMa791 or pUMA792 (Feldbrügge, unpublished) for 3×HA fusions. Fusion constructs were subcloned in pCRII TOPO (Invitrogen), PCR-generated linear DNA was used for transformation of *U. maydis*. Homologous integration of the constructs was verified by Southern analysis. All sequences of oligonucleotides used for PCR are listed in Table S9.

Total RNA was extracted using Trizol reagent (Invitrogen) according to the manufacturer's instructions. RNA samples to be used for real-time qRT-PCR or mRNA-Seq analysis were column purified (RNeasy; Qiagen) and the quality was checked using a Bioanalyzer with an RNA 6000 Nano LabChip kit (Agilent). Quantitative RT-PCR analysis for *pcna*, *rpb3*, *ubi1*, *rho3* and *rbf1* was conducted with oligonucleotide pairs that specifically detected spliced and unspliced RNA species, respectively: forward primers used for quantification of spliced transcript were specific for exon/intron borders, whereas forward primers for the unspliced transcripts were placed within the intron sequence. Ratios of spliced vs. unspliced RNA of AB31 (wild-type) samples were set to 1.0 and compared to the ratios obtained with the AB31*Δnum*1. qRT-PCR experiments were done in three independent biological and two technical replicates each and followed the protocols given in [102] and [103].

DNA content was measured by flow cytometry. FACS analyses were performed according to [104] with the exception that 10^5^ cells were harvested for each time point and analyzed with a BD Biosciences FACSVerse flow cytometer using the FACSDiva 6.2 software. For each acquisition, 2*10^4^ events were measured at an average flow rate of 100–500 events per second.

### mRNA-sequencing

mRNA was sequenced starting from total RNA isolated from strains AB31 and AB31*Δnum1* on an Illumina HiSeq 1000 using the TrueSeq mRNA sequencing kit according to the manufacturer's instructions. Three independent libraries for each of the two strains were generated (Olivier Armant, Insitute for Toxology and Genetics, KIT Campus North), using three independently grown cultures (biological replicates). Each library yielded an average of 60 million paired-end reads of 2×56 bp, which equals about 20×10^9^ bp sequences for each strain. Raw paired-end reads (2×56 bp) were aligned with TopHat 1.3.2 [105] to the *Ustilago* genome assembly provided by the MIPS *Ustilago maydis* Database (MUMDB, http://mips.helmholtz-muenchen.de/genre/proj/ustilago). TopHat was supplied with a gtf file derived from MUMDB's p3_t237631_Ust_maydi_20110629.gtf, but not restricted to known transcripts. Aligned reads were counted using custom Python scripts using the pysam library (http://code.google.com/p/pysam/). Gene expression values were derived by counting read pairs that were completely inside the exon regions of MUMDB-genes and differential expression was assessed with DESeq 1.6.1 [106] and BaySeq 1.8.1 [107] and filtered for an adjusted p-value≤0.05 and a more than two fold change.

For comparison and visualization of splicing efficiency, read pairs overlapping the respective intron/exon with their first segment by at least three base pairs were counted and normalized to fragments per kb per million (FPKM). Intron retention was determined by calculating the ratio of introns FPKM to the gene's overall exon FPKM. Exon FPKM included all exons with more than 10 bp. Only genes with an FPKM above 10 were considered. A small number of introns with a retention rate above 0.9 were excluded, based on the assumption that they base on false gene models. Sequencing data was deposited at the EBI ArrayExpress Database (E-MTAB-1300).

### Co-immunoprecipitation analysis

For *in vitro* protein expression and co-immunoprecipitation of Num1 and Kin1, the TNT quick coupled transcription/translation system (Promega) was used according to the manufacturer's protocol. For expression of Myc-tagged Num1 protein, plasmid pGBKT7-Num1 was used and HA-tagged Kin1 protein was generated using the pGAD-Kin1_650–968_ derivative identified in the yeast two-hybrid screen. All subsequent steps were carried out as described previously [103].

For *in vivo* co-immunoprecipitation, *U. maydis* sporidia were grown in 100 ml CM–G to an OD_600_ of 0.8. Cells were washed once with PBS buffer, resuspended in 1 ml PBS supplemented with Complete proteinase inhibitor cocktail (Roche), frozen in liquid nitrogen and homogenized in a Retsch mill for 10 minutes at 30 Hz. The cell lysate was incubated with 40 µg monoclonal anti-HA coupled agarose (Sigma-Aldrich) on a rotating wheel at 4°C over night. Agarose beads were washed three times in PBS prior to resuspension in 20 µl Laemmli buffer. Samples were boiled for three minutes and separated by SDS-PAGE. For co-immunoprecipitation of Num1 and Kin1, a crosslinking reaction with dithiobis[succinimidylpropionate] (Thermo Fisher) was conducted according to [108] using 300 ml of cells grown in CM-G to an OD_600_ of 0.8.

Proteins were transferred to PVDF nitrocellulose membranes in a semidry blot chamber. Western blots were probed with monoclonal anti-HA, anti-c-Myc, and anti-GFP (Sigma-Aldrich) antibodies. Horseradish peroxidase–conjugated anti-mouse or anti-rabbit IgG (Promega) was used as secondary antibody, and an ECL system was used for protein detection.

### Microscopy, image processing and quantitative analysis

For microscopic analyses, logarithmically growing *U. maydis* cells were taken from liquid cultures grown in CM-G medium. For the induction of hyphal growth, cells were shifted to CM-A or NM to induce the *P_crg1_*- or *P_nar1_*- promoters, respectively, for 12–14 hours. For microscopy of *A. nidulans* germlings and young hyphae, MM on cover slips was inoculated with a small amount of spores and incubated for 12–18 hours at 37°C. Cells were then placed on top of a 2% agarose cushion placed on the microscope slide and immediately observed using an Axioimager Z1 microscope equipped with an Axiocam MRm camera (Carl Zeiss). Standard filter sets for DAPI, GFP, CFP and Rhodamine were used for epifluorescence analysis.

Nuclei were stained with DAPI Vectashield H-1200 (Vector Laboratories); chitin was stained with 2 µg/ml Calcofluor White (Sigma-Aldrich) or with Congo Red solution (1 µl/ml; stock: 1 mg/ml in H_2_O). Congo Red was incubated at 25°C for 10 minutes on a turning wheel and washed twice with CM prior to microscopic analysis. Fungal cell walls in general were stained with 1 mg/ml WGA/Fluorescein (Invitrogen) in PBS. For the visualization of vacuoles, growing cells were incubated in CM supplemented with 10 µg/ml CellTracker Blue (7-amino-4-chloromethyl-coumarin, CMAC) (Invitrogen) for 30 minutes at 28°C, washed twice with CM and then subjected to microscopy. Chlorazole Black E staining was performed according to [24].

Endosome motility was measured in image sequences of 50 frames, taken with an exposure time of 500 msec. Only organelles that were moving for a distance of at least 5 µm were considered. The resulting movies were converted into kymographs using ImageJ software [109]. For quantification of Mcs1 signal intensity, the corrected total cell fluorescence (CTCF) of mid-size growing buds was calculated with ImageJ software (CTCF = integrated density – (area of selected cell×fluorescence of background reading)).

For quantification of the microtubule orientation, cells were grown to mid-log phase in YEPSL medium. Cells were analyzed via 45 sec time-lapse recordings (four frames each 15 sec). Pictures were taken with an exposure time of 90 msec for Tub1-GFP and 300 msec for Peb1:RFP signals. Movement of Peb1:RFP signals was scored at a screen as described in [46]. All image processing, including adjustment of brightness, contrast and gamma-values was performed with the AxioVision and ZEN software (Carl Zeiss), respectively.

### Phylogenetic analysis

For comparative phylogenetic analysis of Num1, 65 sequences with the highest similarity to Num1 were obtained by BLASTP analysis. The sequence of *Saccharomyces cerevisiae* Snt309p was included as outgroup. Sequences were aligned with MAFFT version 6 [110] using the global alignment G-INS-i. A phylogenetic tree was calculated using the minimum linkage clustering method (http://align.bmr.kyushu-u.ac.jp/mafft/online/server/).

Fig Tree 1.3 (http://tree.bio.ed.ac.uk/software/figtree/) was used to visualize the Nexus formats of the MAFFT results.

### Accession numbers

Sequence data from this article can be found at the Munich Information Center for Protein Sequences (MIPS) *Ustilago maydis* database (http://mips. helmholtz-muenchen.de/genre/proj/ustilago/), the *Aspergillus* genome database (http://www.aspgd.org/) and the National Center for Biotechnology Information (NCBI) database under the following accession numbers: *num1* (*um01682*), XP_757829; *prp19* (*um10027*), XP_756259; *cef1* (*um04411*), XP_760558; *rbf1* (*um03172*), XP_759319; *pcna* (*um05403*), XP_761550; *rpb3* (*um03550*), XP_759697; *ubi1* (*um02440*), XP_758587; *rho3* (*um04070*), XP_760217; *rad51* (*um03290*), XP_759437; *rec1* (*um11016*), XP_759527.1; *mms2* (*um10097*), XP_756467.1; *cks1* (*um03210*), XP_759357; *cdk1* (*um10705*), AAP94021.1; *hdp1* (*um12024*), XP_761909.1; *hdp2* (*um04928*), XP_761075; *biz1* (*um02549*), XP_758696; *um02704* (related to allantoate permease), XP_758851; *um12105* (probable PUP3 – 20S proteasome subunit), XP_756658.1; *actin* (*um11232*), XP_762364; *eIF2b* (*um04869*), XP_761016; *kin1* (*um04218*), XP_760356; *dyn2* (*um04372*), XP_760519; *yup1* (*um05406*), XP_761553; *tub1* (*um01221*), XP_757368; *AN4244*, XP_661848.1; *nudA* (*AN0118*), XP_657722.1; *kinA* (*AN5343*), XP_662947.1.; the gene model for *um15049* (related to PUF3 transcript specific regulator of mRNA degradation) is implemented only at MUMDB (http://mips.helmholtz-muenchen.de/genre/proj/ustilago), as the predicted protein is composed of two fragments located on adjacent contig ends and, according to the sequence gap, part of the protein may be absent.

## Supporting Information

Figure S1 Alignment of Num1 to SPF27 homologues. ClustalW was used to align proteins related to Num1 from *S. reilianum* (Sr12752, NCBI accession number CBQ71896), *U. hordei* (UHOR_02497, CCF53376) *C. cinerea* (Num1, BAC78620), *A. nidulans* (AN4244, XP_661848.1), *A. thaliana* (Mos4, AT3G18165.1), mouse (SPF27, BAB31409) and human (BCAS2/SPF27, CAG46834). Identical or similar residues are highlighted in black and grey, respectively. The nuclear localization signal (NLS) in the *U. maydis* sequence is highlighted in green. The red box indicates the conserved BCAS2-domain.
(PDF)

Figure S2 Phylogeny of Num1 homologues. For comparative phylogenetic analysis of Num1, 65 sequences with the highest similarity to Num1 were obtained by BLASTP analysis. The sequence of *Saccharomyces cerevisiae* Snt309p was included as out-group. Sequences were aligned with MAFFT version 6 [110] using the global alignment G-INS-i. A phylogenetic tree was calculated using the minimum linkage clustering method.
(PDF)

Figure S3 Deletion of *num1* does not affect growth in axenic culture, mating and dikaryon formation or proliferation *in planta*. (**A**) Growth curves of AB31 and AB31*Δnum1* deletion strain in YEPSL complex- (black) and glutamin/glucose-containing minimal medium (grey). The *num1*-deletion has no influence on growth in axenic culture. (**B**) SG200 (control) and SG200*Δnum1* sporidia were grown in glucose-containing CM-medium. No obvious phenotype could be observed, indicating that the *Δnum1*-mutant is not impaired during growth in axenic culture. (**C**) Mating assays on charcoal-containing CM-glucose medium by co-spotting of strains indicated. FB1 (*a1b1*) and FB2 (*a2b2*) strains are included as wild-type controls. Formation of dikaryotic aerial hyphae is visible as white mycelium. The *num1*-deletion has no influence on the formation of the dikaryon. (**D**) Pathogenicity of individual *num1*-deletion strains. Disease rating of maize seedlings seven days post inoculation with *U. maydis* strains SG200, SG200*Δnum1*, FB1×FB2 (wild-type crosses) and FB1*Δnum1×*FB2*Δnum1*-derivatives. #1, #2 indicate independently obtained deletion mutants. Bars represent the percentage of infected plants with the symptoms indicated in the legend. N corresponds to the total number of plants infected. (**E**) Maize plants were inoculated with SG200 and SG200*Δnum1*. Infected leaves were stained with Chlorazole Black E. Samples were taken three and five days post inoculation, respectively. Clamp-like structures (arrowheads) could be observed in both strains, indicating fungal proliferation within the plant cells. Scale bars: 10 µm.
(PDF)

Figure S4 Phenotype of conjugation hyphae of *num1*-deletion strains.. Strains FB1 and FB1*Δnum1* were treated with synthetic a2-pheromone (2,5 µg/ml) for six hours in glucose-containing CM-medium. DMSO was used as a solvent for the a2-pheromone and served as negative control. Both FB1 and FB1*Δnum1* respond to the pheromone with the formation of conjugation tubes. Note the characteristic *Δnum1*-mutant phenotype, such as branches and irregular hyphal growth. Scale bars: 10 µm.
(PDF)

Figure S5 Phenotype of dikaryotic hyphae of *num1*-deletion strains. Compatible FB1- and FB2-strains were used to monitor the *Δnum1*-phenotype in dikaryotic hyphae. To demonstrate successful fusion events, FB1-strains express mitochondrial matrix-targeted RFP (mtRFP), and FB2-strains express mitochondrial matrix-targeted GFP (mtGFP), respectively, under control of the inducible *P_crg1_*-promoter. Fusion of the strains was induced in arabinose-containing liquid charcoal medium overnight. Depicted are DIC-, GFP- and RFP-signals. Merged images show both fluorescent signals within the same hypha, indicating fusion of two sporidia in compatible FB1×FB2 wild-type crossings (upper panel) as well as in compatible FB1*Δnum1×*FB2*Δnum1* crossings (lower panel). The two initial cells are marked with an asterisk. Note the characteristic *Δnum1*-mutant phenotype (thicker, irregular hyphal growth and bipolar growth of the sporidia). Scale bars: 10 µm.
(PDF)

Figure S6 The *Δnum1*-deletion causes chitin accumulations and the insertion of unusual septa. Induction of the bE1/bW2 heterodimer in wild-type cells (AB31) induces a G2 cell cycle arrest, resulting in hyphae with a single nucleus positioned in the tip compartment. 18% of AB31*Δnum1* mutant hyphae contain more than one nucleus within the cell. Filaments were stained with DAPI to visualize nuclei and Fluorescein-conjugated wheat germ agglutinin (WGA), which specifically binds to *N*-acetylglucosamine [27]. In AB31 wild-type cells, chitin was intensively stained in the growth region within the hyphal apex as well as in the basal retraction septum (marked with arrowhead). In AB31*Δnum1* mutants, however, chitin accumulations along the hyphae (marked by asterisks) as well as septa-like chitin rings were observed frequently (middle and lower panel). In many cases, these septa were not visible in the DIC channel, indicating that these structures may not represent true septal cell walls. Scale bars: 10 µm. Septa in AB31*Δnum1* were also visualized by Calcofluor (Figures 1E and 6B) and Congo Red (Figure 3A). In contrast to septa in the dikaryotic hyphae, septa in AB31*Δnum1* were not always visible in the DIC channel, indicating that these septa or septa-like structures may be structurally different from wild-type septa.
(PDF)

Figure S7 Complementation analyses of *num1*-deletion strains. Filaments of AB31 and AB31*Δnum1*, expressing Num1:eGFP fusion proteins under control of the native *P_num1_*- or the inducible *P_crg1_*-promoter, were analyzed 12–14 hours after induction of the bE1/bW2-heterodimer and, when present, the Num1:eGFP fusion proteins. (**A**) In AB31, Num1:eGFP fusion proteins localize predominantly to the nucleus. (**B**) Complementation of the *num1*-deletion with a Num1:eGFP fusion protein restores the wild-type phenotype and the protein localizes predominantly to the nucleus. (**C**) A Num1:3eGFP fusion protein with mutated nuclear localization signal (NLS, shown in **D**) does not complement the *Δnum1* phenotype; filaments show the typical curved and bipolar morphology of the *num1*-deletion. In addition to the GFP-signal in the nucleus, a strong signal is observed in the cytoplasm. Scale bars: 10 µm. (**D**) Schematic representation of the Num1 protein depicting the basic amino acids within the NLS that were replaced by alanine-residues.
(PDF)

Figure S8 Prp19- and Cef1-fusion proteins are functional and are localized in the nucleus. *prp19* and *cef1* were expressed as C-terminal RFP- or HA-tagged versions under control of their endogenous promoters in strain AB31 to ensure native expression levels. Hyphal growth was analyzed 14 hours after induction of the bE1/bW2-heterodimer. (**A**) Shown are DIC- and RFP-fluorescence signals of strains UNK208 (AB31 *num1:3egfp:hyg^R^, prp19:rfp:nat^R^*) and (**B**) UMO10 (AB31 *num1:3egfp:hyg^R^, cef1:rfp:nat^R^*). As *prp19* and *cef1* are both essential genes (see Supporting Text S1), the viability of the strains proofs the functionality of the fusion proteins. In addition, no altered phenotypes with respect to filamentous growth were observed, corroborating that both Prp19:RFP as well as Cef1:RFP fusion proteins are functional. Scale bars: 10 µm.
(PDF)

Figure S9 Cell cycle analysis of AB31 vs. AB31*Δnum1*. (**A**) FACS analysis of AB31 and AB31*Δnum1* after induction of the bE1/bW2-heterodimer in arabinose-containing CM-medium. Samples were taken at time points indicated. The histograms show the DNA-content measured by FACS analysis. Relative fluorescence intensities are given on horizontal axes, vertical axes reflect cell numbers. In AB31, induction of the active bE1/bW2-combination resulted in an enrichment of cells with a 2C DNA-content, indicative of a G2 cell cycle arrest. In AB31*Δnum1*, a higher amount of cells in G1 was observed, which implicates defects in cell cycle regulation. (**B**) Microscopic analysis of the filamentous growth of the strains upon *b*-induction. Scale bars: 10 µm.
(PDF)

Figure S10 Control of *bW*-, *rbf1*- and *num1*-gene expression in samples used for RNA-Seq-analysis. RNA was isolated from strains AB31 and AB31*Δnum1* in three biological replicates each, eight hours after induction of the bE1/bW2-heterodimer in arabinose-containing minimal medium. Expression of *bW*-, *rbf1*- and *num1* was monitored by qRT-PCR analysis. Gene expression is shown relative to the highest expression value. *Actin* and *eIF2b* were used for normalization. Shown are the mean values two technical replicates. Error bars represent the SD.
(PDF)

Figure S11 Verification of mRNA-Seq using qRT-PCR. (**A**) Plot depicting splicing efficiency of genes based on the RNA-Seq analysis. Plotted are the FPKM values (fragments per kilobase of sequence per million fragments mapped) across the genomic region indicated (coordinates in nucleotides) of three independent RNA-Seq experiments for AB31 wild-type (blue lines) and AB31*Δnum1* (red lines), respectively. Exons (E) and introns (I) are indicated. (**B**) Schematic representation of exon-intron structures. Oligonucleotides used for qRT-PCR are depicted as arrows and allow the discrimination against spliced (primer depicted in red) and unspliced transcripts (primer depicted in green). (**C**) Gene expression analyses to verify expression of spliced vs. unspliced transcripts of the indicated genes using qRT-PCR. RNA was isolated from AB31 and AB31*Δnum1* eight hours after induction of the bE/bW-heterodimer. *Actin* and *eIF2b* were used for normalization. Depicted are the ratios of spliced vs. unspliced transcripts of three independent biological replicates. Mean values of two technical replicates each are shown.
(PDF)

Figure S12 Individual genes show different alterations in intron retention in AB31*Δnum1*. Splicing efficiency of the indicated genes based on the RNA-Seq analysis. Depicted are examples for genes (**A**) where splicing is not affected, (**B**) the intron is not spliced at all, or (**C**) different introns show dissimilar retention rates. Plotted are the FPKM values (fragments per kilobase of sequence per million fragments mapped) across the genomic region indicated (coordinates in nucleotides) of three independent RNA-Seq experiments for AB31 wild-type (blue lines) and AB31*Δnum1* (red lines), respectively. Exons (E) and introns (I) are indicated.
(PDF)

Figure S13 Reduced splicing efficiency in AB31*Δnum1* in genes with function in DNA repair and cell cycle. Splicing efficiency based on RNA-Seq analysis. Depicted are examples for genes grouping in the functional category (**A**) “DNA repair” and (**B**) “cell cycle”. Plotted are the FPKM values (fragments per kilobase of sequence per million fragments mapped) across the genomic region indicated (coordinates in nucleotides) of three independent RNA-Seq experiments for AB31 wild-type (blue lines) and AB31*Δnum1* (red lines), respectively. Exons (E) and introns (I) are indicated.
(PDF)

Figure S14 Splicing defect of the *rbf1*-gene in AB31*Δnum1*. (**A**) Schematic view of *rbf1* intron/exon structure and primers used for RT-PCR (blue) and qRT-PCR (red, green and black). (**B**) RNA samples of AB31 and AB31*Δnum1* were isolated eight hours after induction of the bE1/bW2-heterodimer. RT-PCR analysis was performed on the *rbf1* open reading frame and PCR products were separated on a 2% TAE-agarose gel. In AB31 cDNA, the spliced version of *rbf1* (430 bp) is predominantly detected. In AB31*Δnum1* more bands spanning different sizes from 430 bp (all four introns spliced) to 1192 bp (all introns retained) are observed, validating the splicing defect of the *rbf1*-gene observed in the RNA-Seq analysis. (**C**) qRT-PCR analysis to investigate the expression of spliced vs. unspliced *rbf1*-transcripts. Primers depicted in (A) were used to distinguish spliced (primer depicted in red) from non-spliced (primer depicted in green) introns. *Actin* and *eIF2b* were used for normalization. Depicted are the ratios of spliced vs. unspliced transcripts of three independent biological replicates. Mean values of two technical replicates each are shown.
(PDF)

Figure S15 Splicing efficiency and expression profile of the Rbf1-target genes *hdp1*, *hdp2*, *biz1* is altered in AB31*Δnum1*. Shown are the expression profiles based on the RNA-Seq analysis. Plotted are the FPKM values (fragments per kilobase of sequence per million fragments mapped) across the genomic region indicated (coordinates in nucleotides) of three independent RNA-Seq experiments for AB31 wild-type (blue lines) and AB31*Δnum1* (red lines), respectively. Exons (E) and introns (I) are indicated. (**A**) Expression of the *hdp1*-gene is increased about two-fold, and splicing efficiency of both introns is reduced in AB31*Δnum1*. (**B**) and (**C**) Expression levels of *hdp2* as well as *biz1* are significantly reduced in AB31*Δnum1* (*hdp2*: −3,1-fold, p = 5.28 * 10^−36^ *biz1*: −3,4-fold, p = 5.34 * 10^−62^; significance according to DEseq [106]).
(PDF)

Figure S16 Introns are essential for full function of *rbf1*. (**A**) and (**B**) An intron-free derivative of *rbf1* was expressed in AB31, replacing the endogenous *rbf1*-gene. cDNA was isolated from strains AB31 and UTO62 (AB31 *rbf1::rbf1cDNA*) 10 hours after induction of filamentous growth in arabinose-containing CM-medium. qRT-PCR analysis was performed to verify unaltered expression of *bE* and *rbf1* (**A**). The intron-free *rbf1*-derivative is not able to induce *rbf1*-target genes (**B**). Gene expression is shown relative to the highest expression value. *Actin* and *eIF2b* were used for normalization. Mean values of two technical replicates each are shown. (**C**) Schematic representation of the construct used in this study. *rbf1*-cDNA was expressed in the genomic *rbf1*-locus under control of the endogenous *P_rbf1_*-promoter. 500 bp of the 3′-UTR were included as terminator. A carboxin resistance-cassette was used for selection. (**D**) SG200-derivatives were spotted on charcoal-containing CM-medium and incubated at 22°C for 3 days. The formation of filamentous hyphae is visible as white mycelium. In contrast to the complementation with a wild-type *rbf1*-copy [2], the respective intron-free *rbf1*-derivative, which replaced the endogenous *rbf1*, was not able to complement the *Δrbf1*-phenotype. Magnified images of the colony margins were taken with a binocular assisted camera. (**E**) Maize seedlings were infected with the indicated SG200 derivatives. Disease rating and tumor formation was monitored seven days after infection. Bars represent the percentage of infected plants with symptoms indicated in the legend. N corresponds to the number of plants infected. The intron-free *rbf1*-derivative leads to a reduction in tumor formation. (**F**) and (**G**) Microscopic analysis of the indicated strains 10 hours after induction of filamentous growth in arabinose-containing CM-medium. Phenotypes were grouped into four different categories. Whereas 95% of AB31 wild-type cells grew as long, straight filaments, the intron-free *rbf1*-derivative was not able to fully complement the *Δrbf1*-phenotype. Integration of the *rbf1*-cDNA construct into the Δ*num1*-mutant background hence did not reduce the observed phenotypes of the *num1*-deletion strain. (**G**) Representative microscopic images of the indicated strains. Shown are DIC-images. Scale bars: 10 µm.
(PDF)

Figure S17 Characterization of the interaction domain of Num1 for the Kin1 motor protein. N- and C-terminally truncated fragments of the Num1-protein were tested for interaction with Kin1 in the yeast two-hybrid system. Left panel: Schematic depiction of the constructs used in this study. Numbers represent the regions of the Num1-constructs in amino acids. The conserved BCAS2-domain is depicted in dark grey. After cloning into pGBKT7 (Clontech), the constructs were co-transformed with the pGAD-Kin1_650–968_ fragment isolated in the yeast two-hybrid screen. Serial 10-fold dilutions of cell suspensions of the respective transformants were spotted on selective media (SD –LW) and stringent media (SD –LWHA), indicating an interaction. Plates were incubated for two days at 30°C. The Num1 interaction domain resides between amino acids 67 and 147 (highlighted in red). pGBKT7-p53 with pGADT7-T served as positive control, pGBKT7-Lam with pGADT7-T (Clontech) served as negative control.
(PDF)

Figure S18 The double deletion of Num1 and Kin1 has additive effects on pathogenicity as well as hyphal morphology. (**A**) Effects of the double deletion on polar hyphal growth in strain AB31*Δnum1Δkin1*. Filaments of were analyzed 12 hours after induction of the bE1/bW2-heterodimer. Compared to AB31*Δkin1* and AB31*Δnum1* hyphae, which predominantly grow as bipolar filaments, the double deletion has additive effects on filament morphology, polarity and branching. (**B**) To visualize nuclear positioning and septation, filaments of AB31*Δnum1Δkin1* were treated with DAPI- and WGA/FITC. Scale bars: 10 µm. (**C**) Pathogenicity of individual *num1*- and *kin1*-deletion strains. Maize seedlings were infected with the indicated SG200 derivatives. #1, #2, #3 indicate independently obtained deletion strains. Disease rating and tumor formation was monitored seven days after inoculation. Bars represent the percentage of infected plants with symptoms indicated in the legend. N corresponds to the total number of plants infected. Deletion of the individual *num1* or *kin1* genes results in slightly reduced pathogenicity, whereas the *num1/kin1*-double deletion has a much stronger effect on pathogenicity. Depicted below are disease symptoms (third leaf below the site of injection) of representative maize plants seven days after inoculation.
(PDF)

Figure S19 Num1 has neither influence on morphology or abundance of microtubules and actin elements, nor on the distribution of the Kin1 motor protein. Microtubules were analyzed using a Tub1:mCherry fusion protein, the actin cytoskeleton was visualized using the lifeact-method (lifeact:yfp) [44]. Both fusion proteins were expressed under control of the constitutively active *P_otef_*-promoter in strains AB31 and AB31*Δnum1*. For the localization of the Kin1 motor protein in strains AB33 and AB33*Δnum1*, an N-terminal 3eGFP:Kin1 fusion was used [14]. The fluorescent cytoskeletal elements were microscopically analyzed 12–14 hours after induction of the bE1/bW2-heterodimer in arabinose-containing CM-medium for AB31 and nitrate-containing minimal-medium for AB33, respectively. mCherry, YFP- and GFP-signals are shown. Scale bars: 10 µm.
(PDF)

Figure S20 Orientation of the microtubule cytoskeleton in AB33 and AB33*Δnum1* sporidia and hyphae. Orientation of the microtubule cytoskeleton was addressed in strain AB33Peb1R_GT (AB33 *peb1:mRFP potefGFP:Tub1*) [14] and its *Δnum1* derivative. In AB33Peb1R_GT wild-type cells, the homologue of the microtubule (MT) plus-end marker EB1, Peb1, fused to RFP, localizes to growing MT plus-ends [45], which are predominantly oriented towards the cell poles of the hyphae [9], [14], or, in interphase cells, towards the budding cell and the opposing cell pole of the mother cell [46]. (**A**) Motility of Peb1:RFP in budded cells of AB33Peb1R_GT (WT) and its *Δnum1* derivative. Peb1:RFP binds to plus-ends of microtubules, and its motility (pictures were taken in 15 sec intervals) indicates the orientation of the microtubule [46]. Arrowheads and asterisks indicate individual Peb1:RFP dots. Scale bar: 2 µm (**B**) Quantification of microtubule orientation in interphase sporidia. AB33Peb1R_GT (WT) and its *Δnum1* derivative were grown in YEPSL medium to mid log phase. Black arrows and numbers above and below the cartoons give the percentage of Peb1-RFP dots moving to either tip of the cell. White arrows show the relative amount of Peb1-RFP to the bud neck. Numbers are based on 45 sec intervals of 30 and 46 cells in wild-type and *Δnum1*-strains, respectively. The total number of signals used for quantification and the number of cells is given in brackets. Quantification was done according to the protocol given in [46]. (**C**) In AB33Peb1R_GT wild-type hyphae, Peb1-RFP is predominantly oriented towards the cell poles. (**D**) In AB33Peb1R_GT *Δnum1*, Peb1:RFP labeled MT plus-ends are also found at septa (marked by arrowheads), which are often placed in the middle of the hyphae. In some hyphae, mitotic spindles were observed (marked by asterisks), implicating that the *num1*-deletion leads to a deregulated cell cycle arrest. Peb1-RFP and GFP-Tub1 fluorescence was analyzed 8–10 hours after induction of hyphal growth in nitrate-containing minimal medium. Scale bars: 10 µm. (**E**) Schematic representation of the orientation of the microtubule cytoskeleton in wild-type and Δ*num1*-hyphae. Peb1-localization indicates microtubule plus-ends.
(PDF)

Figure S21 Influence of Num1 on the localization of the myosin chitin synthase Mcs1. (**A**) In wild-type cells, 3eGFP:Mcs1 localizes to growing bud tips, where it participates in the synthesis of chitin [15]. In contrast, *Δnum1*-mutants shows reduced Mcs1-accumulation at the growth region. Shown are DIC and GFP-images, as well as contrast-inverted images. Scale bars: 5 µm. (**B**) Quantitative analysis of the 3eGFP:Mcs1 signal intensity in growing bud cells. The bar diagram shows corrected total cell fluorescence (CTCF) of wild-type (control) and *Δnum1*-mutant cells. ImageJ software was used for quantification. N corresponds to the number of cells analyzed. (**C**) In AB33 hyphae, 3eGFP:Mcs1 localizes to distinct foci close to the cell membrane and forms a gradient towards the growth zone within the hyphal apex or localizes to the basal retraction septum (arrowhead), respectively. In contrast, in AB33*Δnum1*, no tip-ward gradient is obvious and fewer foci are observed at the cell membrane. In many cases Mcs1 does not localize to delocalized septa in the *Δnum1*-mutant (lower left panel, arrowhead indicates a septum). Mcs1-fluorescence was analyzed 12 hours after induction of hyphal growth in nitrate-containing minimal medium. Scale bars: 10 µm. (**D**) Enlarged images of the sections marked in (**C**). Images were contrast-inverted.
(PDF)

Figure S22 Num1 influences the dynamic of the RNA-binding protein Rrm4. To analyze the impact of *num1* on Rrm4 movement, an Rrm4:eGFP fusion protein was expressed in AB33 and AB33*Δnum1*. *bE1/bW2* were induced for 16 hours in nitrate minimal medium, and Rrm4:eGFP localization was analyzed by fluorescence microscopy. (**A**) In AB33, Rrm4:eGFP-fusion proteins localize on bidirectional moving particles that are evenly distributed within the hyphae, as previously described [46]; see Supporting Video S3. (**B**) In AB33Δ*num1* the Rrm4-particles accumulate at the hyphal tip (asterisk, upper panel) or within the hyphae (lower panel); see also Supporting Video S4 Arrowhead indicates septum. Scale bars: 10 µm.
(PDF)

Video S1 Motility of Yup1-labeled early endosomes in AB33. Visualization of early endosomes in strain AB33 using a Yup1:eGFP-fusion protein under control of the constitutively active *P_otef_*-promoter as a marker. Depicted is the GFP-fluorescence 14 h after induction of the bE/bW-heterodimer in nitrate-containing minimal medium. Rapid, bi-directional motility of early endosomes is visible throughout the filament. Immobile bright fluorescent spots most likely represent vacuoles, as Yup1 was also reported to bind to vacuolar membranes [4]. The image sequence was taken using an Axioimager.Z1 microscope equipped with an Axiocam MRm camera (Carl Zeiss). In total, 50 frames with an exposure time of 253 msec were taken every 0,46 sec for 23,173 sec. Time is given in hrs:min:sec.msec. The scale bar represents 10 µm.
(MOV)

Video S2 Motility of Yup1-labeled early endosomes in AB33*Δnum1*. Visualization of early endosomes in strain AB33*Δnum1* using the Yup1:eGFP-fusion protein under control of the constitutively active *P_otef_*-promoter as a marker. Depicted is the GFP-fluorescence 14 h after induction of the bE/bW-heterodimer in nitrate-containing minimal medium. Endosomal trafficking is considerably reduced and vesicles accumulate at hyphal tips as well as around delocalized septae. The image sequence was taken using an Axioimager.Z1 microscope equipped with an Axiocam MRm camera (Carl Zeiss). In total, 50 frames with an exposure time of 44 msec were taken every 0,26 sec for 12,891 sec. Time is given in hrs:min:sec.msec. The scale bar represents 10 µm.
(MOV)

Video S3 Motility of Rrm4-labeled particles in AB33. Visualization of the RNA-binding protein Rrm4 in strain AB33 using an Rrm4:eGFP fusion protein that was expressed under control of its native promoter. Depicted is the GFP-fluorescence 16 h after induction of the bE/bW-heterodimer in nitrate-containing minimal medium. The protein localizes to rapid, bi-directionally transported particles. The image sequence was taken using an Axioimager.Z1 microscope equipped with an Axiocam MRm camera (Carl Zeiss). In total, 20 frames with an exposure time of 500 msec were taken every 0,96 sec for 19,266 sec. Time is given in hrs:min:sec.msec. The scale bar represents 10 µm.
(MOV)

Video S4 Motility of Rrm4-labeled particles in AB33*Δnum1*. Visualization of the RNA-binding protein Rrm4 in strain AB33*Δnum1* using an Rrm4:eGFP fusion protein that was expressed under control of its native promoter. Depicted is the GFP-fluorescence 16 h after induction of the bE/bW-heterodimer in nitrate-containing minimal medium. The particles are frequently observed in accumulations at various parts of the hyphae and trafficking is considerably reduced. The image sequence was taken using an Axioimager.Z1 microscope equipped with an Axiocam MRm camera (Carl Zeiss). In total, 25 frames with an exposure time of 500 msec were taken every 1,16 sec for 29,141 sec. Time is given in hrs:min:sec.msec. The scale bar represents 10 µm.
(MOV)

Text S1 Prp19:3×HA and Cef1:3×HA fusion proteins are functional in *U. maydis*.
(DOCX)

Table S1 Intron retention in AB31 and AB31*Δnum1*.
(XLSX)

Table S2 Intron containing genes within the FunCats “cell cycle” or “DNA repair”.
(XLSX)

Table S3 Splicing defect upon *num1* deletion in genes encoding transcription factors.
(XLSX)

Table S4 Expression of b-target genes in AB31 compared to AB31*Δrbf1* and AB31*Δnum1*.
(XLSX)

Table S5 Altered gene expression in AB31*Δnum1*.
(XLSX)

Table S6 Num1 interacting proteins identified by Yeast Two-Hybrid screening.
(DOCX)

Table S7 *U. maydis* strains used in this study.
(DOCX)

Table S8 *A. nidulans* strains used in this study.
(DOCX)

Table S9 Oligonucleotides used in this study.
(DOCX)

## References

[1] Brefort T, Doehlemann G, Mendoza-Mendoza A, Reissmann S, Djamei A, et al (2009) *Ustilago maydis* as a Pathogen. Annu Rev Phytopathol 47: 423–445. 19400641 10.1146/annurev-phyto-080508-081923

[2] Heimel K, Scherer M, Vranes M, Wahl R, Pothiratana C, et al (2010) The transcription factor Rbf1 is the master regulator for *b*-mating type controlled pathogenic development in *Ustilago maydis* . PLoS Pathog 6: e1001035. 20700446 10.1371/journal.ppat.1001035 PMC2916880

[3] Steinberg G, Perez-Martin J (2008) *Ustilago maydis*, a new fungal model system for cell biology. Trends Cell Biol 18: 61–67. 18243705 10.1016/j.tcb.2007.11.008

[4] Wedlich-Söldner R, Bölker M, Kahmann R, Steinberg G (2000) A putative endosomal t-SNARE links exo- and endocytosis in the phytopathogenic fungus *Ustilago maydis* . EMBO J 19: 1974–1986. 10790364 10.1093/emboj/19.9.1974 PMC305698

[5] Straube A, Enard W, Berner A, Wedlich-Söldner R, Kahmann R, et al (2001) A split motor domain in a cytoplasmic dynein. EMBO J 20: 5091–5100. 11566874 10.1093/emboj/20.18.5091 PMC125636

[6] Vollmeister E, Schipper K, Feldbrügge M (2012) Microtubule-dependent mRNA transport in the model microorganism *Ustilago maydis* . RNA Biol 9: 261–268. 22336706 10.4161/rna.19432

[7] Baumann S, Pohlmann T, Jungbluth M, Brachmann A, Feldbrügge M (2012) Kinesin-3 and dynein mediate microtubule-dependent co-transport of mRNPs and endosomes. J Cell Sci 125 Pt 11: 2740–52. 22357951 10.1242/jcs.101212

[8] Wedlich-Söldner R, Straube A, Friedrich MW, Steinberg G (2002) A balance of KIF1A-like kinesin and dynein organizes early endosomes in the fungus *Ustilago maydis* . EMBO J 21: 2946–2957. 12065408 10.1093/emboj/cdf296 PMC126054

[9] Lenz JH, Schuchardt I, Straube A, Steinberg G (2006) A dynein loading zone for retrograde endosome motility at microtubule plus-ends. EMBO J 25: 2275–2286. 16688221 10.1038/sj.emboj.7601119 PMC1478194

[10] Schuster M, Kilaru S, Fink G, Collemare J, Roger Y, et al (2011) Kinesin-3 and dynein cooperate in long-range retrograde endosome motility along a nonuniform microtubule array. Mol Biol Cell 22: 3645–3657. 21832152 10.1091/mbc.E11-03-0217 PMC3183019

[11] Schuster M, Lipowsky R, Assmann MA, Lenz P, Steinberg G (2011) Transient binding of dynein controls bidirectional long-range motility of early endosomes. Proc Natl Acad Sci U S A 108: 3618–3623. 21317367 10.1073/pnas.1015839108 PMC3048114

[12] Schuster M, Kilaru S, Ashwin P, Lin C, Severs NJ, et al (2011) Controlled and stochastic retention concentrates dynein at microtubule ends to keep endosomes on track. EMBO J 30: 652–664. 21278707 10.1038/emboj.2010.360 PMC3041956

[13] Steinberg G, Schliwa M, Lehmler C, Bölker M, Kahmann R, et al (1998) Kinesin from the plant pathogenic fungus *Ustilago maydis* is involved in vacuole formation and cytoplasmic migration. J Cell Sci 111 Pt 15: 2235–2246. 9664045 10.1242/jcs.111.15.2235

[14] Schuchardt I, Assmann D, Thines E, Schuberth C, Steinberg G (2005) Myosin-V, Kinesin-1, and Kinesin-3 cooperate in hyphal growth of the fungus *Ustilago maydis* . Mol Biol Cell 16: 5191–5201. 16120650 10.1091/mbc.E05-04-0272 PMC1266418

[15] Weber I, Assmann D, Thines E, Steinberg G (2006) Polar localizing class V myosin chitin synthases are essential during early plant infection in the plant pathogenic fungus *Ustilago maydis* . Plant Cell 18: 225–242. 16314447 10.1105/tpc.105.037341 PMC1323495

[16] Treitschke S, Doehlemann G, Schuster M, Steinberg G (2010) The myosin motor domain of fungal chitin synthase V is dispensable for vesicle motility but required for virulence of the maize pathogen *Ustilago maydis* . Plant Cell 22: 2476–2494. 20663961 10.1105/tpc.110.075028 PMC2929105

[17] Schuster M, Treitschke S, Kilaru S, Molloy J, Harmer NJ, et al (2012) Myosin-5, kinesin-1 and myosin-17 cooperate in secretion of fungal chitin synthase. EMBO J 31: 214–227. 22027862 10.1038/emboj.2011.361 PMC3252574

[18] Makino R, Kamada T (2004) Isolation and characterization of mutations that affect nuclear migration for dikaryosis in *Coprinus cinereus* . Curr Genet 45: 149–156. 14624309 10.1007/s00294-003-0466-4

[19] Neubauer G, King A, Rappsilber J, Calvio C, Watson M, et al (1998) Mass spectrometry and EST-database searching allows characterization of the multi-protein spliceosome complex. Nat Genet 20: 46–50. 9731529 10.1038/1700

[20] Lehmler C, Steinberg G, Snetselaar K, Schliwa M, Kahmann R, et al (1997) Identification of a motor protein required for filamentous growth in *Ustilago maydis* . EMBO J 16: 3464–3473. 9218789 10.1093/emboj/16.12.3464 PMC1169972

[21] Grote M, Wolf E, Will CL, Lemm I, Agafonov DE, et al (2010) Molecular architecture of the human Prp19/CDC5L complex. Mol Cell Biol 30: 2105–2119. 20176811 10.1128/MCB.01505-09 PMC2863591

[22] Chelsky D, Ralph R, Jonak G (1989) Sequence requirements for synthetic peptide-mediated translocation to the nucleus. Mol Cell Biol 9: 2487–2492. 2668735 10.1128/mcb.9.6.2487-2492.1989 PMC362321

[23] Kämper J, Kahmann R, Bölker M, Ma LJ, Brefort T, et al (2006) Insights from the genome of the biotrophic fungal plant pathogen *Ustilago maydis* . Nature 444: 97–101. 17080091 10.1038/nature05248

[24] Brachmann A, Weinzierl G, Kämper J, Kahmann R (2001) Identification of genes in the bW/bE regulatory cascade in *Ustilago maydis* . Mol Microbiol 42: 1047–1063. 11737646 10.1046/j.1365-2958.2001.02699.x

[25] Mitchison JM, Nurse P (1985) Growth in cell length in the fission yeast *Schizosaccharomyces pombe* . J Cell Sci 75: 357–376. 4044680 10.1242/jcs.75.1.357

[26] Matsuoka H, Yang H-C, Homma T, Nemoto Y, Yamada S, et al (1995) Use of Congo red as a microscopic fluorescence indicator of hyphal growth. Appl Microbiol Biotechnol 43: 102–108.

[27] Nagata Y, Burger MM (1974) Wheat germ agglutinin. Molecular characteristics and specificity for sugar binding. J Biol Chem 249: 3116–3122. 4830237

[28] Ohi MD, Gould KL (2002) Characterization of interactions among the Cef1p-Prp19p-associated splicing complex. Rna 8: 798–815. 12088152 10.1017/s1355838202025050 PMC1370298

[29] Nasmyth K, Nurse P (1981) Cell division cycle mutants altered in DNA replication and mitosis in the fission yeast *Schizosaccharomyces pombe* . Mol Gen Genetics 182: 119–124. 10.1007/BF00422777 6943408

[30] Ohi R, McCollum D, Hirani B, Den Haese GJ, Zhang X, et al (1994) The *Schizosaccharomyces pombe* cdc5+ gene encodes an essential protein with homology to c-Myb. EMBO J 13: 471–483. 8313892 10.1002/j.1460-2075.1994.tb06282.x PMC394831

[31] Bernstein HS, Coughlin SR (1998) A mammalian homolog of fission yeast Cdc5 regulates G2 progression and mitotic entry. J Biol Chem 273: 4666–4671. 9468527 10.1074/jbc.273.8.4666

[32] Henriques JA, Moustacchi E (1980) Isolation and characterization of pso mutants sensitive to photo-addition of psoralen derivatives in *Saccharomyces cerevisiae* . Genetics 95: 273–288. 7009316 10.1093/genetics/95.2.273 PMC1214226

[33] Grey M, Düsterhöft A, Henriques JA, Brendel M (1996) Allelism of PSO4 and PRP19 links pre-mRNA processing with recombination and error-prone DNA repair in *Saccharomyces cerevisiae* . Nucleic Acids Res 24: 4009–4014. 8918805 10.1093/nar/24.20.4009 PMC146181

[34] Brendel M, Bonatto D, Strauss M, Revers LF, Pungartnik C, et al (2003) Role of PSO genes in repair of DNA damage of *Saccharomyces cerevisiae* . Mutat Res 544: 179–193. 14644320 10.1016/j.mrrev.2003.06.018

[35] Zhang N, Kaur R, Lu X, Shen X, Li L, et al (2005) The Pso4 mRNA splicing and DNA repair complex interacts with WRN for processing of DNA interstrand cross-links. J Biol Chem 280: 40559–40567. 16223718 10.1074/jbc.M508453200

[36] Beck BD, Park SJ, Lee YJ, Roman Y, Hromas RA, et al (2008) Human Pso4 is a metnase (SETMAR)-binding partner that regulates metnase function in DNA repair. J Biol Chem 283: 9023–9030. 18263876 10.1074/jbc.M800150200 PMC2431028

[37] Lu X, Legerski RJ (2007) The Prp19/Pso4 core complex undergoes ubiquitylation and structural alterations in response to DNA damage. Biochem Biophys Res Commun 354: 968–974. 17276391 10.1016/j.bbrc.2007.01.097 PMC1810354

[38] Legerski RJ (2009) The Pso4 complex splices into the DNA damage response. Cell Cycle 8: 3448–3449. 19838058 10.4161/cc.8.21.9760

[39] Garcia-Muse T, Steinberg G, Perez-Martin J (2003) Pheromone-induced G2 arrest in the phytopathogenic fungus *Ustilago maydis* . Eukaryot Cell 2: 494–500. 12796294 10.1128/EC.2.3.494-500.2003 PMC161457

[40] Mazzei T (1984) Chemistry and mechanism of action of bleomycin. Chemioterapia : international journal of the Mediterranean Society of Chemotherapy 3: 316–319. 6085286

[41] Yarbro JW (1992) Mechanism of action of hydroxyurea. Semin Oncol 19: 1–10. 1641648

[42] Revers LF, Cardone JM, Bonatto D, Saffi J, Grey M, et al (2002) Thermoconditional modulation of the pleiotropic sensitivity phenotype by the *Saccharomyces cerevisiae* PRP19 mutant allele pso4-1. Nucleic Acids Res 30: 4993–5003. 12434004 10.1093/nar/gkf632 PMC137178

[43] Flor-Parra I, Vranes M, Kämper J, Perez-Martin J (2006) Biz1, a zinc finger protein required for plant invasion by *Ustilago maydis*, regulates the levels of a mitotic cyclin. Plant Cell 18: 2369–2387. 16905655 10.1105/tpc.106.042754 PMC1560913

[44] Banks GR, Shelton PA, Kanuga N, Holden DW, Spanos A (1993) The *Ustilago maydis nar1* gene encoding nitrate reductase activity: sequence and transcriptional regulation. Gene 131: 69–78. 8370542 10.1016/0378-1119(93)90670-x

[45] Riedl J, Crevenna AH, Kessenbrock K, Yu JH, Neukirchen D, et al (2008) Lifeact: a versatile marker to visualize F-actin. Nat Methods 5: 605–607. 18536722 10.1038/nmeth.1220 PMC2814344

[46] Straube A, Brill M, Oakley BR, Horio T, Steinberg G (2003) Microtubule organization requires cell cycle-dependent nucleation at dispersed cytoplasmic sites: polar and perinuclear microtubule organizing centers in the plant pathogen *Ustilago maydis* . Mol Biol Cell 14: 642–657. 12589060 10.1091/mbc.E02-08-0513 PMC149998

[47] Becht P, König J, Feldbrügge M (2006) The RNA-binding protein Rrm4 is essential for polarity in *Ustilago maydis* and shuttles along microtubules. J Cell Sci 119: 4964–4973. 17105762 10.1242/jcs.03287

[48] Requena N, Alberti-Segui C, Winzenburg E, Horn C, Schliwa M, et al (2001) Genetic evidence for a microtubule-destabilizing effect of conventional kinesin and analysis of its consequences for the control of nuclear distribution in *Aspergillus nidulans* . Mol Microbiol 42: 121–132. 11679072 10.1046/j.1365-2958.2001.02609.x

[49] Koncz C, Dejong F, Villacorta N, Szakonyi D, Koncz Z (2012) The spliceosome-activating complex: molecular mechanisms underlying the function of a pleiotropic regulator. Front Plant Sci 3: 9. 22639636 10.3389/fpls.2012.00009 PMC3355604

[50] Tarn WY, Hsu CH, Huang KT, Chen HR, Kao HY, et al (1994) Functional association of essential splicing factor(s) with PRP19 in a protein complex. EMBO J 13: 2421–2431. 8194532 10.1002/j.1460-2075.1994.tb06527.x PMC395108

[51] Hogg R, McGrail JC, O'Keefe RT (2010) The function of the NineTeen Complex (NTC) in regulating spliceosome conformations and fidelity during pre-mRNA splicing. Biochem Soc Trans 38: 1110–1115. 20659013 10.1042/BST0381110 PMC4234902

[52] Ohi MD, Vander Kooi CW, Rosenberg JA, Chazin WJ, Gould KL (2003) Structural insights into the U-box, a domain associated with multi-ubiquitination. Nat Struct Biol 10: 250–255. 12627222 10.1038/nsb906 PMC5881891

[53] Ohi MD, Vander Kooi CW, Rosenberg JA, Ren L, Hirsch JP, et al (2005) Structural and functional analysis of essential pre-mRNA splicing factor Prp19p. Mol Cell Biol 25: 451–460. 15601865 10.1128/MCB.25.1.451-460.2005 PMC538785

[54] Cheng SC, Tarn WY, Tsao TY, Abelson J (1993) PRP19: a novel spliceosomal component. Mol Cell Biol 13: 1876–1882. 8441419 10.1128/mcb.13.3.1876-1882.1993 PMC359501

[55] Tarn WY, Lee KR, Cheng SC (1993) The yeast PRP19 protein is not tightly associated with small nuclear RNAs, but appears to associate with the spliceosome after binding of U2 to the pre-mRNA and prior to formation of the functional spliceosome. Mol Cell Biol 13: 1883–1891. 7680101 10.1128/mcb.13.3.1883-1891.1993 PMC359502

[56] Tarn WY, Lee KR, Cheng SC (1993) Yeast precursor mRNA processing protein PRP19 associates with the spliceosome concomitant with or just after dissociation of U4 small nuclear RNA. Proc Natl Acad Sci U S A 90: 10821–10825. 8248176 10.1073/pnas.90.22.10821 PMC47870

[57] Tsai WY, Chow YT, Chen HR, Huang KT, Hong RI, et al (1999) Cef1p is a component of the Prp19p-associated complex and essential for pre-mRNA splicing. J Biol Chem 274: 9455–9462. 10092627 10.1074/jbc.274.14.9455

[58] McDonald WH, Ohi R, Smelkova N, Frendewey D, Gould KL (1999) Myb-related fission yeast cdc5p is a component of a 40S snRNP-containing complex and is essential for pre-mRNA splicing. Mol Cell Biol 19: 5352–5362. 10409726 10.1128/mcb.19.8.5352 PMC84378

[59] Query CC, Konarska MM (2012) *CEF1/CDC5* alleles modulate transitions between catalytic conformations of the spliceosome. Rna 18: 1001–1013. 22408182 10.1261/rna.029421.111 PMC3334688

[60] Pleiss JA, Whitworth GB, Bergkessel M, Guthrie C (2007) Rapid, transcript-specific changes in splicing in response to environmental stress. Mol Cell 27: 928–937. 17889666 10.1016/j.molcel.2007.07.018 PMC2081968

[61] Böhmer C, Böhmer M, Bölker M, Sandrock B (2008) Cdc42 and the Ste20-like kinase Don3 act independently in triggering cytokinesis in *Ustilago maydis* . J Cell Sci 121: 143–148. 18089648 10.1242/jcs.014449

[62] Böhmer C, Ripp C, Bölker M (2009) The germinal centre kinase Don3 triggers the dynamic rearrangement of higher-order septin structures during cytokinesis in *Ustilago maydis* . Mol Microbiol 74: 1484–1496. 19906182 10.1111/j.1365-2958.2009.06948.x

[63] Mahlert M, Leveleki L, Hlubek A, Sandrock B, Bölker M (2006) Rac1 and Cdc42 regulate hyphal growth and cytokinesis in the dimorphic fungus *Ustilago maydis* . Mol Microbiol 59: 567–578. 16390450 10.1111/j.1365-2958.2005.04952.x

[64] Weinzierl G, Leveleki L, Hassel A, Kost G, Wanner G, et al (2002) Regulation of cell separation in the dimorphic fungus *Ustilago maydis* . Mol Microbiol 45: 219–231. 12100561 10.1046/j.1365-2958.2002.03010.x

[65] Freitag J, Lanver D, Böhmer C, Schink KO, Bölker M, et al (2011) Septation of infectious hyphae is critical for appressoria formation and virulence in the smut fungus *Ustilago maydis* . PLoS Pathog 7: e1002044. 21625538 10.1371/journal.ppat.1002044 PMC3098242

[66] Bankmann M, Prakash L, Prakash S (1992) Yeast RAD14 and human xeroderma pigmentosum group A DNA-repair genes encode homologous proteins. Nature 355: 555–558. 1741034 10.1038/355555a0

[67] Mahajan KN, Mitchell BS (2003) Role of human Pso4 in mammalian DNA repair and association with terminal deoxynucleotidyl transferase. Proc Natl Acad Sci U S A 100: 10746–10751. 12960389 10.1073/pnas.1631060100 PMC196874

[68] Ajuh P, Kuster B, Panov K, Zomerdijk JC, Mann M, et al (2000) Functional analysis of the human CDC5L complex and identification of its components by mass spectrometry. EMBO J 19: 6569–6581. 11101529 10.1093/emboj/19.23.6569 PMC305846

[69] Zhang N, Kaur R, Akhter S, Legerski RJ (2009) Cdc5L interacts with ATR and is required for the S-phase cell-cycle checkpoint. EMBO Rep 10: 1029–1035. 19633697 10.1038/embor.2009.122 PMC2750050

[70] Perez-Martin J (2009) DNA-damage response in the basidiomycete fungus *Ustilago maydis* relies in a sole Chk1-like kinase. DNA Repair 8: 720–731. 19269260 10.1016/j.dnarep.2009.01.023

[71] Perez-Martin J, de Sena-Tomas C (2011) Dikaryotic cell cycle in the phytopathogenic fungus *Ustilago maydis* is controlled by the DNA damage response cascade. Plant Signal Behav 6: 1574–1577. 21918381 10.4161/psb.6.10.17055 PMC3256387

[72] Chorev M, Carmel L (2012) The function of introns. Frontiers in genetics 3: 55. 22518112 10.3389/fgene.2012.00055 PMC3325483

[73] Le Hir H, Nott A, Moore MJ (2003) How introns influence and enhance eukaryotic gene expression. Trends Biochem Sci 28: 215–220. 12713906 10.1016/S0968-0004(03)00052-5

[74] Muresan V, Muresan Z (2012) Unconventional functions of microtubule motors. Arch Biochem Biophys 520: 17–29. 22306515 10.1016/j.abb.2011.12.029 PMC3307959

[75] Sun F, Zhu C, Dixit R, Cavalli V (2011) Sunday Driver/JIP3 binds kinesin heavy chain directly and enhances its motility. EMBO J 30: 3416–3429. 21750526 10.1038/emboj.2011.229 PMC3160654

[76] Steinberg G, Schuster M, Theisen U, Kilaru S, Forge A, et al (2012) Motor-driven motility of fungal nuclear pores organizes chromosomes and fosters nucleocytoplasmic transport. J Cell Biol 198: 343–355. 22851316 10.1083/jcb.201201087 PMC3413351

[77] König J, Baumann S, Koepke J, Pohlmann T, Zarnack K, et al (2009) The fungal RNA-binding protein Rrm4 mediates long-distance transport of *ubi1* and *rho3* mRNAs. EMBO J 28: 1855–1866. 19494833 10.1038/emboj.2009.145 PMC2711182

[78] Becht P, Vollmeister E, Feldbrügge M (2005) Role for RNA-binding proteins implicated in pathogenic development of *Ustilago maydis* . Eukaryot Cell 4: 121–133. 15643068 10.1128/EC.4.1.121-133.2005 PMC544158

[79] Zimyanin VL, Belaya K, Pecreaux J, Gilchrist MJ, Clark A, et al (2008) *In vivo* imaging of *oskar* mRNA transport reveals the mechanism of posterior localization. Cell 134: 843–853. 18775316 10.1016/j.cell.2008.06.053 PMC2585615

[80] Chang YF, Imam JS, Wilkinson MF (2007) The nonsense-mediated decay RNA surveillance pathway. Annu Rev Biochem 76: 51–74. 17352659 10.1146/annurev.biochem.76.050106.093909

[81] Brendza RP, Serbus LR, Saxton WM, Duffy JB (2002) Posterior localization of dynein and dorsal-ventral axis formation depend on kinesin in *Drosophila* oocytes. Curr Biol 12: 1541–1545. 12225672 10.1016/s0960-9822(02)01108-9 PMC3209760

[82] Chanarat S, Seizl M, Strasser K (2011) The Prp19 complex is a novel transcription elongation factor required for TREX occupancy at transcribed genes. Genes Dev 25: 1147–1158. 21576257 10.1101/gad.623411 PMC3110953

[83] Katahira J, Yoneda Y (2009) Roles of the TREX complex in nuclear export of mRNA. RNA Biol 6: 149–152. 19229134 10.4161/rna.6.2.8046

[84] Katahira J, Strasser K, Podtelejnikov A, Mann M, Jung JU, et al (1999) The Mex67p-mediated nuclear mRNA export pathway is conserved from yeast to human. EMBO J 18: 2593–2609. 10228171 10.1093/emboj/18.9.2593 PMC1171339

[85] Santos-Rosa H, Moreno H, Simos G, Segref A, Fahrenkrog B, et al (1998) Nuclear mRNA export requires complex formation between Mex67p and Mtr2p at the nuclear pores. Mol Cell Biol 18: 6826–6838. 9774696 10.1128/mcb.18.11.6826 PMC109266

[86] Segref A, Sharma K, Doye V, Hellwig A, Huber J, et al (1997) Mex67p, a novel factor for nuclear mRNA export, binds to both poly(A)+ RNA and nuclear pores. EMBO J 16: 3256–3271. 9214641 10.1093/emboj/16.11.3256 PMC1169942

[87] Sambrook J, Frisch EF, Maniatis T (1989) Molecular Cloning: A Laboratory Manual. Cold Spring Harbour, New York: Cold Spring Harbour Laboratory Press.

[88] Tsukuda T, Carleton S, Fotheringham S, Holloman WK (1988) Isolation and characterization of an autonomously replicating sequence from *Ustilago maydis* . Mol Cell Biol 8: 3703–3709. 2851726 10.1128/mcb.8.9.3703-3709.1988 PMC365426

[89] Holliday R (1974) *Ustilago maydis*. In: King RC, editor. Handbook of Genetics. New York, USA: Plenum Press. pp. 575–595.

[90] Mahlert M, Vogler C, Stelter K, Hause G, Basse CW (2009) The *a2* mating-type-locus gene *lga2* of *Ustilago maydis* interferes with mitochondrial dynamics and fusion, partially in dependence on a Dnm1-like fission component. J Cell Sci 122: 2402–2412. 19531588 10.1242/jcs.039354

[91] Müller P, Weinzierl G, Brachmann A, Feldbrügge M, Kahmann R (2003) Mating and pathogenic development of the Smut fungus *Ustilago maydis* are regulated by one mitogen-activated protein kinase cascade. Eukaryot Cell 2: 1187–1199. 14665454 10.1128/EC.2.6.1187-1199.2003 PMC326639

[92] Pontecorvo G, Roper JA, Hemmons LM, Macdonald KD, Bufton AW (1953) The genetics of *Aspergillus nidulans* . Adv Genet 5: 141–238. 13040135 10.1016/s0065-2660(08)60408-3

[93] Hill TWuK, E (2001) Improved protocols for *Aspergillus* minimal medium: trace element and minimal medium salt stock solutions. Fungal Genetics Newsletter 48: 20–21.

[94] Gillissen B, Bergemann J, Sandmann C, Schröer B, Bölker M, et al (1992) A two-component regulatory system for self/non-self recognition in *Ustilago maydis* . Cell 68: 647–657. 1739973 10.1016/0092-8674(92)90141-x

[95] Mendoza-Mendoza A, Eskova A, Weise C, Czajkowski R, Kahmann R (2009) Hap2 regulates the pheromone response transcription factor prf1 in *Ustilago maydis* . Mol Microbiol 72: 683–698. 19400774 10.1111/j.1365-2958.2009.06676.x

[96] Hoffman CS, Winston F (1987) A ten-minute DNA preparation from yeast efficiently releases autonomous plasmids for transformation of *E. coli* . Gene 57: 267–272. 3319781 10.1016/0378-1119(87)90131-4

[97] Schulz B, Banuett F, Dahl M, Schlesinger R, Schäfer W, et al (1990) The *b* alleles of *U. maydis*, whose combinations program pathogenic development, code for polypeptides containing a homeodomain-related motif. Cell 60: 295–306. 1967554 10.1016/0092-8674(90)90744-y

[98] Timberlake WE, Marshall MA (1989) Genetic engineering of filamentous fungi. Science 244: 1313–1317. 2525275 10.1126/science.2525275

[99] Yelton MM, Hamer JE, Timberlake WE (1984) Transformation of *Aspergillus nidulans* by using a *trpC* plasmid. Proc Natl Acad Sci U S A 81: 1470–1474. 6324193 10.1073/pnas.81.5.1470 PMC344858

[100] Kämper J (2004) A PCR-based system for highly efficient generation of gene replacement mutants in *Ustilago maydis* . Mol Genet Genomics 271: 103–110. 14673645 10.1007/s00438-003-0962-8

[101] Brachmann A, König J, Julius C, Feldbrügge M (2004) A reverse genetic approach for generating gene replacement mutants in *Ustilago maydis* . Mol Genet Genomics 272: 216–226. 15316769 10.1007/s00438-004-1047-z

[102] Scherer M, Heimel K, Starke V, Kämper J (2006) The Clp1 protein is required for clamp formation and pathogenic development of *Ustilago maydis* . Plant Cell 18: 2388–2401. 16920779 10.1105/tpc.106.043521 PMC1560919

[103] Heimel K, Scherer M, Schuler D, Kämper J (2010) The *Ustilago maydis* Clp1 protein orchestrates pheromone and *b*-dependent signaling pathways to coordinate the cell cycle and pathogenic development. Plant Cell 22: 2908–2922. 20729384 10.1105/tpc.110.076265 PMC2947178

[104] Garrido E, Perez-Martin J (2003) The *crk1* gene encodes an Ime2-related protein that is required for morphogenesis in the plant pathogen *Ustilago maydis* . Mol Microbiol 47: 729–743. 12535072 10.1046/j.1365-2958.2003.03323.x

[105] Trapnell C, Pachter L, Salzberg SL (2009) TopHat: discovering splice junctions with RNA-Seq. Bioinformatics 25: 1105–1111. 19289445 10.1093/bioinformatics/btp120 PMC2672628

[106] Anders S, Huber W (2010) Differential expression analysis for sequence count data. Genome Biol 11: R106. 20979621 10.1186/gb-2010-11-10-r106 PMC3218662

[107] Hardcastle TJ, Kelly KA (2010) baySeq: empirical Bayesian methods for identifying differential expression in sequence count data. BMC Bioinformatics 11: 422. 20698981 10.1186/1471-2105-11-422 PMC2928208

[108] Lanver D, Mendoza-Mendoza A, Brachmann A, Kahmann R (2010) Sho1 and Msb2-related proteins regulate appressorium development in the smut fungus Ustilago maydis. Plant Cell 22: 2085–2101. 20587773 10.1105/tpc.109.073734 PMC2910971

[109] Abramoff MD, Magalhaes PJ, Ram SJ (2004) Image Processing with Image J. Biophotonics International 11: 36–42.

[110] Katoh K, Toh H (2008) Recent developments in the MAFFT multiple sequence alignment program. Brief Bioinform 9: 286–298. 18372315 10.1093/bib/bbn013

