# Supplementary material for: The SPF27 Homologue Num1 Connects Splicing and Kinesin 1-Dependent Cytoplasmic Trafficking in Ustilago maydis
Source: PLoS Genet. 2014 Jan 2;10(1):e1004046. doi: 10.1371/journal.pgen.1004046 (PMC3879195; doi:10.1371/journal.pgen.1004046)
Supplement: Figure S1 — Alignment of Num1 to SPF27 homologues. ClustalW was used to align proteins related to Num1 from S. reilianum (Sr12752, NCBI accession number CBQ71896), U. hordei (UHOR_02497, CCF53376) C. cinerea (Num1, BAC78620), A. nidulans (AN4244, XP_661848.1), A. thaliana (Mos4, AT3G18165.1), mouse (SPF27, BAB31409) and human (BCAS2/SPF27, CAG46834). Identical or similar residues are highlighted in black and grey, respectively. The nuclear localization signal (NLS) in the U. maydis sequence is highlighted in green. The red box indicates the conserved BCAS2-domain. (PDF) [file pgen.1004046.s001.pdf]

Figure_S1

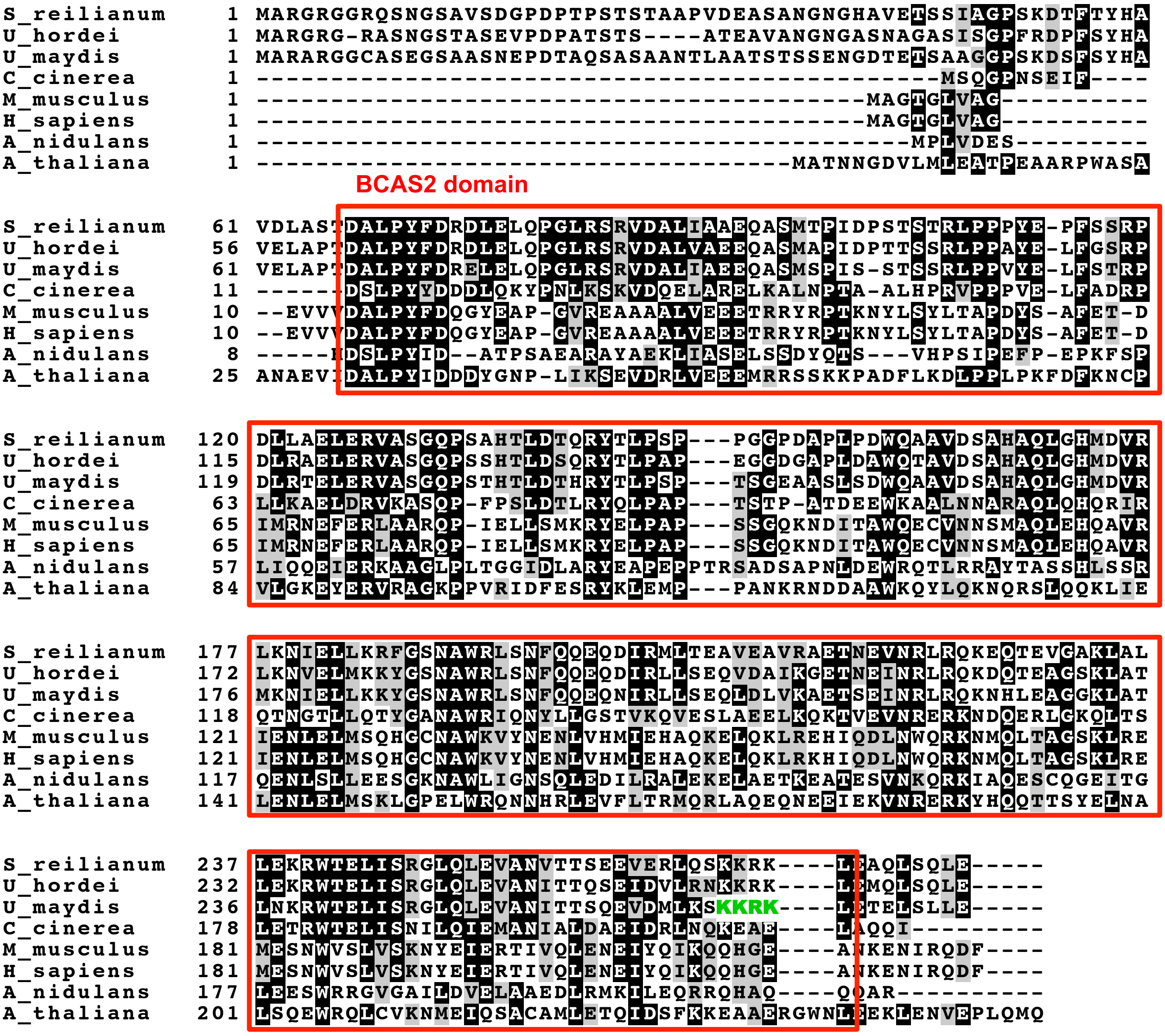

S_reilianum 1 MARGRGGRQSNGSAVSDGPDPTPSTSTAAPVDEASANGNGHAVETSSIAGPSKDTFTYHA
U_hordei 1 MARGRG-RASNGSTASEVPDPATSTS----ATEAVANGNGASNAGASISGPFRDPFSYHA
U_maydis 1 MARARGGCASEGSAASNEPDTAQSASAANTLAATSTSSENGDTETSAAGGPSKDSFSYHA
C_cinerea 1 --MSQGPNSEIF----
M_musculus 1 ---MAGTGLVAG----------
H_sapiens 1 ---MAGTGLVAG----------
A_nidulans 1 --MPLVDES---------
A_thaliana 1 ------------------------------------MATNNGDVLMLEATPEAARPWASA
BCAS2 domain
S_reilianum 61 VDLASTDALPYFDRDLELQPGLRSRVDALIAAEQASMTPIDPSTSTRLPPPYE-PFSSRP
U_hordei 56 VELAPTDALPYFDRDLELQPGLRSRVDALVAEEQASMAPIDPTTSSRLPPAYE-LFGSRP
U_maydis 61 VELAPTDALPYFDRELELQPGLRSRVDALIAEEQASMSPIS-STSSRLPPVYE-LFSTRP
C_cinerea 11 ------DSLPYYDDDLQKYPNLKSKVDQELARELKALNPTA-ALHPRVPPPVE-LFADRP
M_musculus 10 --EVVVDALPYFDQGYEAP-GVREAAAALVEEETRRYRPTKNYLSYLTAPDYS-AFET-D
H_sapiens 10 --EVVVDALPYFDQGYEAP-GVREAAAALVEEETRRYRPTKNYLSYLTAPDYS-AFET-D
A_nidulans 8 -----HDSLPYID--ATPSAEARAYAEKLIASELSSDYQTS---VHPSIPEFP-EPKFSP
A_thaliana 25 ANAEVIDALPYIDDDYGNP-LIKSEVDRLVEEEMRRSSKKPADFLKDLPPLPKFDFKNCP
S_reilianum 120 DLLAELERVASGQPSAHTLDTQRYTLPSP---PGGPDAPLPDWQAAVDSAHAQLGHMDVR
U_hordei 115 DLRAELERVASGQPSSHTLDSQRYTLPAP---EGGDGAPLDAWQTAVDSAHAQLGHMDVR
U_maydis 119 DLRTELERVASGQPSTHTLDTHRYTLPSP---TSGEAASLSDWQAAVDSAHAQLGHMDVR
C_cinerea 63 LLKAELDRVKASQP-FPSLDTLRYQLPAP---TSTP-ATDEEWKAALNNARAQLQHQRIR
M_musculus 65 IMRNEFERLAARQP-IELLSMKRYELPAP---SSGQKNDITAWQECVNNSMAQLEHQAVR
H_sapiens 65 IMRNEFERLAARQP-IELLSMKRYELPAP---SSGQKNDITAWQECVNNSMAQLEHQAVR
A_nidulans 57 LIQQEIERKAAGLPLTGGIDLARYEAPEPPTRSADSAPNLDEWRQTLRRAYTASSHLSSR
A_thaliana 84 VLGKEYERVRAGKPPVRIDFESRYKLEMP---PANKRNDDAAWKQYLQKNQRSLQQKLIE
S_reilianum 177 LKNIELLKRFGSNAWRLSNFQQEQDIRMLTEAVEAVRAETNEVNRLRQKEQTEVGAKLAL
U_hordei 172 LKNVELMKKYGSNAWRLSNFQQEQDIRLLSEQVDAIKGETNEINRLRQKDQTEAGSKLAT
U_maydis 176 MKNIELLKKYGSNAWRLSNFQQEQNIRLLSEQLDLVKAETSEINRLRQKNHLEAGGKLAT
C_cinerea 118 QTNGTLLQTYGANAWRIQNYLLGSTVKQVESLAEELKQKTVEVNRERKNDQERLGKQLTS
M_musculus 121 IENLELMSQHGCNAWKVYNENLVHMIEHAQKELQKLREHIQDLNWQRKNMQLTAGSKLRE
H_sapiens 121 IENLELMSQHGCNAWKVYNENLVHMIEHAQKELQKLRKHIQDLNWQRKNMQLTAGSKLRE
A_nidulans 117 QENLSLLEESGKNAWLIGNSQLEDILRALEKELAETKEATESVNKQRKIAQESCQGEITG
A_thaliana 141 LENLELMSKLGPELWRQNNHRLEVFLTRMQRLAQEQNEEIEKVNRERKYHQQTTSYELNA
S_reilianum 237 LEKRWTELISRGLQLEVANVTTSEEVERLQSKKRK----LEAQLSQLE-----
U_hordei 232 LEKRWTELISRGLQLEVANITTQSEIDVLRNKKRK----LEMQLSQLE-----
U_maydis 236 LNKRWTELISRGLQLEVANITTSQEVDMLKSKKRK----LETELSLLE-----
C_cinerea 178 LETRWTELISNILQIEMANIALDAEIDRLNQKEAE----LAQQI---------
M_musculus 181 MESNWVSLVSKNYEIERTIVQLENEIYQIKQQHGE----ANKENIRQDF----
H_sapiens 181 MESNWVSLVSKNYEIERTIVQLENEIYQIKQQHGE----ANKENIRQDF----
A_nidulans 177 LEESWRRGVGAILDVELAAEDLRMKILEQRRQHAQ----QQAR----------
A_thaliana 201 LSQEWRQLCVKNMEIQSACAMLETQIDSFKKEAAERGWNLEEKLENVEPLQMQ
