# Supplementary figures and images for: The SPF27 Homologue Num1 Connects Splicing and Kinesin 1-Dependent Cytoplasmic Trafficking in Ustilago maydis
Source: PLoS Genet. 2014 Jan 2;10(1):e1004046. doi: 10.1371/journal.pgen.1004046 (PMC3879195; doi:10.1371/journal.pgen.1004046)

Figure\_S2

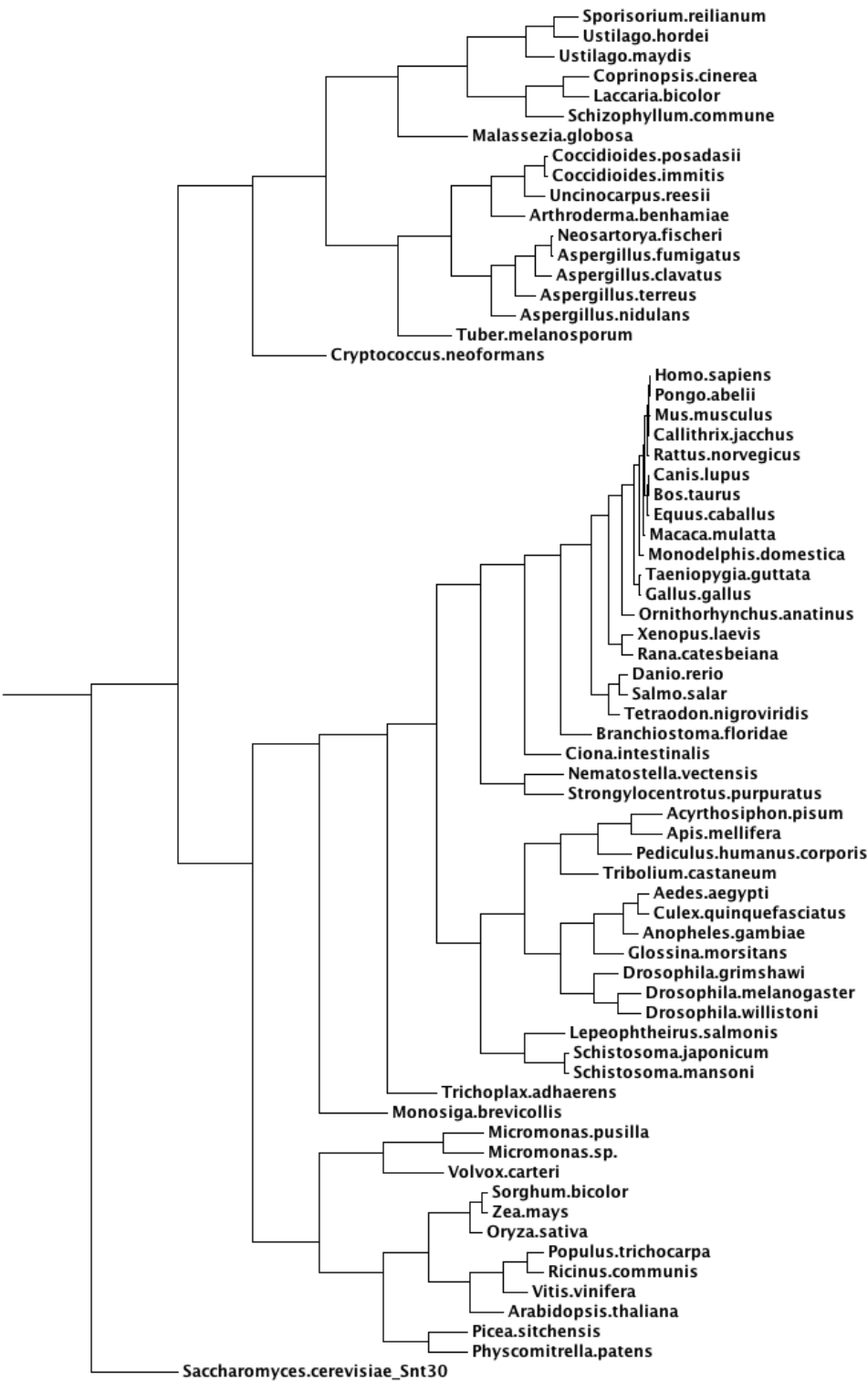

Supplement: Figure S2 — Phylogeny of Num1 homologues. For comparative phylogenetic analysis of Num1, 65 sequences with the highest similarity to Num1 were obtained by BLASTP analysis. The sequence of Saccharomyces cerevisiae Snt309p was included as out-group. Sequences were aligned with MAFFT version 6 [110] using the global alignment G-INS-i. A phylogenetic tree was calculated using the minimum linkage clustering method. (PDF) [file pgen.1004046.s002.pdf]

Figure\_S3

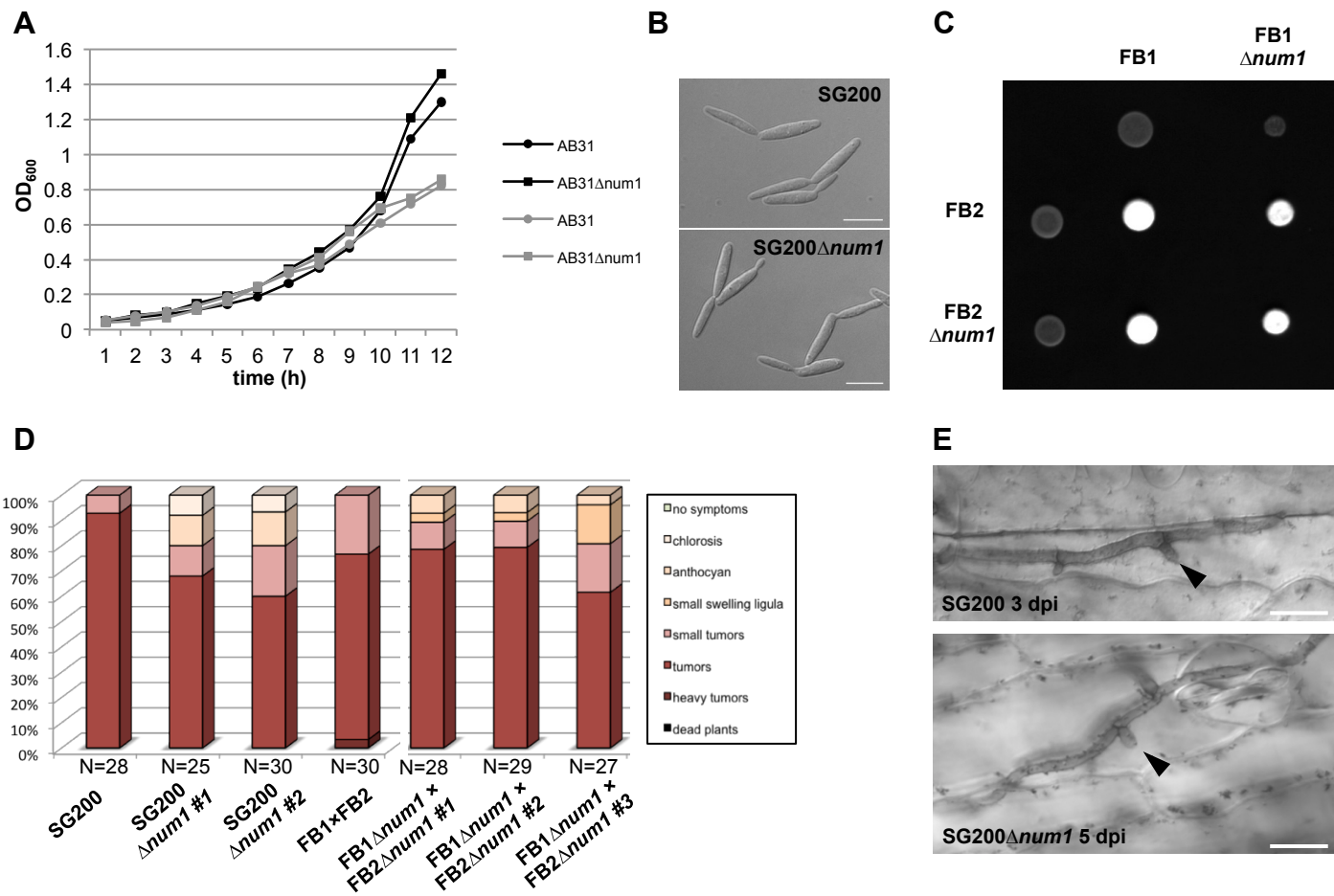

Supplement: Figure S3 — Deletion of num1 does not affect growth in axenic culture, mating and dikaryon formation or proliferation in planta. (A) Growth curves of AB31 and AB31Δnum1 deletion strain in YEPSL complex- (black) and glutamin/glucose-containing minimal medium (grey). The num1-deletion has no influence on growth in axenic culture. (B) SG200 (control) and SG200Δnum1 sporidia were grown in glucose-containing CM-medium. No obvious phenotype could be observed, indicating that the Δnum1-mutant is not impaired during growth in axenic culture. (C) Mating assays on charcoal-containing CM-glucose medium by co-spotting of strains indicated. FB1 (a1b1) and FB2 (a2b2) strains are included as wild-type controls. Formation of dikaryotic aerial hyphae is visible as white mycelium. The num1-deletion has no influence on the formation of the dikaryon. (D) Pathogenicity of individual num1-deletion strains. Disease rating of maize seedlings seven days post inoculation with U. maydis strains SG200, SG200Δnum1, FB1×FB2 (wild-type crosses) and FB1Δnum1×FB2Δnum1-derivatives. #1, #2 indicate independently obtained deletion mutants. Bars represent the percentage of infected plants with the symptoms indicated in the legend. N corresponds to the total number of plants infected. (E) Maize plants were inoculated with SG200 and SG200Δnum1. Infected leaves were stained with Chlorazole Black E. Samples were taken three and five days post inoculation, respectively. Clamp-like structures (arrowheads) could be observed in both strains, indicating fungal proliferation within the plant cells. Scale bars: 10 µm. (PDF) [file pgen.1004046.s003.pdf]

Figure\_S4

FB1 + pheromone

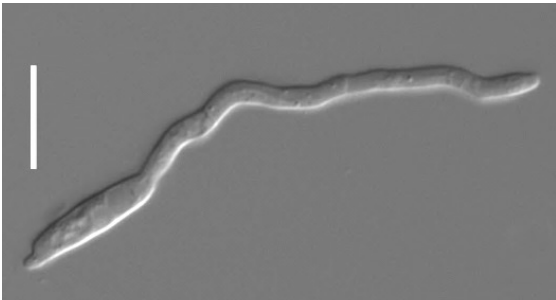

FB1 $\Delta$ num1 + pheromone

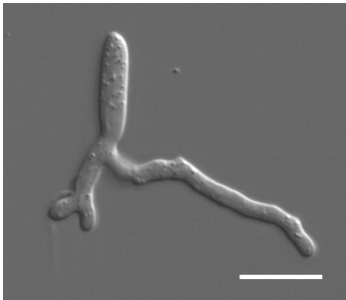

FB1 + DMSO

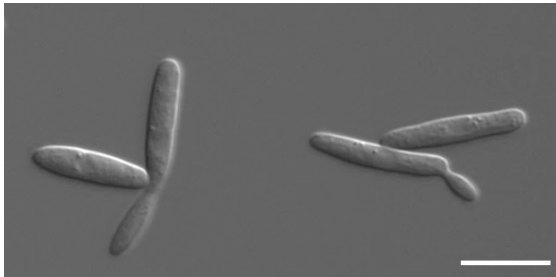

FB1 $\Delta$ num1 + DMSO

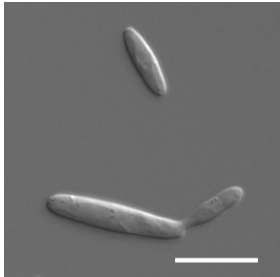

Supplement: Figure S4 — Phenotype of conjugation hyphae of num1-deletion strains.. Strains FB1 and FB1Δnum1 were treated with synthetic a2-pheromone (2,5 µg/ml) for six hours in glucose-containing CM-medium. DMSO was used as a solvent for the a2-pheromone and served as negative control. Both FB1 and FB1Δnum1 respond to the pheromone with the formation of conjugation tubes. Note the characteristic Δnum1-mutant phenotype, such as branches and irregular hyphal growth. Scale bars: 10 µm. (PDF) [file pgen.1004046.s004.pdf]

Figure\_S5

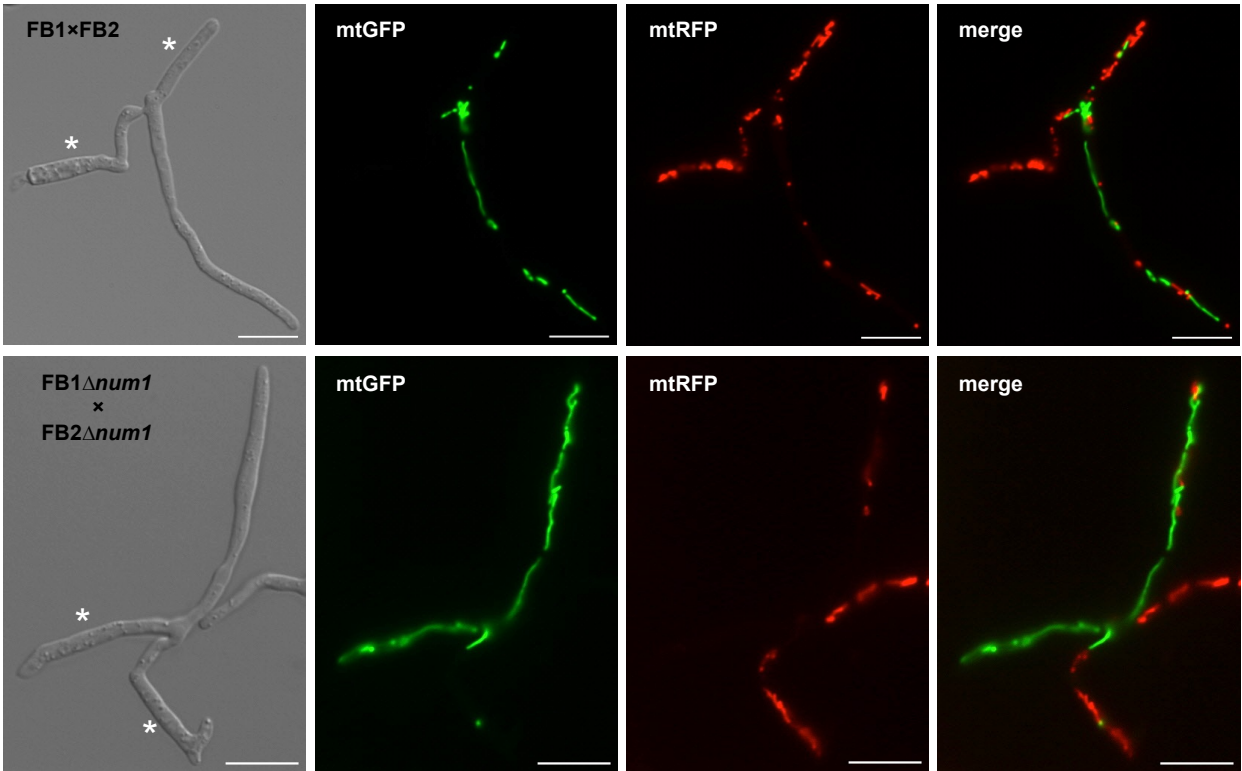

Supplement: Figure S5 — Phenotype of dikaryotic hyphae of num1-deletion strains. Compatible FB1- and FB2-strains were used to monitor the Δnum1-phenotype in dikaryotic hyphae. To demonstrate successful fusion events, FB1-strains express mitochondrial matrix-targeted RFP (mtRFP), and FB2-strains express mitochondrial matrix-targeted GFP (mtGFP), respectively, under control of the inducible Pcrg1-promoter. Fusion of the strains was induced in arabinose-containing liquid charcoal medium overnight. Depicted are DIC-, GFP- and RFP-signals. Merged images show both fluorescent signals within the same hypha, indicating fusion of two sporidia in compatible FB1×FB2 wild-type crossings (upper panel) as well as in compatible FB1Δnum1×FB2Δnum1 crossings (lower panel). The two initial cells are marked with an asterisk. Note the characteristic Δnum1-mutant phenotype (thicker, irregular hyphal growth and bipolar growth of the sporidia). Scale bars: 10 µm. (PDF) [file pgen.1004046.s005.pdf]

Figure\_S6

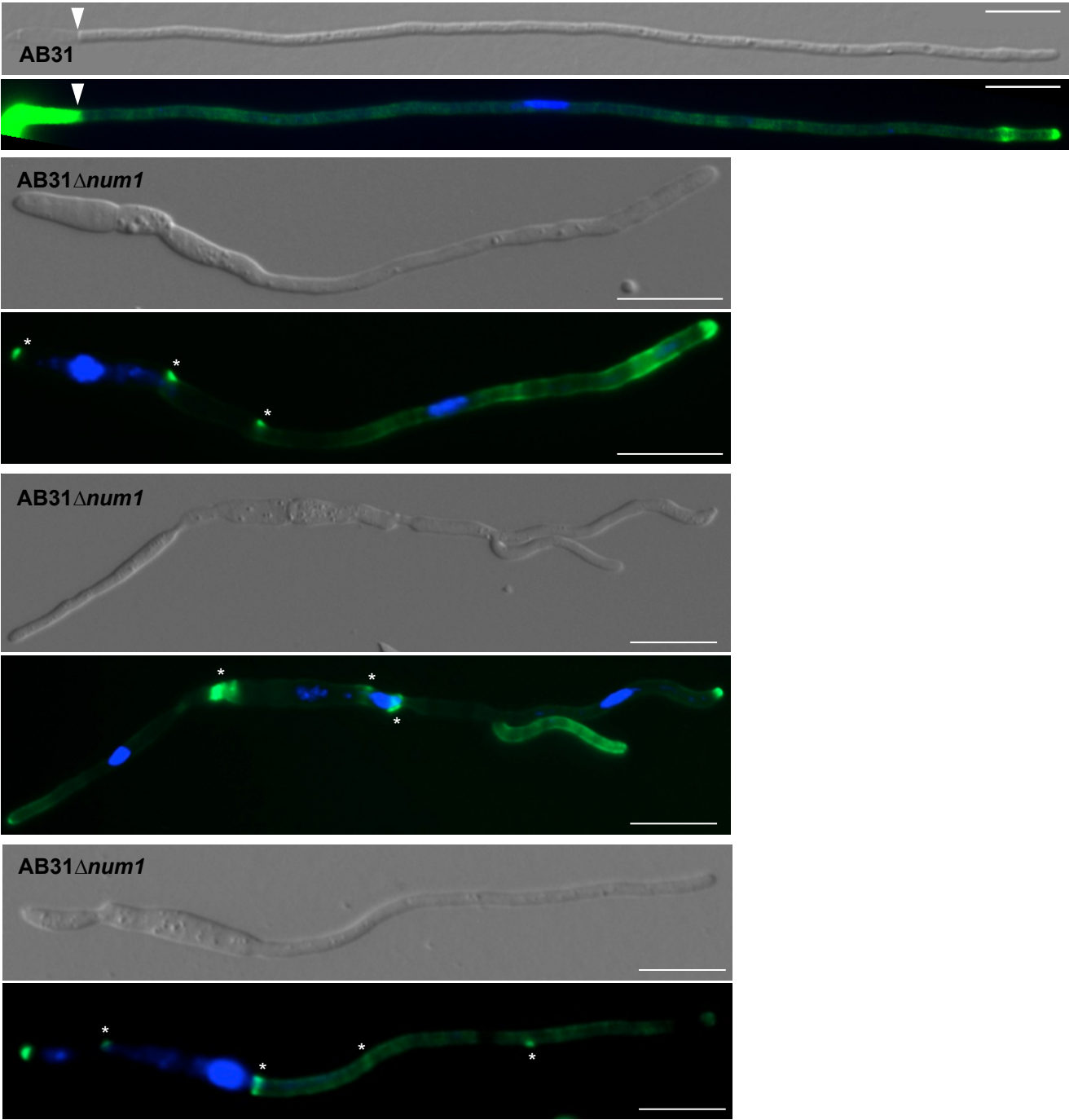

Supplement: Figure S6 — The Δnum1-deletion causes chitin accumulations and the insertion of unusual septa. Induction of the bE1/bW2 heterodimer in wild-type cells (AB31) induces a G2 cell cycle arrest, resulting in hyphae with a single nucleus positioned in the tip compartment. 18% of AB31Δnum1 mutant hyphae contain more than one nucleus within the cell. Filaments were stained with DAPI to visualize nuclei and Fluorescein-conjugated wheat germ agglutinin (WGA), which specifically binds to N-acetylglucosamine [27]. In AB31 wild-type cells, chitin was intensively stained in the growth region within the hyphal apex as well as in the basal retraction septum (marked with arrowhead). In AB31Δnum1 mutants, however, chitin accumulations along the hyphae (marked by asterisks) as well as septa-like chitin rings were observed frequently (middle and lower panel). In many cases, these septa were not visible in the DIC channel, indicating that these structures may not represent true septal cell walls. Scale bars: 10 µm. Septa in AB31Δnum1 were also visualized by Calcofluor (Figures 1E and 6B) and Congo Red (Figure 3A). In contrast to septa in the dikaryotic hyphae, septa in AB31Δnum1 were not always visible in the DIC channel, indicating that these septa or septa-like structures may be structurally different from wild-type septa. (PDF) [file pgen.1004046.s006.pdf]

Figure\_S7

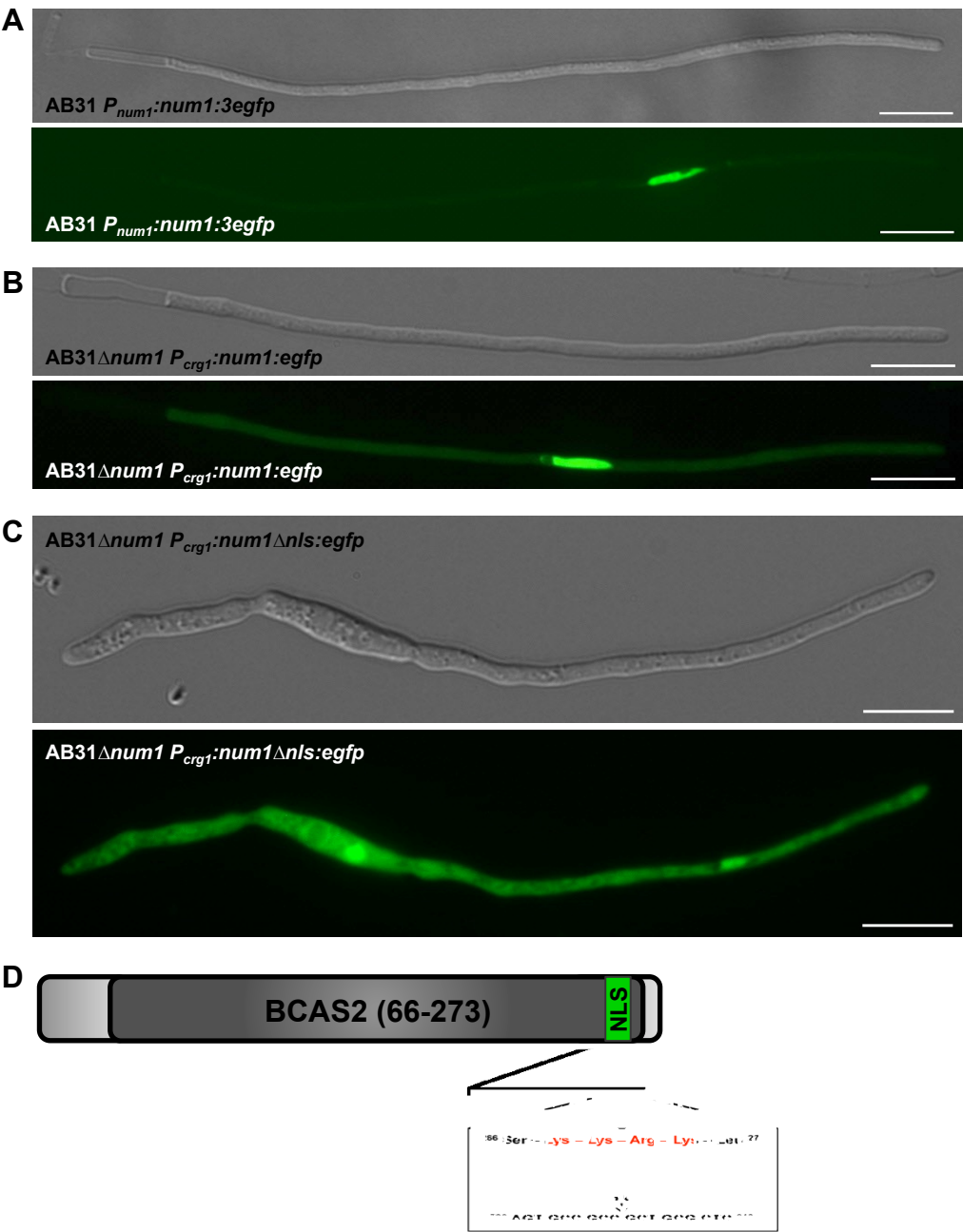

Supplement: Figure S7 — Complementation analyses of num1-deletion strains. Filaments of AB31 and AB31Δnum1, expressing Num1:eGFP fusion proteins under control of the native Pnum1- or the inducible Pcrg1-promoter, were analyzed 12–14 hours after induction of the bE1/bW2-heterodimer and, when present, the Num1:eGFP fusion proteins. (A) In AB31, Num1:eGFP fusion proteins localize predominantly to the nucleus. (B) Complementation of the num1-deletion with a Num1:eGFP fusion protein restores the wild-type phenotype and the protein localizes predominantly to the nucleus. (C) A Num1:3eGFP fusion protein with mutated nuclear localization signal (NLS, shown in D) does not complement the Δnum1 phenotype; filaments show the typical curved and bipolar morphology of the num1-deletion. In addition to the GFP-signal in the nucleus, a strong signal is observed in the cytoplasm. Scale bars: 10 µm. (D) Schematic representation of the Num1 protein depicting the basic amino acids within the NLS that were replaced by alanine-residues. (PDF) [file pgen.1004046.s007.pdf]

Figure\_S8

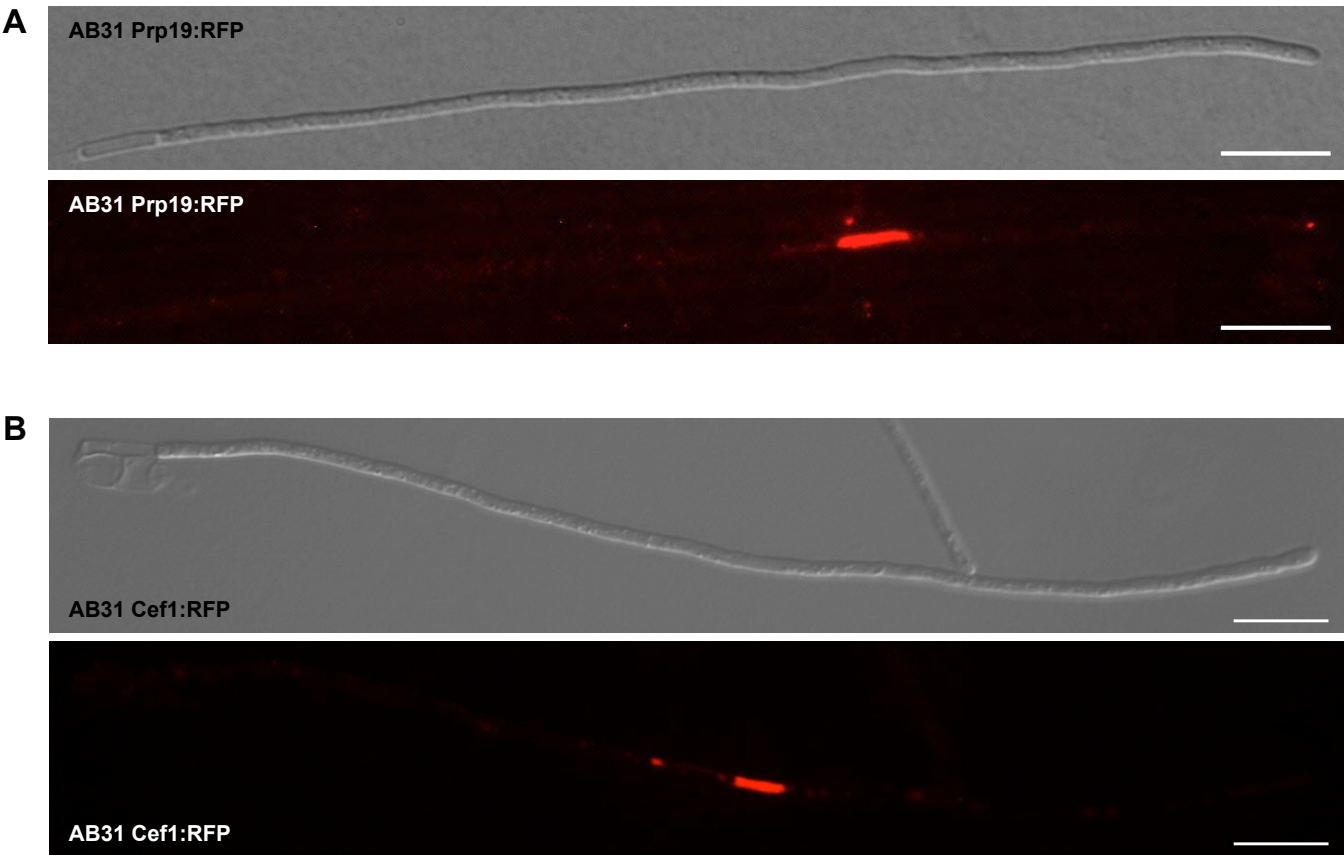

Supplement: Figure S8 — Prp19- and Cef1-fusion proteins are functional and are localized in the nucleus. prp19 and cef1 were expressed as C-terminal RFP- or HA-tagged versions under control of their endogenous promoters in strain AB31 to ensure native expression levels. Hyphal growth was analyzed 14 hours after induction of the bE1/bW2-heterodimer. (A) Shown are DIC- and RFP-fluorescence signals of strains UNK208 (AB31 num1:3egfp:hygR, prp19:rfp:natR) and (B) UMO10 (AB31 num1:3egfp:hygR, cef1:rfp:natR). As prp19 and cef1 are both essential genes (see Supporting Text S1), the viability of the strains proofs the functionality of the fusion proteins. In addition, no altered phenotypes with respect to filamentous growth were observed, corroborating that both Prp19:RFP as well as Cef1:RFP fusion proteins are functional. Scale bars: 10 µm. (PDF) [file pgen.1004046.s008.pdf]

Figure\_S9

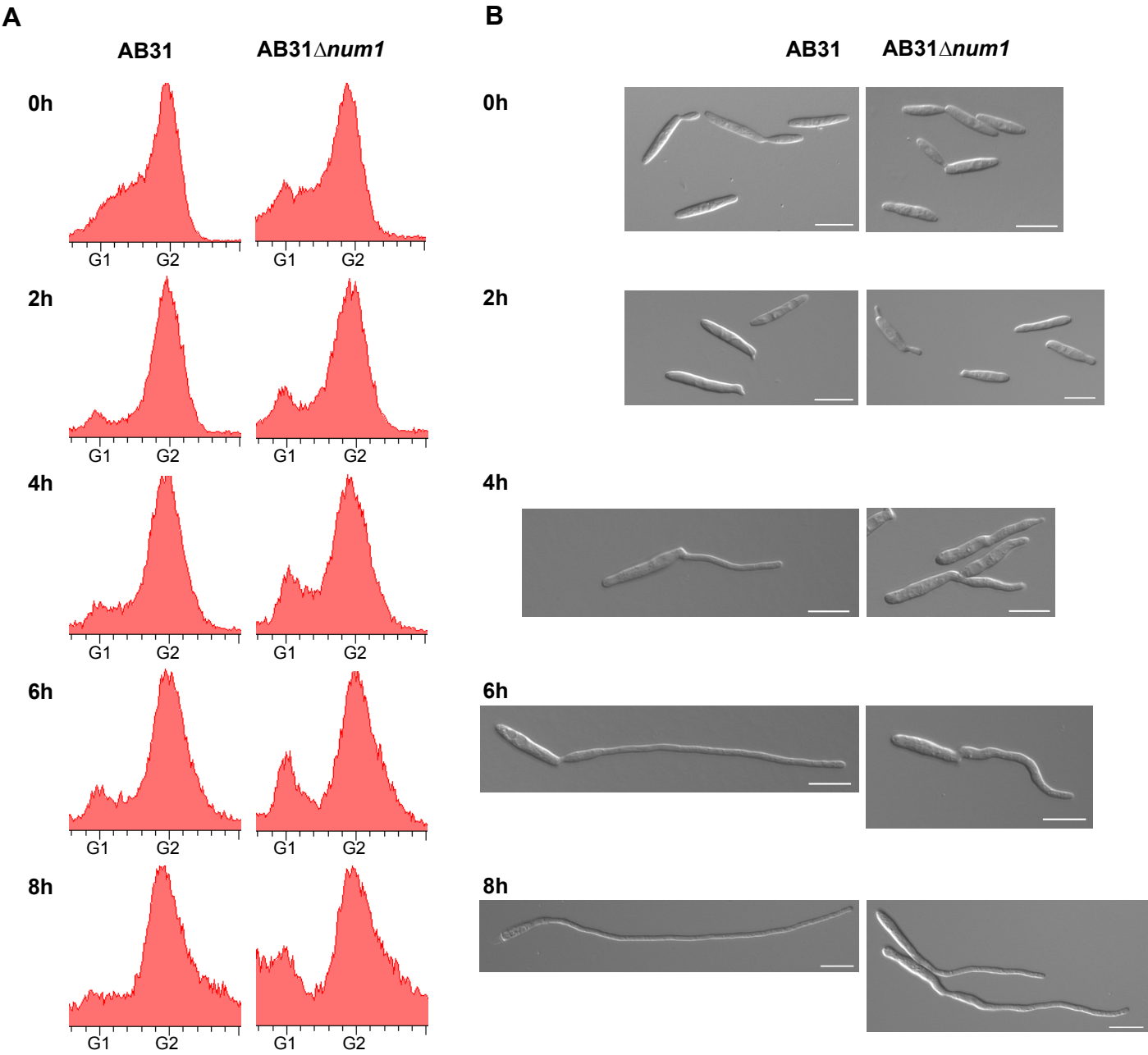

Supplement: Figure S9 — Cell cycle analysis of AB31 vs. AB31Δnum1. (A) FACS analysis of AB31 and AB31Δnum1 after induction of the bE1/bW2-heterodimer in arabinose-containing CM-medium. Samples were taken at time points indicated. The histograms show the DNA-content measured by FACS analysis. Relative fluorescence intensities are given on horizontal axes, vertical axes reflect cell numbers. In AB31, induction of the active bE1/bW2-combination resulted in an enrichment of cells with a 2C DNA-content, indicative of a G2 cell cycle arrest. In AB31Δnum1, a higher amount of cells in G1 was observed, which implicates defects in cell cycle regulation. (B) Microscopic analysis of the filamentous growth of the strains upon b-induction. Scale bars: 10 µm. (PDF) [file pgen.1004046.s009.pdf]

Figure\_S10

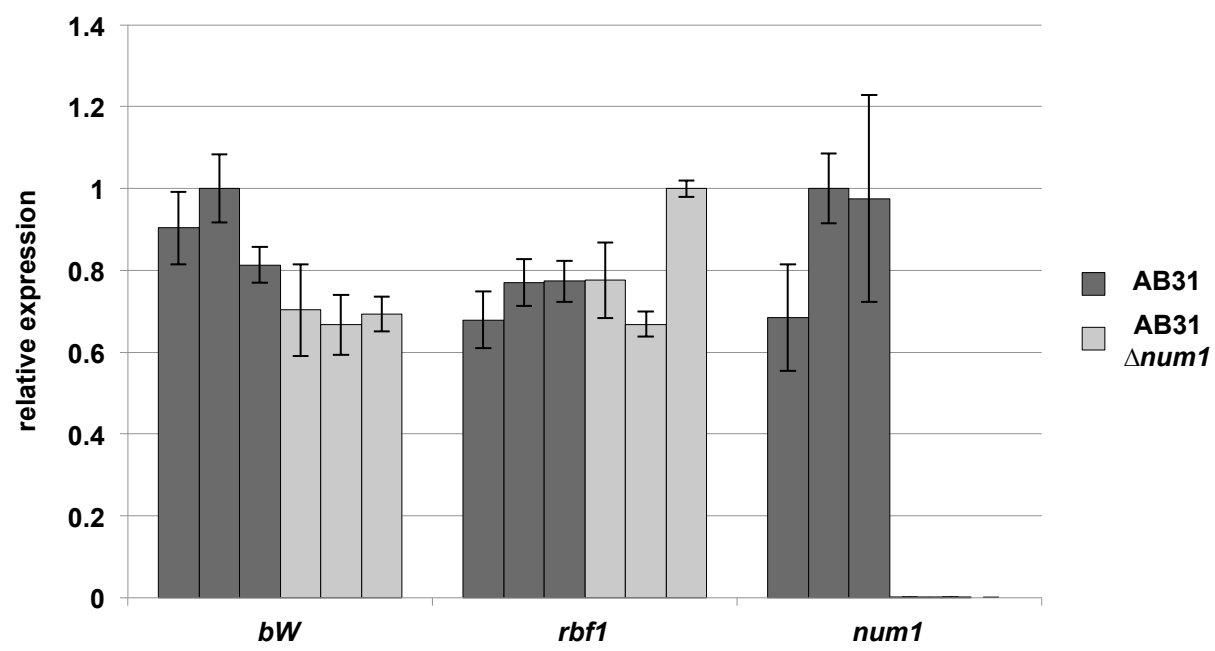

Supplement: Figure S10 — Control of bW-, rbf1- and num1-gene expression in samples used for RNA-Seq-analysis. RNA was isolated from strains AB31 and AB31Δnum1 in three biological replicates each, eight hours after induction of the bE1/bW2-heterodimer in arabinose-containing minimal medium. Expression of bW-, rbf1- and num1 was monitored by qRT-PCR analysis. Gene expression is shown relative to the highest expression value. Actin and eIF2b were used for normalization. Shown are the mean values two technical replicates. Error bars represent the SD. (PDF) [file pgen.1004046.s010.pdf]

Figure\_S11

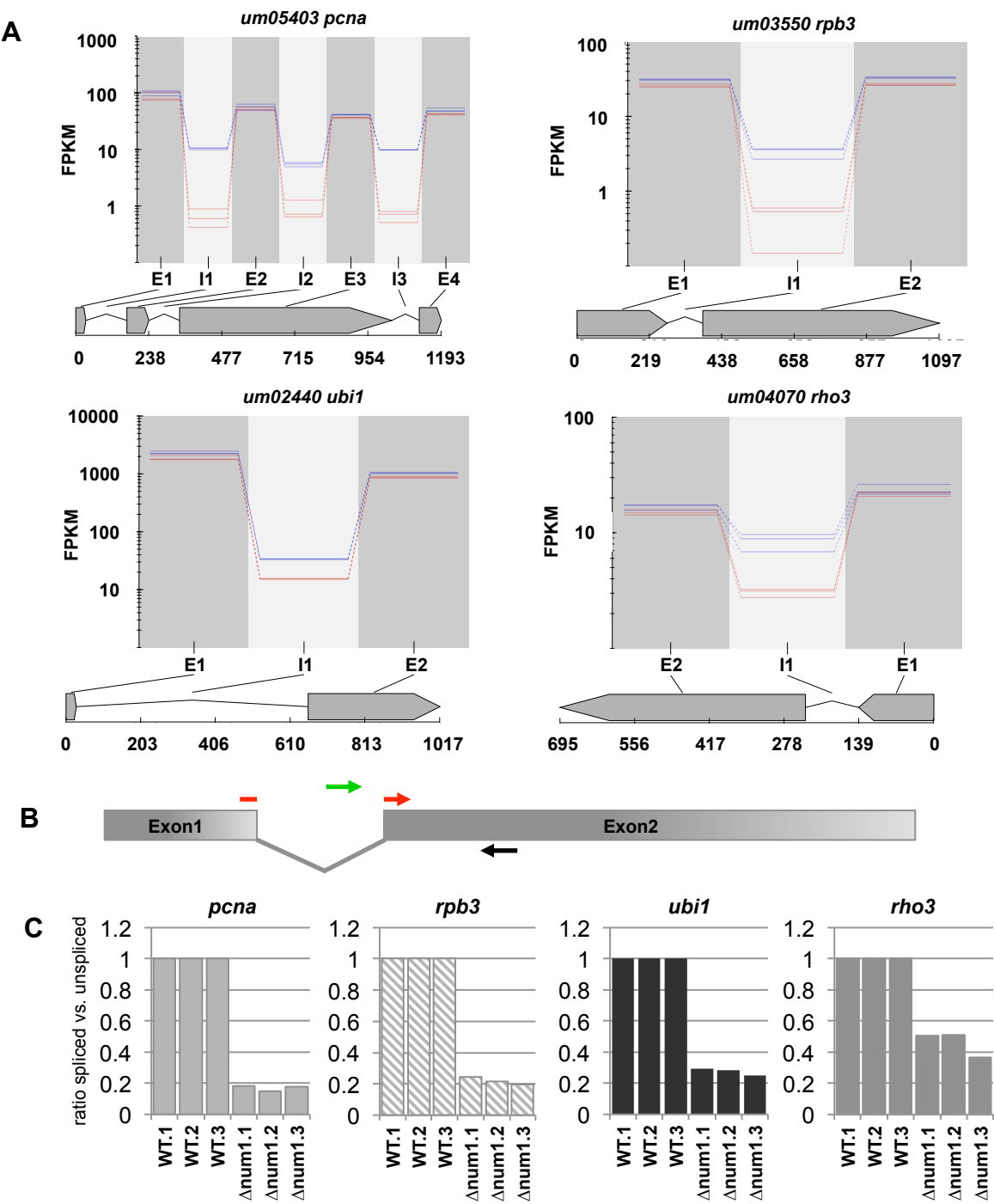

Supplement: Figure S11 — Verification of mRNA-Seq using qRT-PCR. (A) Plot depicting splicing efficiency of genes based on the RNA-Seq analysis. Plotted are the FPKM values (fragments per kilobase of sequence per million fragments mapped) across the genomic region indicated (coordinates in nucleotides) of three independent RNA-Seq experiments for AB31 wild-type (blue lines) and AB31Δnum1 (red lines), respectively. Exons (E) and introns (I) are indicated. (B) Schematic representation of exon-intron structures. Oligonucleotides used for qRT-PCR are depicted as arrows and allow the discrimination against spliced (primer depicted in red) and unspliced transcripts (primer depicted in green). (C) Gene expression analyses to verify expression of spliced vs. unspliced transcripts of the indicated genes using qRT-PCR. RNA was isolated from AB31 and AB31Δnum1 eight hours after induction of the bE/bW-heterodimer. Actin and eIF2b were used for normalization. Depicted are the ratios of spliced vs. unspliced transcripts of three independent biological replicates. Mean values of two technical replicates each are shown. (PDF) [file pgen.1004046.s011.pdf]

Figure\_S13

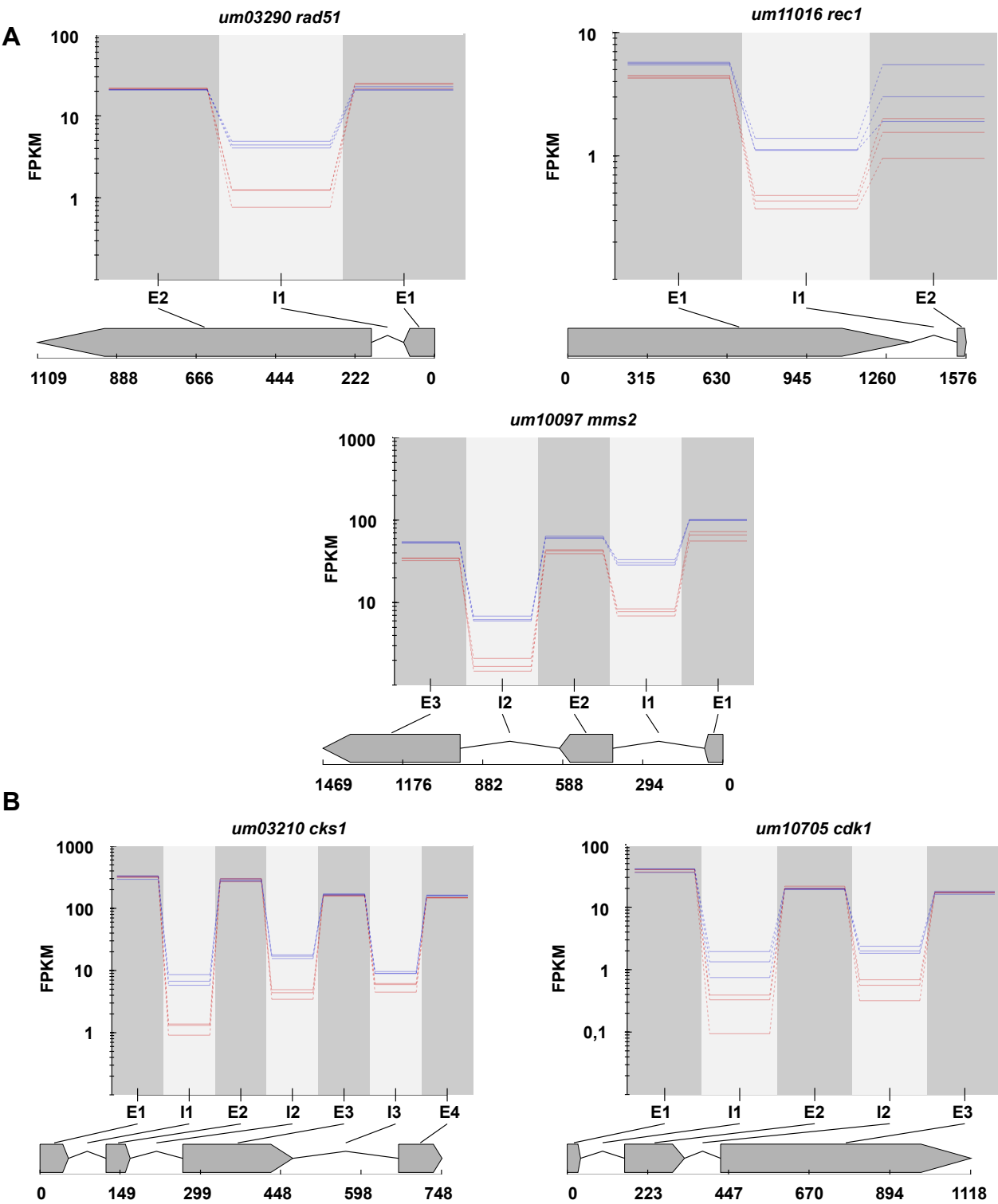

Supplement: Figure S13 — Reduced splicing efficiency in AB31Δnum1 in genes with function in DNA repair and cell cycle. Splicing efficiency based on RNA-Seq analysis. Depicted are examples for genes grouping in the functional category (A) “DNA repair” and (B) “cell cycle”. Plotted are the FPKM values (fragments per kilobase of sequence per million fragments mapped) across the genomic region indicated (coordinates in nucleotides) of three independent RNA-Seq experiments for AB31 wild-type (blue lines) and AB31Δnum1 (red lines), respectively. Exons (E) and introns (I) are indicated. (PDF) [file pgen.1004046.s013.pdf]

Figure\_S14

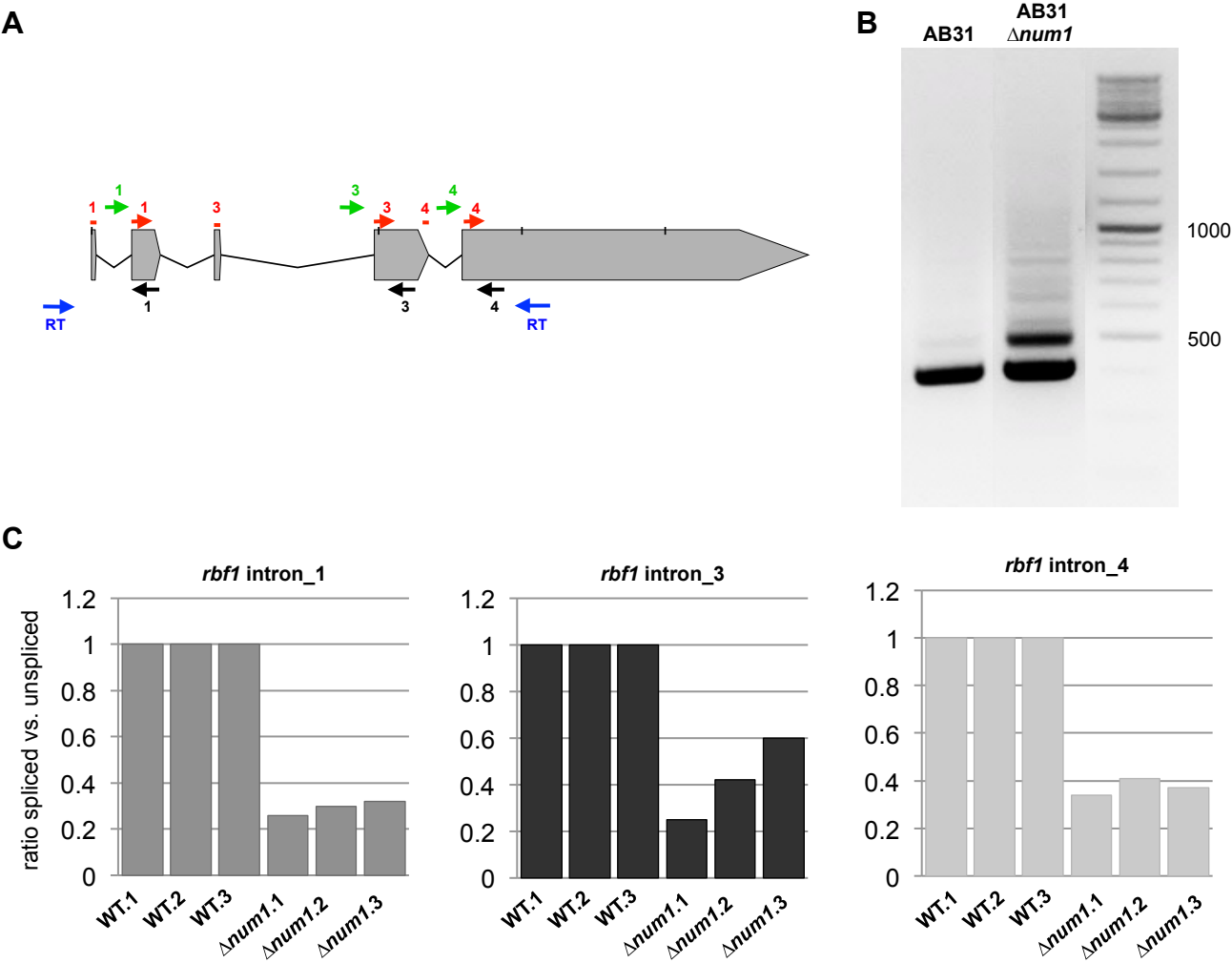

Supplement: Figure S14 — Splicing defect of the rbf1-gene in AB31Δnum1. (A) Schematic view of rbf1 intron/exon structure and primers used for RT-PCR (blue) and qRT-PCR (red, green and black). (B) RNA samples of AB31 and AB31Δnum1 were isolated eight hours after induction of the bE1/bW2-heterodimer. RT-PCR analysis was performed on the rbf1 open reading frame and PCR products were separated on a 2% TAE-agarose gel. In AB31 cDNA, the spliced version of rbf1 (430 bp) is predominantly detected. In AB31Δnum1 more bands spanning different sizes from 430 bp (all four introns spliced) to 1192 bp (all introns retained) are observed, validating the splicing defect of the rbf1-gene observed in the RNA-Seq analysis. (C) qRT-PCR analysis to investigate the expression of spliced vs. unspliced rbf1-transcripts. Primers depicted in (A) were used to distinguish spliced (primer depicted in red) from non-spliced (primer depicted in green) introns. Actin and eIF2b were used for normalization. Depicted are the ratios of spliced vs. unspliced transcripts of three independent biological replicates. Mean values of two technical replicates each are shown. (PDF) [file pgen.1004046.s014.pdf]

Figure\_S15

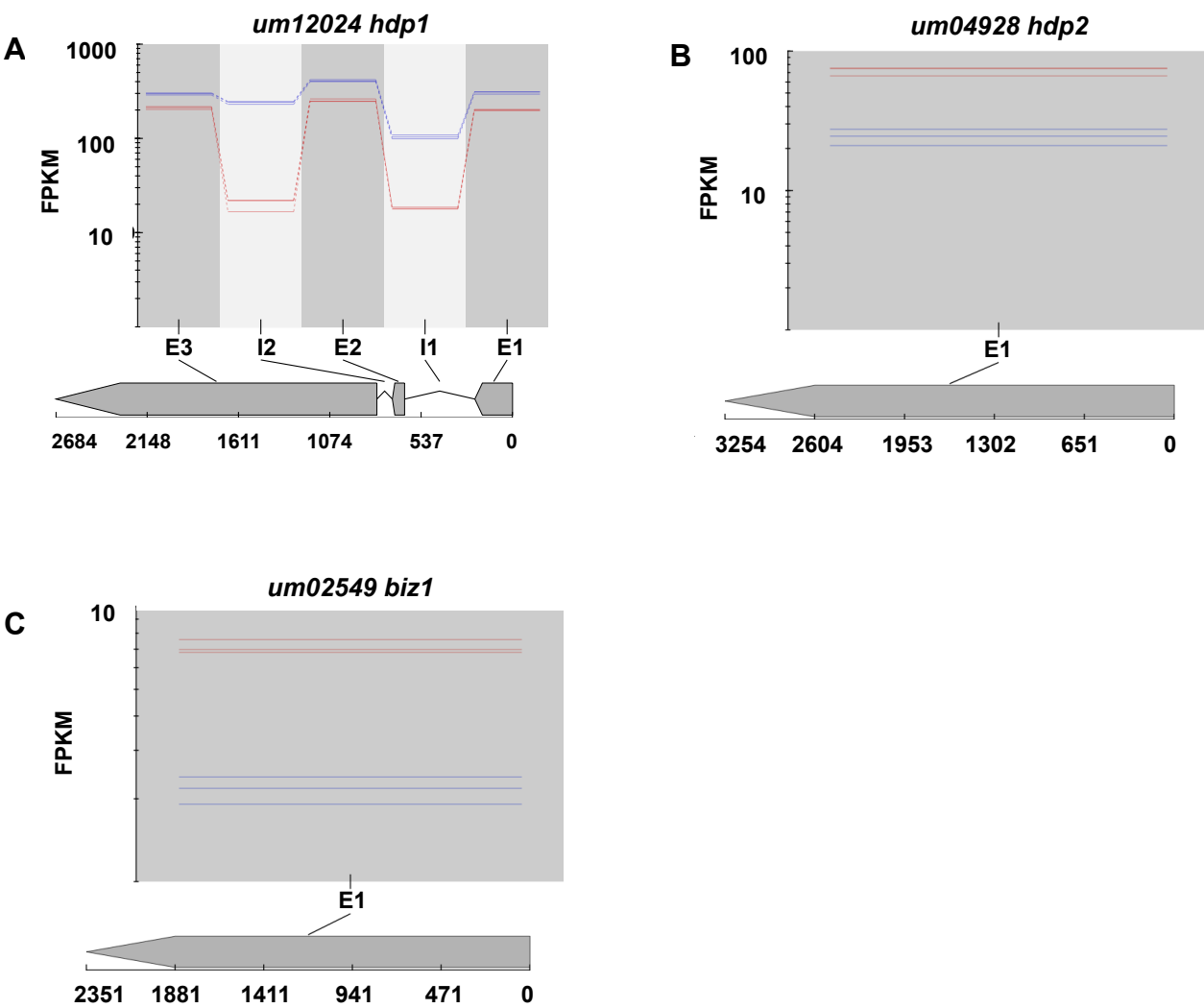

Supplement: Figure S15 — Splicing efficiency and expression profile of the Rbf1-target genes hdp1, hdp2, biz1 is altered in AB31Δnum1. Shown are the expression profiles based on the RNA-Seq analysis. Plotted are the FPKM values (fragments per kilobase of sequence per million fragments mapped) across the genomic region indicated (coordinates in nucleotides) of three independent RNA-Seq experiments for AB31 wild-type (blue lines) and AB31Δnum1 (red lines), respectively. Exons (E) and introns (I) are indicated. (A) Expression of the hdp1-gene is increased about two-fold, and splicing efficiency of both introns is reduced in AB31Δnum1. (B) and (C) Expression levels of hdp2 as well as biz1 are significantly reduced in AB31Δnum1 (hdp2: −3,1-fold, p = 5.28 * 10−36 biz1: −3,4-fold, p = 5.34 * 10−62; significance according to DEseq [106]). (PDF) [file pgen.1004046.s015.pdf]

Figure\_S16

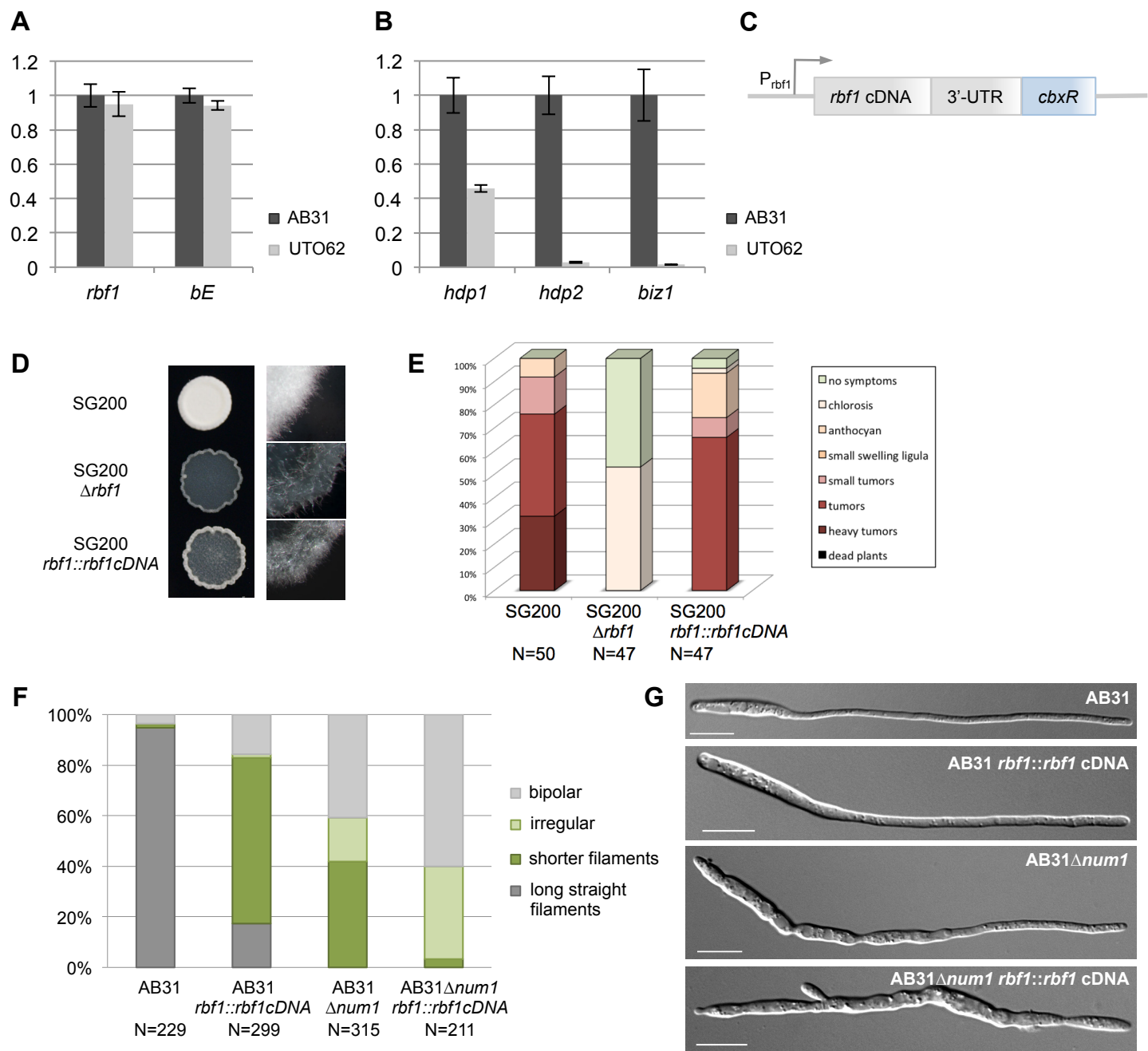

Supplement: Figure S16 — Introns are essential for full function of rbf1. (A) and (B) An intron-free derivative of rbf1 was expressed in AB31, replacing the endogenous rbf1-gene. cDNA was isolated from strains AB31 and UTO62 (AB31 rbf1::rbf1cDNA) 10 hours after induction of filamentous growth in arabinose-containing CM-medium. qRT-PCR analysis was performed to verify unaltered expression of bE and rbf1 (A). The intron-free rbf1-derivative is not able to induce rbf1-target genes (B). Gene expression is shown relative to the highest expression value. Actin and eIF2b were used for normalization. Mean values of two technical replicates each are shown. (C) Schematic representation of the construct used in this study. rbf1-cDNA was expressed in the genomic rbf1-locus under control of the endogenous Prbf1-promoter. 500 bp of the 3′-UTR were included as terminator. A carboxin resistance-cassette was used for selection. (D) SG200-derivatives were spotted on charcoal-containing CM-medium and incubated at 22°C for 3 days. The formation of filamentous hyphae is visible as white mycelium. In contrast to the complementation with a wild-type rbf1-copy [2], the respective intron-free rbf1-derivative, which replaced the endogenous rbf1, was not able to complement the Δrbf1-phenotype. Magnified images of the colony margins were taken with a binocular assisted camera. (E) Maize seedlings were infected with the indicated SG200 derivatives. Disease rating and tumor formation was monitored seven days after infection. Bars represent the percentage of infected plants with symptoms indicated in the legend. N corresponds to the number of plants infected. The intron-free rbf1-derivative leads to a reduction in tumor formation. (F) and (G) Microscopic analysis of the indicated strains 10 hours after induction of filamentous growth in arabinose-containing CM-medium. Phenotypes were grouped into four different categories. Whereas 95% of AB31 wild-type cells grew as long, straight filaments, the intron-free rbf1-derivati [file pgen.1004046.s016.pdf]

Figure\_S17

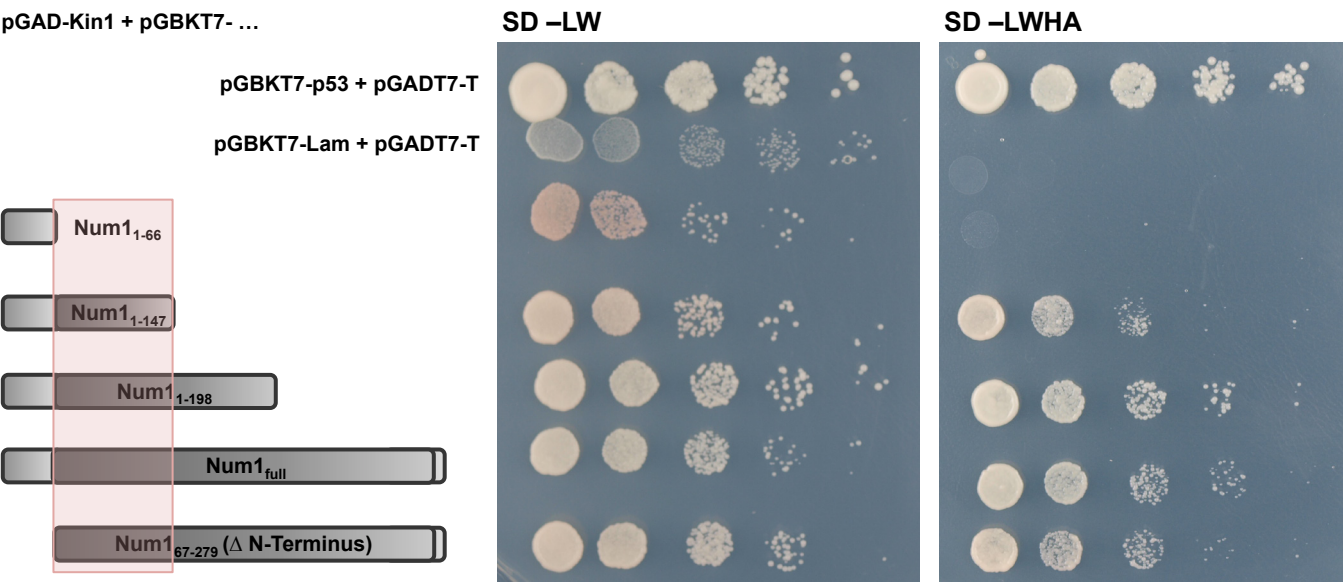

Supplement: Figure S17 — Characterization of the interaction domain of Num1 for the Kin1 motor protein. N- and C-terminally truncated fragments of the Num1-protein were tested for interaction with Kin1 in the yeast two-hybrid system. Left panel: Schematic depiction of the constructs used in this study. Numbers represent the regions of the Num1-constructs in amino acids. The conserved BCAS2-domain is depicted in dark grey. After cloning into pGBKT7 (Clontech), the constructs were co-transformed with the pGAD-Kin1650–968 fragment isolated in the yeast two-hybrid screen. Serial 10-fold dilutions of cell suspensions of the respective transformants were spotted on selective media (SD –LW) and stringent media (SD –LWHA), indicating an interaction. Plates were incubated for two days at 30°C. The Num1 interaction domain resides between amino acids 67 and 147 (highlighted in red). pGBKT7-p53 with pGADT7-T served as positive control, pGBKT7-Lam with pGADT7-T (Clontech) served as negative control. (PDF) [file pgen.1004046.s017.pdf]

Figure\_S18

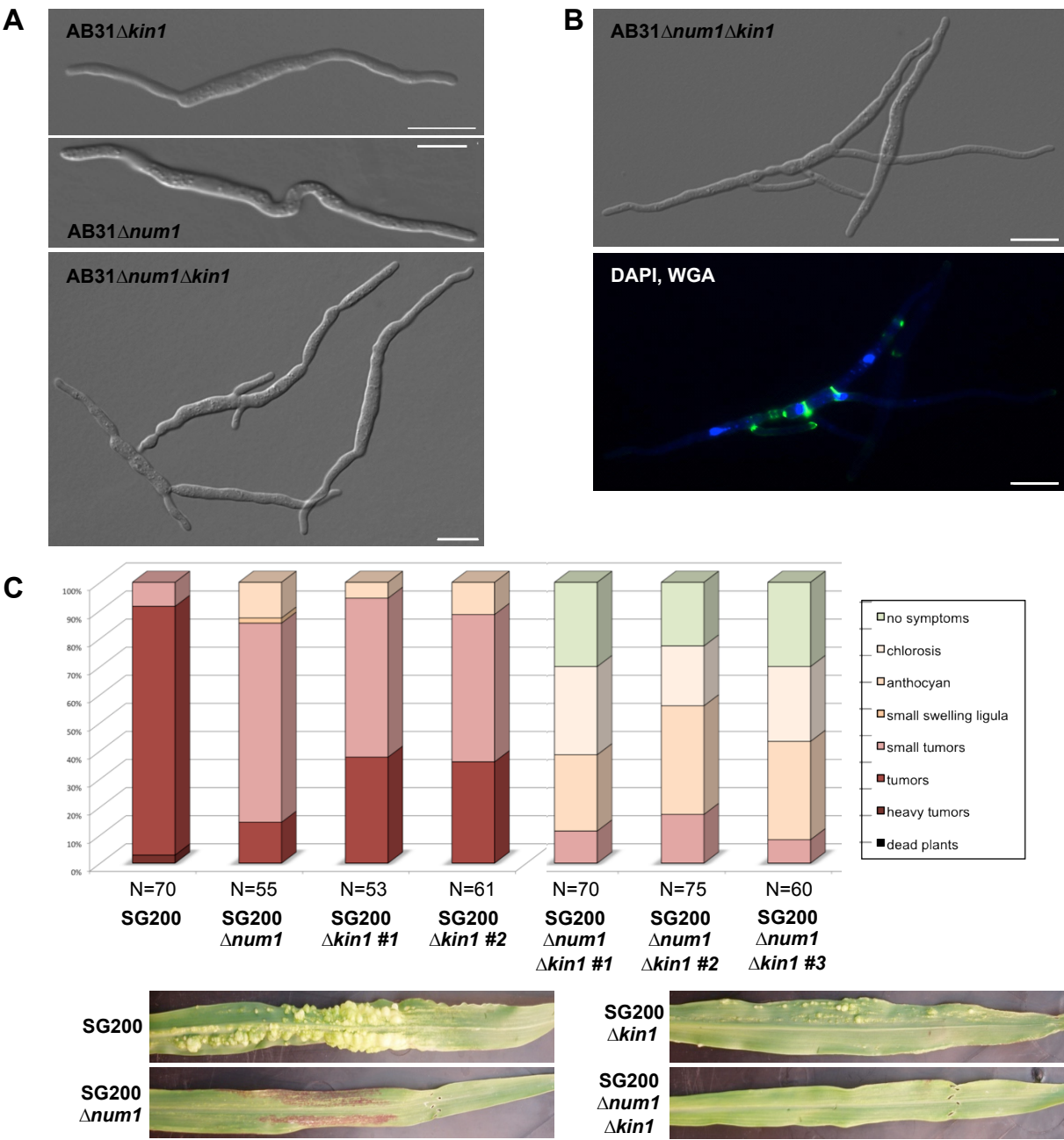

Supplement: Figure S18 — The double deletion of Num1 and Kin1 has additive effects on pathogenicity as well as hyphal morphology. (A) Effects of the double deletion on polar hyphal growth in strain AB31Δnum1Δkin1. Filaments of were analyzed 12 hours after induction of the bE1/bW2-heterodimer. Compared to AB31Δkin1 and AB31Δnum1 hyphae, which predominantly grow as bipolar filaments, the double deletion has additive effects on filament morphology, polarity and branching. (B) To visualize nuclear positioning and septation, filaments of AB31Δnum1Δkin1 were treated with DAPI- and WGA/FITC. Scale bars: 10 µm. (C) Pathogenicity of individual num1- and kin1-deletion strains. Maize seedlings were infected with the indicated SG200 derivatives. #1, #2, #3 indicate independently obtained deletion strains. Disease rating and tumor formation was monitored seven days after inoculation. Bars represent the percentage of infected plants with symptoms indicated in the legend. N corresponds to the total number of plants infected. Deletion of the individual num1 or kin1 genes results in slightly reduced pathogenicity, whereas the num1/kin1-double deletion has a much stronger effect on pathogenicity. Depicted below are disease symptoms (third leaf below the site of injection) of representative maize plants seven days after inoculation. (PDF) [file pgen.1004046.s018.pdf]

Figure\_S19

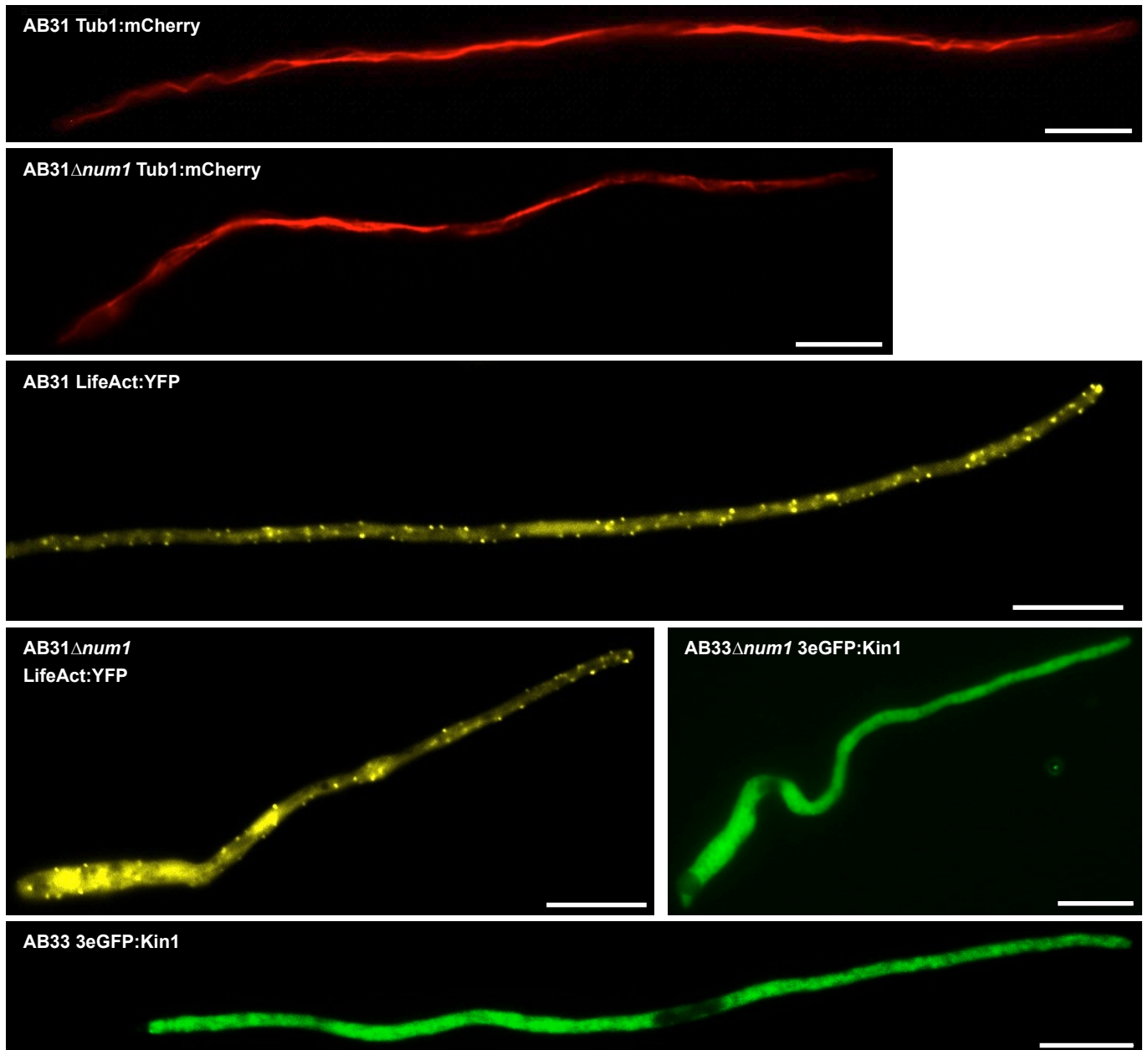

Supplement: Figure S19 — Num1 has neither influence on morphology or abundance of microtubules and actin elements, nor on the distribution of the Kin1 motor protein. Microtubules were analyzed using a Tub1:mCherry fusion protein, the actin cytoskeleton was visualized using the lifeact-method (lifeact:yfp) [44]. Both fusion proteins were expressed under control of the constitutively active Potef-promoter in strains AB31 and AB31Δnum1. For the localization of the Kin1 motor protein in strains AB33 and AB33Δnum1, an N-terminal 3eGFP:Kin1 fusion was used [14]. The fluorescent cytoskeletal elements were microscopically analyzed 12–14 hours after induction of the bE1/bW2-heterodimer in arabinose-containing CM-medium for AB31 and nitrate-containing minimal-medium for AB33, respectively. mCherry, YFP- and GFP-signals are shown. Scale bars: 10 µm. (PDF) [file pgen.1004046.s019.pdf]

Figure\_S20

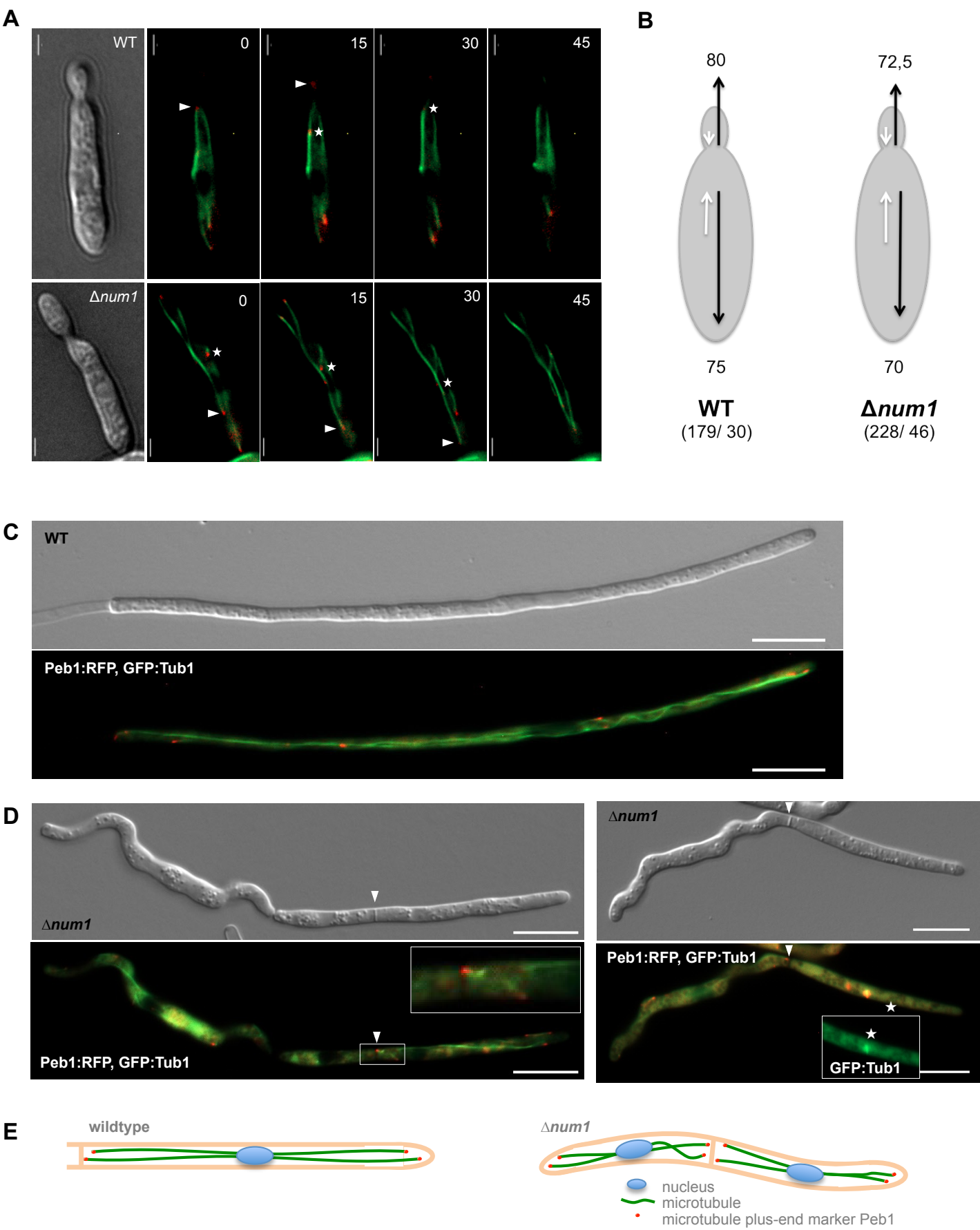

Supplement: Figure S20 — Orientation of the microtubule cytoskeleton in AB33 and AB33Δnum1 sporidia and hyphae. Orientation of the microtubule cytoskeleton was addressed in strain AB33Peb1R_GT (AB33 peb1:mRFP potefGFP:Tub1) [14] and its Δnum1 derivative. In AB33Peb1R_GT wild-type cells, the homologue of the microtubule (MT) plus-end marker EB1, Peb1, fused to RFP, localizes to growing MT plus-ends [45], which are predominantly oriented towards the cell poles of the hyphae [9], [14], or, in interphase cells, towards the budding cell and the opposing cell pole of the mother cell [46]. (A) Motility of Peb1:RFP in budded cells of AB33Peb1R_GT (WT) and its Δnum1 derivative. Peb1:RFP binds to plus-ends of microtubules, and its motility (pictures were taken in 15 sec intervals) indicates the orientation of the microtubule [46]. Arrowheads and asterisks indicate individual Peb1:RFP dots. Scale bar: 2 µm (B) Quantification of microtubule orientation in interphase sporidia. AB33Peb1R_GT (WT) and its Δnum1 derivative were grown in YEPSL medium to mid log phase. Black arrows and numbers above and below the cartoons give the percentage of Peb1-RFP dots moving to either tip of the cell. White arrows show the relative amount of Peb1-RFP to the bud neck. Numbers are based on 45 sec intervals of 30 and 46 cells in wild-type and Δnum1-strains, respectively. The total number of signals used for quantification and the number of cells is given in brackets. Quantification was done according to the protocol given in [46]. (C) In AB33Peb1R_GT wild-type hyphae, Peb1-RFP is predominantly oriented towards the cell poles. (D) In AB33Peb1R_GT Δnum1, Peb1:RFP labeled MT plus-ends are also found at septa (marked by arrowheads), which are often placed in the middle of the hyphae. In some hyphae, mitotic spindles were observed (marked by asterisks), implicating that the num1-deletion leads to a deregulated cell cycle arrest. Peb1-RFP and GFP-Tub1 fluorescence was analyzed 8–10 hours after induction of hyphal growth in nitr [file pgen.1004046.s020.pdf]

Figure\_S21

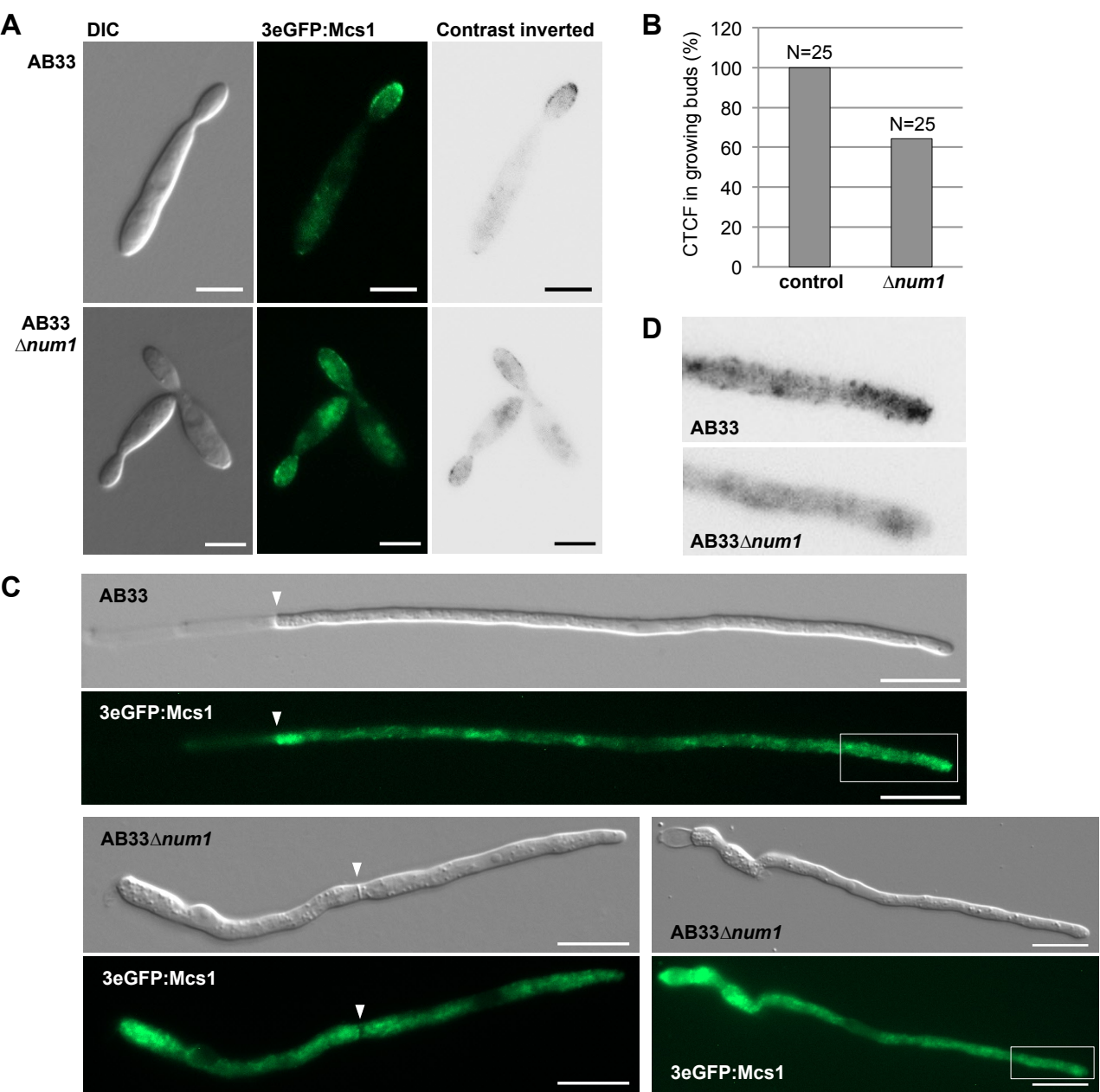

Supplement: Figure S21 — Influence of Num1 on the localization of the myosin chitin synthase Mcs1. (A) In wild-type cells, 3eGFP:Mcs1 localizes to growing bud tips, where it participates in the synthesis of chitin [15]. In contrast, Δnum1-mutants shows reduced Mcs1-accumulation at the growth region. Shown are DIC and GFP-images, as well as contrast-inverted images. Scale bars: 5 µm. (B) Quantitative analysis of the 3eGFP:Mcs1 signal intensity in growing bud cells. The bar diagram shows corrected total cell fluorescence (CTCF) of wild-type (control) and Δnum1-mutant cells. ImageJ software was used for quantification. N corresponds to the number of cells analyzed. (C) In AB33 hyphae, 3eGFP:Mcs1 localizes to distinct foci close to the cell membrane and forms a gradient towards the growth zone within the hyphal apex or localizes to the basal retraction septum (arrowhead), respectively. In contrast, in AB33Δnum1, no tip-ward gradient is obvious and fewer foci are observed at the cell membrane. In many cases Mcs1 does not localize to delocalized septa in the Δnum1-mutant (lower left panel, arrowhead indicates a septum). Mcs1-fluorescence was analyzed 12 hours after induction of hyphal growth in nitrate-containing minimal medium. Scale bars: 10 µm. (D) Enlarged images of the sections marked in (C). Images were contrast-inverted. (PDF) [file pgen.1004046.s021.pdf]

Figure\_S22

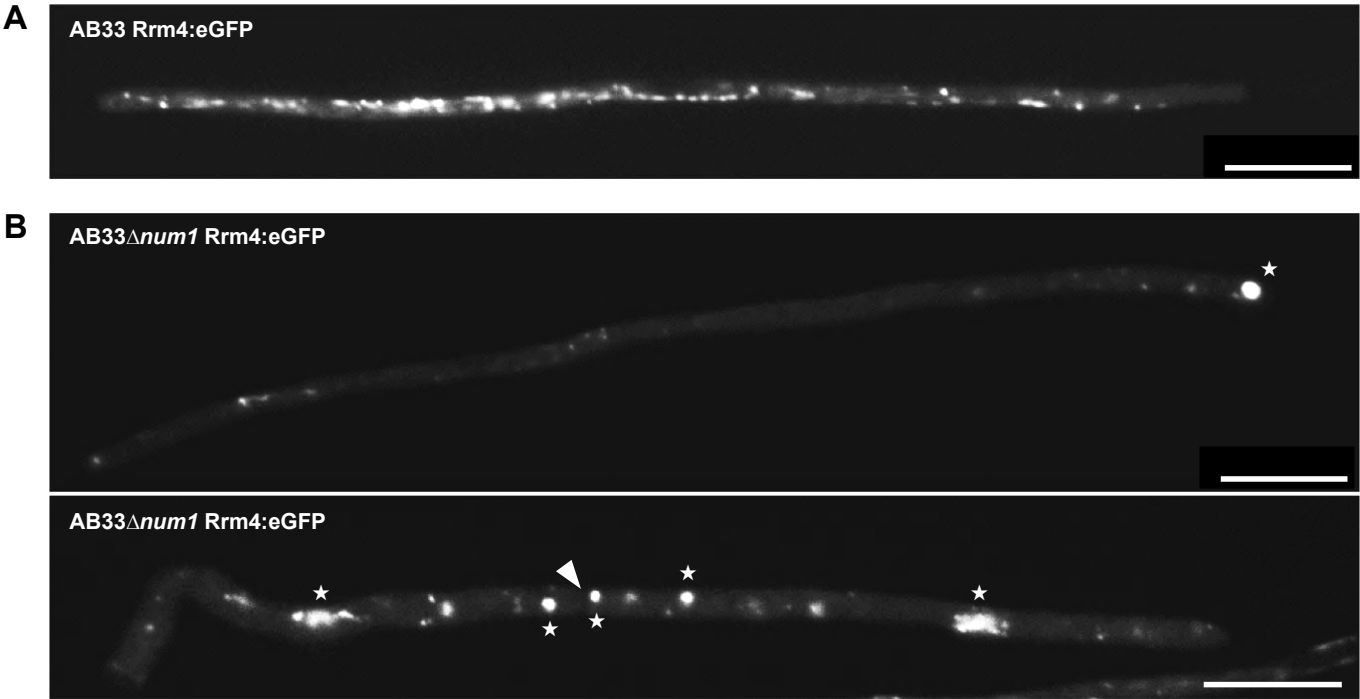

Supplement: Figure S22 — Num1 influences the dynamic of the RNA-binding protein Rrm4. To analyze the impact of num1 on Rrm4 movement, an Rrm4:eGFP fusion protein was expressed in AB33 and AB33Δnum1. bE1/bW2 were induced for 16 hours in nitrate minimal medium, and Rrm4:eGFP localization was analyzed by fluorescence microscopy. (A) In AB33, Rrm4:eGFP-fusion proteins localize on bidirectional moving particles that are evenly distributed within the hyphae, as previously described [46]; see Supporting Video S3. (B) In AB33Δnum1 the Rrm4-particles accumulate at the hyphal tip (asterisk, upper panel) or within the hyphae (lower panel); see also Supporting Video S4 Arrowhead indicates septum. Scale bars: 10 µm. (PDF) [file pgen.1004046.s022.pdf]
