# Supplementary material for: The SPF27 Homologue Num1 Connects Splicing and Kinesin 1-Dependent Cytoplasmic Trafficking in Ustilago maydis
Source: PLoS Genet. 2014 Jan 2;10(1):e1004046. doi: 10.1371/journal.pgen.1004046 (PMC3879195; doi:10.1371/journal.pgen.1004046)
Supplement: Figure S12 — Individual genes show different alterations in intron retention in AB31Δnum1. Splicing efficiency of the indicated genes based on the RNA-Seq analysis. Depicted are examples for genes (A) where splicing is not affected, (B) the intron is not spliced at all, or (C) different introns show dissimilar retention rates. Plotted are the FPKM values (fragments per kilobase of sequence per million fragments mapped) across the genomic region indicated (coordinates in nucleotides) of three independent RNA-Seq experiments for AB31 wild-type (blue lines) and AB31Δnum1 (red lines), respectively. Exons (E) and introns (I) are indicated. (PDF) [file pgen.1004046.s012.pdf]

Figure_S12

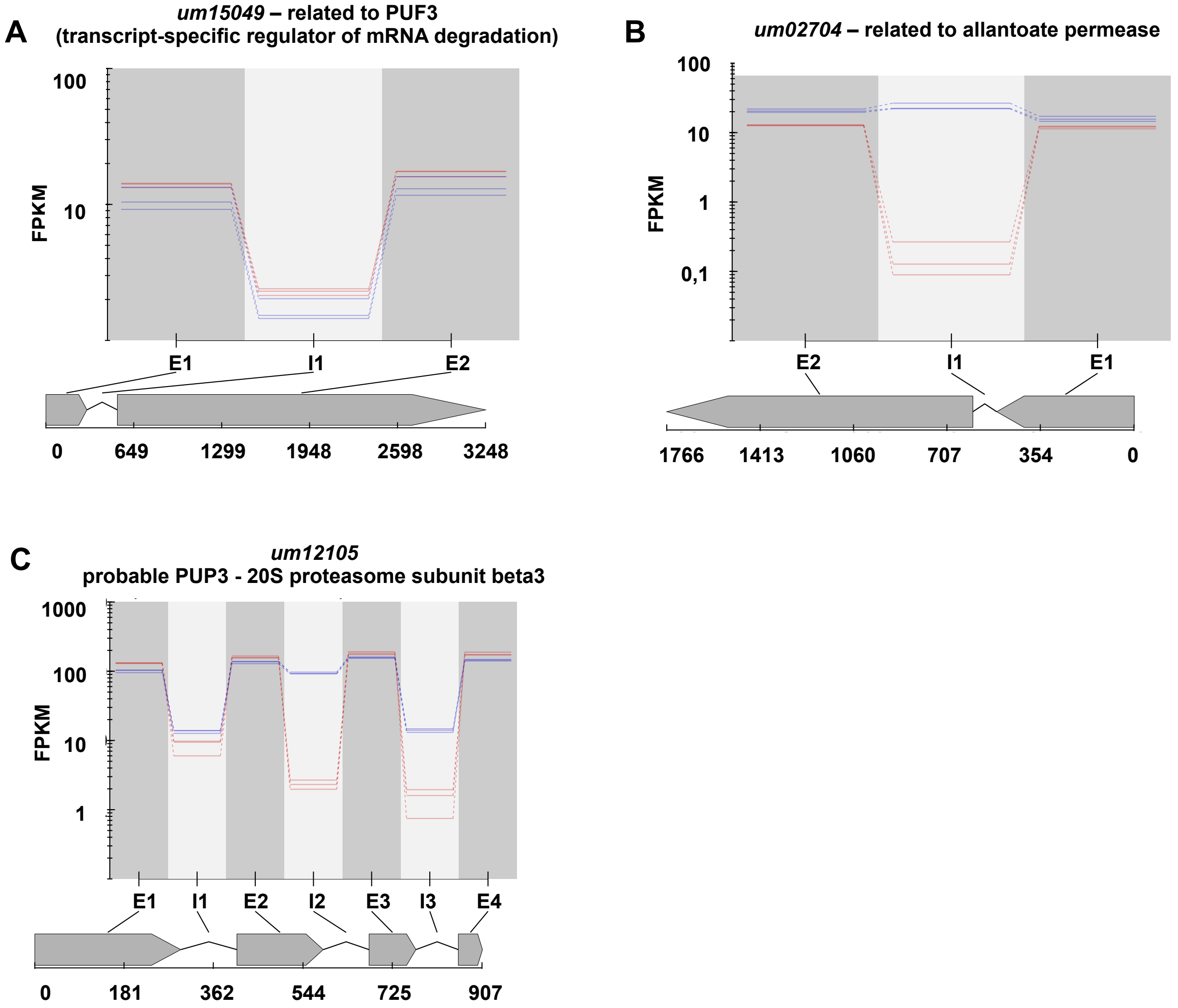

A
um15049 – related to PUF3
(transcript-specific regulator of mRNA degradation)
100
10
FPKM
E1
I1
E2
0
649
1299
1948
2598
3248
B
um02704 – related to allantoate permease
100
10
1
0,1
FPKM
E2
I1
E1
1766
1413
1060
707
354
0
C
um12105
probable PUP3 - 20S proteasome subunit beta3
1000
100
10
1
FPKM
E1
I1
E2
I2
E3
I3
E4
0
181
362
544
725
907
