# Supplementary material for: The SPF27 Homologue Num1 Connects Splicing and Kinesin 1-Dependent Cytoplasmic Trafficking in Ustilago maydis
Source: PLoS Genet. 2014 Jan 2;10(1):e1004046. doi: 10.1371/journal.pgen.1004046 (PMC3879195; doi:10.1371/journal.pgen.1004046)
Supplement: Text S1 — Prp19:3×HA and Cef1:3×HA fusion proteins are functional in U. maydis. (DOCX) [file pgen.1004046.s027.docx]

**Supporting Text S1: Prp19:3xHA and Cef1:3xHA fusion proteins are functional in *U. maydis.***

For use in co-immunoprecipitation and cellular, *prp19* (*um10027*) and *cef1* (*um04411*) were exchanged with HA-tagged versions via homologous recombination in strains UNK200 (AB31 *num1:3xeGFP:hyg^R^, prp19:3xHA:cbx^R^)* and UMO8 (AB31 *num1:3xeGFP:hyg^R^, cef1:3xHA:cbx^R^)*, and with RFP-tagged versions in strains UNK208 (AB31 *num1:3egfp:hyg^R^, prp19:rfp:nat^R^*) and UMO10 (AB31 *num1:3egfp:hyg^R^, cef1:rfp:nat^R^*).

In both *S. cerevisiae* and *S. pombe*, Prp19 and Cdc5/Cef1 were shown to be essential proteins. Similarly, we failed to generate *prp19* or *cef1* deletion mutants in the haploid strain SG200, suggesting that both genes are essential in *U. maydis*. For further analysis, we replaced one copy of *prp19* or *cef1*, respectively, with a hygromycin resistance cassette in the diploid strain FBD11 (*a1 a2 b1 b2*) [[1](#_ENREF_1)]. Diploid teliospores were harvested from tumors originating from plants infected with FBD11*∆prp19* and FBD11*∆cef1.* Analysis of the haploid meiotic progeny of germinated teliospores revealed no hygromycin-resistant cells, demonstrating that those cells in which the *prp19* and *cef1* genes are replaced with the hygromycin-marker gene are not viable. As *U. maydis* strains UNK200 and UMO8, which harbor HA tagged versions of *prp19* and *cef1*, as well as UNK208 and UMO10 are all viable and show no other obvious phenotypes, we conclude that Prp19:3xHA and Cef1:3xHA fusion proteins are both functional. The RFP fusion proteins in strains UNK208 and UMO10 can be visualized in the nucleus (Figure S7)

**Reference:**

1. Banuett F, Herskowitz I (1989) Different *a* alleles of *Ustilago maydis* are necessary for maintenance of filamentous growth but not for meiosis. Proc Natl Acad Sci USA 86: 5878-5882.
