# Supplementary material for: The SPF27 Homologue Num1 Connects Splicing and Kinesin 1-Dependent Cytoplasmic Trafficking in Ustilago maydis
Source: PLoS Genet. 2014 Jan 2;10(1):e1004046. doi: 10.1371/journal.pgen.1004046 (PMC3879195; doi:10.1371/journal.pgen.1004046)
Supplement: Table S6 — Num1 interacting proteins identified by Yeast Two-Hybrid screening. (DOCX) [file pgen.1004046.s033.docx]

**Table S6:**

Num1 interacting proteins identified by Yeast-Two-Hybrid screening.

|  | **Accession**^a^ | **No. of isolation**^b^ | **Description**^c^ |
| --- | --- | --- | --- |
| 1 | *um06098* | 4 | related to extracellular elastinolytic metalloproteinase precursor |
| 2 | *um11400* | 20 | probable thiamin biosynthetic enzyme |
| 3 | *um03539* | 4 | conserved hypothetical protein (BAR domain) |
| 4 | *um03416* | 2 | Crg1, carbon source-regulated protein |
| 5 | *um00029* | 2 | probable myo-inositol oxygenase |
| 6 | *um10905* | 2 | conserved hypothetical protein |
| 7 | *um02300* | 1 | probable alpha-amylase |
| 8 | *um10787* | 3 | probable Fum1 – fumarate hydratase |
| 9 | *um01172* | 3 | probable Sdh1 – succinate dehydrogenase (ubiquinone) flavoprotein percursor |
| 10 | *um00658* | 2 | probable 40S ribosomal protein S13.e |
| 11 | *um04138* | 1 | probable Tal1 - transaldolase |
| 12 | *um03584* | 1 | related to Rpa49 – 49 kD subunit of DNA-directed RNA polymerase I |
| 13 | *um05831* | 1 | probable heat-shock protein hsp60 |
| 14 | *um00933* | 2 | conserved hypothetical protein |
| 15 | *um12306* | 1 | related to Med7 – member of RNA polymerase II transcriptional regulation mediator complex |
| 16 | *um11630* | 1 | conserved hypothetical protein (thioesterase superfamily) |
| 17 | *um10158* | 1 | probable adapter-related protein complex 1 beta 1 subunit (adaptin-like) |
| 18 | *um11510* | 2 | conserved hypothetical protein (BRO domain) |
| 19 | *um05042.2* | 1 | conserved hypothetical protein |
| 20 | *um04218* | 1 | Kin1 – Kinesin 1 motor protein |
| 21 | *um11067* | 1 | probable catalase 2 |
| 22 | *um10174* | 1 | conserved hypothetical protein (Hamartin domain) |
| 23 | *um04887* | 1 | conserved hypothetical Ustilago-specific protein |
| 24 | *um10958* | 1 | related to kinetochore-associated 2 (HEC) |
| 25 | *um05908* | 1 | conserved hypothetical protein |
| 26 | *um06285* | 1 | related to carbamoyl-phosphate synthase small chain, arginin-specific, small chain, mitochondrial precursor |
| 27 | *um00523* | 1 | related to Cdc36 – transcription factor |
| 28 | *um11022* | 1 | related to Sry1 – 3-hydroxyaspartate dehydratase |
| 29 | *um10394* | 1 | related to dihydrofolate reductase |
| 30 | *um02361* | 1 | related to ATPase inhibitor, mitochondrial precursor |
| 31 | *um10672.2* | 1 | hypothetical protein |
| 32 | *um05378* | 1 | Dnm1 – mitochondrial fission factor DNM1 |
| 33 | *um04411* | 1 | related to Cef1 – required during G2/M transition |

^a^ = Accession according to MUMDB (http://mips.helmholtz-muenchen.de/genre/proj/ustilago/)

^b^ = number of times of isolation

^c^ = functional description/gene name according to MUMDB (http://mips.helmholtz-muenchen.de/genre/proj/ustilago/)
