# Supplementary material for: The SPF27 Homologue Num1 Connects Splicing and Kinesin 1-Dependent Cytoplasmic Trafficking in Ustilago maydis
Source: PLoS Genet. 2014 Jan 2;10(1):e1004046. doi: 10.1371/journal.pgen.1004046 (PMC3879195; doi:10.1371/journal.pgen.1004046)
Supplement: Table S7 — U. maydis strains used in this study. (DOCX) [file pgen.1004046.s034.docx]

**Table S7:** *U. maydis* strains used in this study.

| **Strain** | **Genotype** | **Reference** |
| --- | --- | --- |
| SG200 | *a1:mfa2, bE1, bW2, ble^R^* | [[1](#_ENREF_1)] |
| AB31 | *a2, P_crg1_:bW2, bE1, ble^R^* | [[2](#_ENREF_2)] |
| FB1 | *a1, b1* | [[3](#_ENREF_3)] |
| FB2 | *a2, b2* | [[3](#_ENREF_3)] |
| FBD11 | *a1/a2, b1/b2, ade^−/+^, pan^−/+^* | [[3](#_ENREF_3)] |
| SG200*∆num1* | *a1:mfa2, bE1, bW2, ble^R^,* *∆num1::hyg^R^* | this work |
| AB31*∆num1* | *a2, P_crg1_:bW2, bE1, ble^R^, ∆num1::hyg^R^* | this work |
| FB1*∆num1* | *a1, b1, ∆num1::hyg^R^* | this work |
| FB2*∆num1* | *a2, b2, ∆num1::hyg^R^* | this work |
| FBD11*∆prp19* | *a1/a2, b1/b2, ade^−/+^, pan^−/+^, ∆prp19::hyg^R^* | this work |
| FBD11*∆cef1* | *a1/a2, b1/b2, ade^−/+^, pan^−/+^, ∆cef1::hyg^R^* | this work |
| SG200*∆kin1** | *a1:mfa2, bE1, bW2, ble^R^, ∆kin1::hyg^R^* | this work |
| SG200*∆rbf1* | *a1:mfa2, bE1, bW2, ble^R^, ∆rbf1::hyg^R^* | [[4](#_ENREF_4)] |
| SG200 *rbf1::rbf1cDNA* | *a1:mfa2, bE1, bW2, rbf1::rbf1cDNA:3’-UTR:cbxR* | this work |
| SG200*∆rbf1 rbf1::rbf1cDNA* | *a1:mfa2, bE1, bW2, ∆rbf1::hyg^R^, /P_rbf1_:rbf1cDNA, cbx^R^* | this work |
| AB31 *rbf1::rbf1cDNA* (UTO62) | *a2, P_crg1_: bW2, bE1, rbf1::rbf1cDNA:3’-UTR:cbxR* | this work |
| AB31*∆num1 rbf1::rbf1cDNA* | *a2, P_crg1_: bW2, bE1, ∆num1::hyg^R^, rbf1::rbf1cDNA:3’-UTR:cbxR* | this work |
| AB31*∆kin1** | *a2, P_crg1_:bW2, bE1, ble^R^, ∆kin1::hyg^R^* | this work |
| SG200*∆num1∆kin1** | *a1:mfa2, bE1, bW2, ble^R^, ∆kin1::hyg^R^, ∆num1::nat^R^* | this work |
| AB31*∆num1∆kin1** | *a2, P_crg1_:bW2, bE1, ble^R^, ∆kin1::nat^R^, ∆num1::hyg^R^* | this work |
| FB1/pKS2 | *a1, b1, /P_crg1_:mtRFP* | [[5](#_ENREF_5)] |
| FB1*∆num1*/pKS2 | *a1, b1, ∆num1::hyg^R^, /P_crg1_:mtRFP* | this work |
| FB2/pKS1 | *a2, b2, /P_crg1_:mtGFP* | [[5](#_ENREF_5)] |
| FB2*∆num1*/pKS2 | *a2, b2, ∆num1::hyg^R^, /P_crg1_:mtGFP* | this work |
| AB31 Prp19-HA Num1-GFP  (UNK200) | *a2, P_crg1_:bW2, bE1, ble^R^, prp19:3ha, cbx^R^, num1:3egfp, hyg^R^* | this work |
| AB31 Cef1-HA Num1-GFP  (UMO8) | *a2, P_crg1_:bW2, bE1, ble^R^, cef1:3ha, cbx^R^, num1:3egfp, hyg^R^* | this work |
| AB31 Prp19-RFP Num1-GFP (UNK208) | *a2, P_crg1_:bW2, bE1, ble^R^, prp19:rfp, nat^R^, num1:3egfp, hyg^R^* | this work |
| AB31 Cef1-RFP Num1-GFP  (UMO10) | *a2, P_crg1_:bW2, bE1, ble^R^, cef1:rfp, nat^R^, num1:3egfp, hyg^R^* | this work |
| AB31 Kin1*-HA Num1-GFP | *a2, P_crg1_:bW2, bE1, ble^R^, kin1:3ha, cbx^R^, num1:3egfp, hyg^R^* | this work |
| SG200 Num1-GFP | *a1:mfa2, bE1, bW2, ble^R^, num1:3egfp*, *hyg^R^* | this work |
| AB31 Num1-GFP | *a2, P_crg1_:bW2, bE1, ble^R^, num1:3egfp*, *hyg^R^* | this work |
| AB31 Rbf1-HA | *a2, P_crg1_:bW2, bE1, ble^R^, rbf1:3ha*, *hyg^R^* | [[4](#_ENREF_4)] |
| AB31*∆num1* Rbf1-HA | *a2, P_crg1_:bW2, bE1, ble^R^, ∆num1::nat^R^, rbf1:3ha*, *hyg^R^* | this work |
| AB31 Num1-GFP Kin1*-RFP | *a2, P_crg1_:bW2, bE1, ble^R^, num1:3egfp*, *hyg^R^, kin1:rfp, nat^R^* | this work |
| AB33 GFP-Dyn2 | *a2, P_nar_:bW2, P_nar_:bE1* *3egfp:dyn2, ble^R^, nat^R^* | [[6](#_ENREF_6)] |
| AB33*∆num1* GFP-Dyn2 | *a2, P_nar_:bW2, P_nar_:bE1,* *3egfp:dyn2, ble^R^, nat^R^, ∆num1::cbx^R^* | this work |
| AB33 Yup1-GFP | *a2, P_nar_:bW2, P_nar_:bE1, ble^R^, ip^R^, P_otef_:yup1:egfp, ip^S^* | [[6](#_ENREF_6)] |
| AB33*∆num1* Yup1-GFP | *a2, P_nar_:bW2, P_nar_:bE1, ble^R^, ip^R^, P_otef_:yup1:egfp, ip^S^, ∆num1::hyg^R^* | this work |
| AB33 GFP-Kin1* | *a2, P_nar_:bW2, P_nar_:bE1, ble^R^, /P_kin1_:3egfp:kin1, cbx^R^* | [[7](#_ENREF_7)] |
| AB33*∆num1* GFP-Kin1* | *a2, P_nar_:bW2, P_nar_:bE1, ble^R^, ∆num1::hyg^R^, /P_kin1_:3egfp:kin1, cbx^R^* | this work |
| AB31 Tub1-mCherry | *a2, P_crg1_:bW2, bE1, ble^R^, ip^R^, P_otef_:tub1:mCherry, ip^S^* | this work |
| AB31*∆num1* Tub1-mCherry | *a2, P_crg1_:bW2, bE1, ble^R^, ip^R^, P_otef_:tub1:mCherry, ip^S^, ∆num1::hyg^R^* | this work |
| AB31 LifeAct-YFP | *a2, P_crg1_:bW2, bE1, ble^R^, ip^R^, P_otef_:lifeact:yfp, ip^S^* | this work |
| AB31*∆num1* LifeAct-YFP | *a2, P_crg1_:bW2, bE1, ble^R^, ip^R^, P_otef_:lifeact:yfp, ip^S^, ∆num1::hyg^R^* | this work |
| AB31*∆num1* Num1:eGFP | *a2, P_crg1_:bW2, bE1, ble^R^, ip^R^, P_crg1_:num1:egfp, ip^S^, ∆num1::hyg^R^* | this work |
| AB31*∆num1* Num1_mutNLS_ | *a2, P_crg1_:bW2, bE1, ble^R^, ip^R^, P_crg1_:num1_mutNLS_:egfp, ip^S^, ∆num1::hyg^R^* | this work |
| AB33 Peb1-RFP GFP-Tub1 | *a2, P_nar_:bW2, P_nar_:bE1, ble^R^, peb1:rfp, nat^R^, /gfp:tub1, cbx^R^* | [[7](#_ENREF_7)] |
| AB33*∆num1* Peb1-RFP GFP-Tub1 | *a2, P_nar_:bW2, P_nar_:bE1, ble^R^, peb1:rfp, nat^R^, /gfp:tub1, cbx^R^, ∆num1::hyg^R^* | this work |
| AB33 GFP-Mcs1 | *a2, P_nar_:bW2, P_nar_:bE1, ble^R^, 3egfp:mcs1, hyg^R^,* | [[8](#_ENREF_8)] |
| AB33*∆num1* GFP-Mcs1 | *a2, P_nar_:bW2, P_nar_:bE1, ble^R^, 3egfp:mcs1, hyg^R^, ∆num1::cbx^R^* | this work |

Abbreviations in strain genotypes: *a*, *b*: mating type loci: *mfa2*: mating pheromone; *∆*: deletion; P: promoter; ::, homologous replacement; :, fusion; *hyg^R^*: hygromycin resistance; *ble^R^*: phleomycin resistance; *nat^R^*, nourseothricin resistance: *cbx^R^*, carboxin resistance: /, ectopically integrated; *P_otef_*: constitutive promoter [[9](#_ENREF_9)]; *P_nar_*: conditional nitrate reductase promoter [[10](#_ENREF_10)]; *P_crg1_*: conditional arabinose-induced promoter [[11](#_ENREF_11)]; *E1*, *W2*: genes of the *b* mating type locus; *egfp*: enhanced green fluorescent protein; *yfp*: yellow-shifted fluorescent protein; *yup1*: endosomal t-SNARE; *dyn2*: C-terminal half of the dynein heavy chain; *tub1*: α-tubulin.

* *kin1*, conventional kinesin (kinesin-1), previously named *kin2* [[7](#_ENREF_7),[12](#_ENREF_12)]

**References:**

1. Kämper J, Kahmann R, Bölker M, Ma LJ, Brefort T, et al. (2006) Insights from the genome of the biotrophic fungal plant pathogen *Ustilago maydis*. Nature 444: 97-101.

2. Brachmann A, Weinzierl G, Kämper J, Kahmann R (2001) Identification of genes in the bW/bE regulatory cascade in *Ustilago maydis*. Mol Microbiol 42: 1047-1063.

3. Banuett F, Herskowitz I (1989) Different *a* alleles of *Ustilago maydis* are necessary for maintenance of filamentous growth but not for meiosis. Proc Natl Acad Sci USA 86: 5878-5882.

4. Heimel K, Scherer M, Vranes M, Wahl R, Pothiratana C, et al. (2010) The transcription factor Rbf1 is the master regulator for *b*-mating type controlled pathogenic development in *Ustilago maydis*. PLoS Pathog 6: e1001035.

5. Mahlert M, Vogler C, Stelter K, Hause G, Basse CW (2009) The *a2* mating-type-locus gene *lga2* of *Ustilago maydis* interferes with mitochondrial dynamics and fusion, partially in dependence on a Dnm1-like fission component. J Cell Sci 122: 2402-2412.

6. Lenz JH, Schuchardt I, Straube A, Steinberg G (2006) A dynein loading zone for retrograde endosome motility at microtubule plus-ends. EMBO J 25: 2275-2286.

7. Schuchardt I, Assmann D, Thines E, Schuberth C, Steinberg G (2005) Myosin-V, Kinesin-1, and Kinesin-3 cooperate in hyphal growth of the fungus *Ustilago maydis*. Mol Biol Cell 16: 5191-5201.

8. Schuster M, Treitschke S, Kilaru S, Molloy J, Harmer NJ, et al. (2012) Myosin-5, kinesin-1 and myosin-17 cooperate in secretion of fungal chitin synthase. EMBO J 31: 214-227.

9. Spellig T, Bottin A, Kahmann R (1996) Green fluorescent protein (GFP) as a new vital marker in the phytopathogenic fungus *Ustilago maydis*. Mol Gen Genet 252: 503-509.

10. Banks GR, Shelton PA, Kanuga N, Holden DW, Spanos A (1993) The *Ustilago maydis nar1* gene encoding nitrate reductase activity: sequence and transcriptional regulation. Gene 131: 69-78.

11. Bottin A, Kämper J, Kahmann R (1996) Isolation of a carbon source-regulated gene from *Ustilago maydis*. Mol Gen Genet 253: 342-352.

12. Lehmler C, Steinberg G, Snetselaar K, Schliwa M, Kahmann R, et al. (1997) Identification of a motor protein required for filamentous growth in *Ustilago maydis*. EMBO J 16: 3464-3473.
