# Supplementary material for: The SPF27 Homologue Num1 Connects Splicing and Kinesin 1-Dependent Cytoplasmic Trafficking in Ustilago maydis
Source: PLoS Genet. 2014 Jan 2;10(1):e1004046. doi: 10.1371/journal.pgen.1004046 (PMC3879195; doi:10.1371/journal.pgen.1004046)
Supplement: Table S8 — A. nidulans strains used in this study. (DOCX) [file pgen.1004046.s035.docx]

**Table S8:** *A. nidulans* strains used in this study.

| **Strain** | **Genotype** | **Reference** |
| --- | --- | --- |
| TNO2A3 | *pyrG89-; argB2-; ∆nkuA::argB; pyroA4-* | [[1](#_ENREF_1)] |
| TNO2A3*∆AN4244* | *pyrG89-; argB2-; ∆nkuA::argB; pyroA4-; ∆AN4244::pyroA4* | this work |
| LZ12 | *gfp-nudA; ∆nkuA::argB; pyroA4-; pyrG89-* | [[2](#_ENREF_2)] |
| LZ12*∆AN4244* | *gfp-nudA; ∆nkuA::argB; pyroA4-; pyrG89-; ∆AN4244::pyroA4* | this work |

**References:**

1. Nayak T, Szewczyk E, Oakley CE, Osmani A, Ukil L, et al. (2006) A versatile and efficient gene-targeting system for *Aspergillus nidulans*. Genetics 172: 1557-1566.

2. Zhuang L, Zhang J, Xiang X (2007) Point mutations in the stem region and the fourth AAA domain of cytoplasmic dynein heavy chain partially suppress the phenotype of NUDF/LIS1 loss in *Aspergillus nidulans*. Genetics 175: 1185-1196.
