# Supplementary material for: The SPF27 Homologue Num1 Connects Splicing and Kinesin 1-Dependent Cytoplasmic Trafficking in Ustilago maydis
Source: PLoS Genet. 2014 Jan 2;10(1):e1004046. doi: 10.1371/journal.pgen.1004046 (PMC3879195; doi:10.1371/journal.pgen.1004046)
Supplement: Table S9 — Oligonucleotides used in this study. (DOCX) [file pgen.1004046.s036.docx]

**Table S9:** Oligonucleotides used in this study

| **Primer/Purpose** | **Sequence (5’ 🡪 3’ direction)** | **Reference** |
| --- | --- | --- |
| **Yeast two-hybrid** |  |  |
| num1_Y2H_for | GTGGGCCATTACGGCCATGGCGAGAGCACGAGGTGGAT | this work |
| num1_Y2H_rev | ATAGGCCGAGGCGGCCCTACTCCAACAGGCTCAACTCTGTC | this work |
| pGAD-DS_for | TACGACTCACTATAGGGCGAGCGCC | [[1](#_ENREF_1)] |
| pGAD-DS_rev | TGGTGCACGATGCACAGTTGAAGTG | [[1](#_ENREF_1)] |
| 10027_Y2H_full_se | GTGGGCCATTACGGCCATGTTCTGCGCCATCTCTGGTGAG | this work |
| 10027_Y2H_full_as | ATAGGCCGAGGCGGCCTCAGGCGGCGAGACCAAACG | this work |
| 10027_Y2H_Cterm_se | GTGGGCCATTACGGCCCCGTGGCAGACTTTGTAGAGGTGC | this work |
| 10027_Y2H_N_as | ATAGGCCGAGGCGGCCCCGGTGCTTTACGTTTGGACTTGG | this work |
| 04411_Y2H_se | GTGGGCCATTACGGCCATGGTTCGCGTCATCATCAAAGGC | this work |
| 04411_Y2H_as | ATAGGCCGAGGCGGCCCTAGGCAGACAGATTAGCCTCGTTG | this work |
|  |  |  |
| ***num1* deletion** |  |  |
| 01682_LB_se | GACGCCGTACATGTCCCAGATTTC | this work |
| 01682_LB_as | GTTGGCCATCTAGGCCTGGATGGAGTAGCAGCGGCTTTC | this work |
| 01682_RB_se | GTTGGCCTGAGTGGCCGCAGTGATGCGGTTCATGTGTCG | this work |
| 01682_RB_as | TCCCACAGGGGACTCGAAGAATC | this work |
| 01682_nested_se | GTACCAGTCTGCGGATCGGAAGAC | this work |
| 01682_nested_as | TTGCGGCAGATTCTTCCAACTCC | this work |
| 01682_ORF_se | AACACCTTGGCTGCCACATCGAC | this work |
| 01682_ORF_as | CTGTTGGAAATTGGACAGCCTCC | this work |
|  |  |  |
| ***prp19* deletion** |  |  |
| 10027_LB_se | CCCGTGACATGAGAAGCTTAACGTC | this work |
| 10027_LB_as | GTTGGCCATCTAGGCCGTTGACGCGATTGGCCCTTCT | this work |
| 10027_RB_se | GTTGGCCTGAGTGGCCTCGCCGTCTCATTTCTTTTGTTTACC | this work |
| 10027_RB_as | GGAATATTTATCGTCAAGGCGCTCG | this work |
| 10027_nested_se | CGTGTTTCTGTCCTGGTCGGC | this work |
| 10027_nested_as | CCGTCCGACTGTGGTTGGTGC | this work |
| 10027_ORF_se | GTGGGCCATTACGGCCATGTTCTGCGCCATCTCTGGTGAG | this work |
| 10027_ORF_as | CGAACGGTACGGTCCAGACTCG | this work |
|  |  |  |
| ***cef1* deletion** |  |  |
| 04411_LB_se | CTGGCAGCTGCCTCCCTGATTCT | this work |
| 04411_LB_as | GTTGGCCATCTAGGCCGTTGACAATGAGAAGCCTGACAGC | this work |
| 04411_RB_se | GTTGGCCTGAGTGGCCTAGTTGTGGCCAAGCACAGATCATG | this work |
| 04411_RB_as | CTGTGAGCCTTGAAAGTCTGCCA | this work |
| 04411_nested_se | TCTGTGGATAACAAGTCATGCTGCTC | this work |
| 04411_nested_as | TAAACGGACAACACAGACCTGCG | this work |
| 04411_ORF_se | ATGGTTCGCGTCATCATCAAAGG | this work |
| 04411_ORF_as | ATACGCGCCCACTGATTCTTGCC | this work |
|  |  |  |
| ***kin1* deletion** |  |  |
| kin1_LB_se | GTGTGCTTTGTGGAGGAAGTCCGAC | this work |
| kin1_LB_as | GTTGGCCATCTAGGCCGACGACTTGGCGGTGTAAGTGTGC | this work |
| kin1_RB_se | GTTGGCCTGAGTGGCCCGTGGAAGATCTATACGTTTCACCTT | this work |
| kin1_RB_as | GCAATCTCAAGCGTCGCTATGA | this work |
| kin1_nested_se | CCCACTCGACTCTGTGCCTTGTGAC | this work |
| kin1_nested_as | CCAACACGGCTGCTTCCATTTCC | this work |
| kin1_ORF_se | ATGTCCAACAACATCAAGGTCGTC | this work |
| kin1_ORF_as | CGAACTCAAAGACGTCGCGTTG | this work |
|  |  |  |
| **C-terminal *num1* fusion** |  |  |
| 01682GFP_LB_se | CCAATTCTTAACGGCCGAAAAGAC | this work |
| 01682GFP_LB_as | GTGGGCCGCGTTGGCCCGCTCCAACAGGCTCAACTC | this work |
| 01682GFP_RB_Sfi | CACGGCCTGAGTGGCCTAGGCAGTGATGCGGTTCAT | this work |
| 01682GFP_RB_as | GACGGCATCGTTGCGTTGGAAG | this work |
|  |  |  |
| **C-terminal *prp19* fusion** |  |  |
| 10027fusion_LB_se | CCGTGGCAGACTTTGTAGAGGTGC | this work |
| 10027fusion_LB_as | GGCCGCGTTGGCCCGGGCGGCGAGACCAAACGC | this work |
| 10027fusion_RB_se | GGCCTGAGTGGCCTCGCCGTCTCATTTCTTTTGTTTACC | this work |
| 10027fusion_RB_as | GGAATATTTATCGTCAAGGCGCTCG | this work |
|  |  |  |
| **C-terminal *cef1* fusion** |  |  |
| 04411fusion_LB_se | CTCAAGCGGGCACGTAGCCTG | this work |
| 04411fusion_LB_as | GGCCGCGTTGGCCCGGGCAGACAGATTAGCCTCGTTGAGAG | this work |
| 04411fusion_RB_se | GGCCTGAGTGGCCTCGCCGTCTCATTTCTTTTGTTTACC | this work |
| 04411fusion_RB_as | CTGTGAGCCTTGAAAGTCTGCCA | this work |
|  |  |  |
| **C-terminal *kin1* fusion** |  |  |
| kin1fusion_LB_se | GGAGAACGAGCTACTGCATCAAAGG | this work |
| kin1fusion_LB_as | GGCCGCGTTGGCCCGCTTGGACGAAAAGAACCAGCTGG | this work |
| kin1fusion_RB_se | GGCCTGAGTGGCCCACCTTACCTTACATACCCGATCTCTG | this work |
| kin1fusion_RB_as | GCAATCTCAAGCGTCGCTATGA | this work |
|  |  |  |
| ***num1* truncation** |  |  |
| 01682_LB_se | GACGCCGTACATGTCCCAGATTTC | this work |
| 01682trunc_LB_as | GGCCGCGTTGGCCCGTGTGGAAGAGATGGGAGACATGGAC | this work |
| 01682_Nde | CATATGGCGAGAGCACGAGGTGGATG | this work |
| 01682trunc_Not | AGCGGCCGCCTATGTGGAAGAGATGGGAGACATGGAC | this work |
|  |  |  |
| ***num1*∆NLS** |  |  |
| num1_seNde | CATATGGCGAGAGCACGAGGTGGATG | this work |
| num1_dNLS_Sfi | GGCCGCGTTGGCCCGCTCCAACAGGCTCAACTCTGTCTCGAGCGCAGCGGCGGCACTTTTC | this work |
|  |  |  |
| **qRT PCR analyses** |  |  |
| Ubi1_RTas | CATCCTCGAGCTGCTTACCG | this work |
| Ubi1_RTintron | CAGTACGGCAAGCTAACGCATTC | this work |
| Ubi1_RTborders | CTCTGACGGGTAAGACCATTACC | this work |
| Rho3_RTas | CTTGTCAAACTCCTCCTGACCAG | this work |
| Rho3_RTintron | GTGGCTGGCTAGAACGACTG | this work |
| Rho3_RTborders | CACAGACATACGAGCCGACG | this work |
| Cts1_RTas | GGAAGCTTCGGGTGCCGTA | this work |
| Cts1_RTintron | GCACAGATGACTCAATGCAGCA | this work |
| Cts1_RTborders | ATGTTTGGACGTCTTAAGCACAGG | this work |
| PCNA_RTas | GACACCGATCGACATAGGTC | this work |
| PCNA_RTintron | GCAACGTCGCATTTGATCTGG | this work |
| PCNA_RTborders | GACGGCATTCGACTGCAAG | this work |
| Rpb3_RTas | CGAGAAGTCACCTCGAGATTAC | this work |
| Rpb3_RTintron | CGTGTCTCTATCTTGCTCTCTTC | this work |
| Rpb3_RTborders | GGATCACAGGGATTGCGCATG | this work |
| Rbf1_RTborders1 | ATGGACATCTTGGAACTAGTGCAC | this work |
| Rbf1_RTintron1 | CAAGCGATTGACCTCTCCTCG | this work |
| Rbf1_RTas1 | CTAGAAAACGCTTTGGGACAACC | this work |
| Rbf1_RTborders3 | CTTGGCACGACACGCTCG | this work |
| Rbf1_RTintron3 | ACGATGCTGATTCATCAAGCCG | this work |
| Rbf1_RTas3 | GAATGAACGTCTTTCCGCAGTC | this work |
| Rbf1_RTborders4 | CAGCAAAGCATTCTCTGACTCGA | this work |
| Rbf1_RTintron4 | GCGCTCCTCAGGCTCATTG | this work |
| Rbf1_RTas4 | CAAGGCACTTGTACGGACGTC | this work |
| RT_eIF2b_f | ATCCCGAACAGCCCAAAC | [[1](#_ENREF_1)] |
| RT_eIF2b_r | ATCGTCAACCGCAACCAC | [[1](#_ENREF_1)] |
| RT_actin_f | CATGTACGCCGGTATCTCG | [[1](#_ENREF_1)] |
| RT_actin_r | CTCGGGAGGAGCAACAATC | [[1](#_ENREF_1)] |
| RT_bE-F | GCACAACACCTTCCATTGAC | [[2](#_ENREF_2)] |
| RT_bE-R | ACTGCTCCCGAATGTACTG | [[2](#_ENREF_2)] |
| RT_bW-F | GATCTCACCCAGCCAATCAC | [[2](#_ENREF_2)] |
| RT_bW-R | GAGTTGATCGAGGCCGAATG | [[2](#_ENREF_2)] |
| RT_rbf1-F | AGTACGAGCTACGACGGATTC | [[2](#_ENREF_2)] |
| RT_rbf1-R | GGGTAGGTGTTGGACACATTC | [[2](#_ENREF_2)] |
| 01682RT_for | CAACTGGGCCATATGGATGTTCG | this work |
| 01682RT_rev | TCGAGCTGTTCGGATAGCAGACG | this work |
| RT_riz_f | AACATGCAAGGCTCAGGTAG | this work |
| RT_riz-R | CTGTCCATCATTTGGCTGATCC | this work |
| RT_hdp2-F | GGCGCTTTGCATTGGAAC | this work |
| RT_hdp2_R | AGCTTGAAGCCGATCGAC | this work |
| RT_hdp1-F6 | CCGAAAGCGTCTGGGATGAG | this work |
| RT_hdp1-R6 | GTCGTGCGTACATCGTACGG | this work |
|  |  |  |
| **RT PCR analysis** |  |  |
| Rbf1_PCR_for | ACAGTTTGCCAAAATGGACATCTTG | this work |
| Rbf1_PCR_rev | GGTGAACGTACCTTCGGCATC | this work |
|  |  |  |
| ***A. nidulans* *AN4244* deletion** |  |  |
| Num1_A_nid_LBs | GCCATTGCAGACTAGCTTCGAGC | this work |
| Num1_A_nid_LBas | GTTGGCCATCTAGGCCGTGAATAGCTGTCTTGGCTGCT | this work |
| Num1_A_nid_RBs | GTTGGCCTGAGTGGCCAAGTGTCATGCCCTGATTAC | this work |
| Num1_A_nid_RBas | GAGGAGACTACAGCCGGCAGTTTT | this work |
| Num1_A_nid_ORFs | ATGCCTTTGGTCGATGAATCTCAC | this work |
| Num1_A_nid_ORFa | GCGTTGCATCGATATCTACACCG | this work |

**References:**

1. Heimel K, Scherer M, Schuler D, Kämper J (2010) The *Ustilago maydis* Clp1 protein orchestrates pheromone and *b*-dependent signaling pathways to coordinate the cell cycle and pathogenic development. Plant Cell 22: 2908-2922.

2. Heimel K, Scherer M, Vranes M, Wahl R, Pothiratana C, et al. (2010) The transcription factor Rbf1 is the master regulator for *b*-mating type controlled pathogenic development in *Ustilago maydis*. PLoS Pathog 6: e1001035.
